# Evaluating sports nutrition advice provided by general-purpose large language models for recreational female marathon runners: a multidimensional descriptive audit

**DOI:** 10.3389/fnut.2026.1902377

**Published:** 2026-09-21

**Authors:** Hao Sun, Xinshuo Chu, Ziyang Li, Feng Wang

**Affiliations:** 1 School of Sports Training, Wuhan Sports University, Wuhan, China; 2 Jincheng College, Nanjing University of Aeronautics and Astronautics, Nanjing, China; 3 Physical Education Teaching Department, Xuanhua Vocational College of Science and Technology, Zhangjiakou, China

**Keywords:** female runners, LLMs, marathon, readability, safety boundaries, sports nutrition

## Abstract

**Background:**

General-purpose large language models (LLMs) are increasingly used for health and nutrition information. We evaluated the quality and practical limitations of LLM-generated sports nutrition advice for a standardized recreational female marathon runner, focusing on numerical recommendations, safety boundaries, and female-specific considerations.

**Methods:**

We conducted a time-stamped cross-sectional descriptive audit of five LLM configurations using 15 consumer-style prompts covering pre-race nutrition, in-race fueling and hydration, unexpected race situations, supplements, and female-specific nutritional risks. Each prompt was repeated 10 times per configuration, yielding 750 responses. The standardized profile was a 39-year-old, 58-kg recreational female marathon runner targeting a 4-h finish during the luteal phase. Responses were evaluated for scientific accuracy, numerical appropriateness and stability, safety-boundary handling, female-specific applicability, and readability. The coding framework was calibrated on 50 responses and finalized before blinded full-corpus evaluation by two author-raters and two independent external raters.

**Results:**

All configurations produced structured advice, but performance and female-specific integration varied by configuration, prompt, and domain. In the directly prompted Q15 scenario, ChatGPT covered all seven indicators in all 10 runs. Gemini's Q5 recommendations ranged from 5–6 gels and 50–60 g/h carbohydrate. DeepSeek and Doubao Q5 carbohydrate recommendations ranged from 15–120 g/h and 5–145 g/h, respectively, crossing both limits of the 30–90 g/h comparison range. One Doubao response stated a 115–145 g/h target while its gel schedule provided 37.5–45 g/h. Configurations differed in response stance toward strong pre-race coffee and in maintaining the training-first recommendation for new supplements (5/10–10/10). Model-level mean Flesch Reading Ease scores ranged from 45.5 to 57.8. Raw agreement between the independent experts was 91.24% for binary judgments (κ = 0.780); agreement with final author ratings was 87.76%−89.98% (κ = 0.703–0.763); exact numeric agreement was 75.0%; indicator-specific ICCs were 0.264–0.928.

**Conclusion:**

General-purpose LLMs can provide accessible and structured sports nutrition information for recreational female marathon runners, but their advice remains uneven in female-specific integration, numerical consistency, and safety-boundary communication. Apparent strengths were configuration-, prompt-, and context-specific and do not support a stable overall ranking of model families. LLM outputs may therefore be useful as supplementary information but should not replace individualized guidance from a sports dietitian or medical professional.

## Introduction

Regular running is associated with lower risks of all-cause, cardiovascular, and cancer mortality, and marathon training is also considered to have potential benefits for metabolic, skeletal muscle, and psychological health (1, 2). In recent years, marathon running has continued to expand in recreational fitness and urban sports events, with increasing overall participation and a growing proportion of female runners. A recent systematic review of the New York City Marathon showed that participation in this event increased markedly over time, with this growth mainly driven by increased participation among female and age-group runners (3). Data from the Berlin Marathon similarly showed that the number of female finishers increased from 8 in the inaugural race in 1974 to 12,268 in 2018 (4). However, as a prolonged endurance event, marathon running places substantial demands on pre-race nutritional preparation, in-race energy intake, hydration strategies, and gastrointestinal tolerance. Insufficient energy intake during the race may lead to fatigue, dizziness, and difficulty implementing the planned fueling strategy; therefore, prolonged endurance exercise requires appropriate planning of carbohydrate intake and fueling timing (5). At the same time, gastrointestinal symptoms are relatively common during marathon races, and their occurrence may be influenced by pre-race meal timing, energy gels, sports drinks, and hydration strategies (6, 7). With respect to hydration and fluid balance, inappropriate hydration strategies may increase the risk of exercise-associated hyponatremia (8). Exercise-associated muscle cramps are also common during marathon races (9, 10). In addition, NSAID use is not uncommon among marathon and ultramarathon runners, and its potential risks involve acute kidney injury, exercise-associated hyponatremia, and gastrointestinal complications (11, 12). For recreational female marathon runners, factors such as the menstrual cycle, iron status, low energy availability, and bone health may further increase the complexity of sports nutrition decision-making (13–17). Therefore, access to accurate, clear, and individually appropriate sports nutrition advice is particularly important for recreational female marathon runners during race preparation and competition.

With the development of generative artificial intelligence, general-purpose large language models (LLMs) have increasingly become a channel through which non-specialist users obtain health and nutrition information. A 2024 nationally representative survey in Australia showed that approximately one in ten adult respondents had used ChatGPT to obtain health information, with higher use reported among younger adults and those with lower health literacy (18). A previous systematic review showed that LLMs have been widely used in patient education and health information consultation contexts (19). In the field of nutrition, Ponzo et al. (20) found that different AI chatbots varied in the accuracy, completeness, reproducibility, and consistency of their nutrition advice. Solomon and Laye (21) further reported that generative AI chatbots have the potential to provide sports nutrition guidance, but the accuracy of their advice was at most moderate and performance varied across models. Previous studies have already evaluated test-retest reliability, safety, and readability in LLM-generated health and nutrition information, including comparisons between free and paid models (21–24). Accordingly, these domains are treated here as established evaluation dimensions. Therefore, LLM-generated responses may serve as a supplementary information source for recreational runners seeking sports nutrition information, but their scientific accuracy, safety, and context-specific applicability require further evaluation.

For recreational runners without specialized sports nutrition knowledge, it may be difficult to determine whether LLM-generated responses are scientifically accurate, contextually applicable, and safe. In particular, when model responses are presented in a clear and relatively confident manner, non-specialist users may be more likely to regard them as actionable guidance (25). Sports nutrition advice often involves specific intake amounts, fueling timing, and conditions of applicability; therefore, it requires not only complete information but also a high level of scientific accuracy and appropriate numerical recommendations. However, LLMs may generate inconsistent numerical plans or strategies across different sessions (21). For questions involving fueling doses and race-related decision-making, such output variability may affect how non-specialist users understand and implement the advice. Therefore, output stability should also be included in the evaluation. In addition, some sports nutrition decisions may involve potential risks. Whether a model can identify these risks and provide cautious advice also reflects its handling of safety boundaries. For recreational female marathon runners, factors such as the menstrual cycle, iron status, energy availability, and bone health may also influence nutritional requirements and risk assessment (13). Accordingly, the key unresolved question addressed in the present study is whether LLMs can recognize and substantively integrate these female-specific considerations within a standardized recreational marathon context. Recreational runners obtain LLM-generated responses not only to read information but also to translate advice into practical decisions. Previous studies have shown that the readability of health information can influence how non-specialist users understand and use such content (26, 27). Therefore, readability is also an important aspect of evaluating sports nutrition advice generated by LLMs. Because consumer-facing LLM outputs may depend on the specific model configuration and interface conditions, transparent reporting of the tested access route, account type, and interface mode is necessary for interpreting descriptive audits. In the present study, these factors were documented as characteristics of the tested configurations rather than treated as the basis for a paid-vs.-free comparison.

For these reasons, systematic and multidimensional evaluations of the quality of sports nutrition advice generated by general-purpose LLMs remain limited in the specific use context of recreational female marathon runners. Therefore, we developed a virtual profile of a recreational female marathon runner and designed standardized prompts covering pre-race nutritional preparation, in-race fueling, unexpected in-race situations, and supplement selection. These prompts were used to repeatedly evaluate five publicly accessible LLM configurations: ChatGPT, Gemini, Claude, DeepSeek, and Doubao, with their access routes, account types, and interface modes documented descriptively. The study focused on evaluating the content quality, scientific accuracy, appropriateness of numerical recommendations, output stability, safety boundaries, and sex- and population-specific applicability of the model responses. It further analyzed differences in readability, output format, and interaction style. The primary aim was to evaluate the potential and limitations of LLMs as auxiliary sports nutrition tools for recreational female marathon runners, focusing on their recognition and substantive integration of female-specific physiological and nutritional considerations; output stability, safety-boundary handling, and readability were assessed as complementary dimensions.

## Methods

### Study design

We conducted a cross-sectional, descriptive text audit of five general-purpose LLM configurations. A virtual participant profile and 15 standardized consumer-style prompts were used to examine four decision-making stages during marathon preparation and competition. Each prompt was submitted independently 10 times to each configuration, yielding 5 × 15 × 10 = 750 responses. All prompts and interactions were conducted in English to maintain language consistency and to reduce the influence of translation on formula-based readability estimates. No inferential testing or prespecified composite score was used; comparisons are descriptive.

Data were collected from April 10 to April 14, 2026, with one configuration tested per day through the developers' web interfaces in Microsoft Edge. Memory-control options were disabled where available (ChatGPT, Gemini, and Claude); no user-accessible memory-control option was available in the tested DeepSeek or Doubao interfaces. Previous conversation records were cleared, and every run used a newly opened conversation window. Responses were saved verbatim. The original log did not retain a time-zone field, prompt-order log, or randomization record; these omissions are acknowledged as limitations. Browsing, personalization, and other auxiliary interface functions were not independently controlled or recorded. Before readability analysis, Markdown and other formatting markers were normalized uniformly while substantive wording, numerical values, and existing punctuation were retained. Missing terminal punctuation was added only to independent list items and table rows to preserve their sentence boundaries.

### Model selection

We evaluated five publicly accessible web-interface configurations: ChatGPT (OpenAI, USA), Gemini (Google DeepMind, USA), Claude (Anthropic, USA), DeepSeek (DeepSeek, China), and Doubao (ByteDance, China). The access route and interface mode are reported because the configurations were not matched across vendors by model tier, computational level, or subscription condition.

Specific model versions and testing conditions were recorded from the information displayed in each web interface at the time of testing. The tested configurations were as follows: ChatGPT GPT-5.3 Instant (tested through a ChatGPT Plus account; April 10, 2026); Gemini 3 Flash (tested through a Google AI Pro account; April 11, 2026); Claude Haiku 4.5, with extended thinking disabled (tested through a Claude Pro account; April 12, 2026); DeepSeek-V3.2, with deep-thinking/R1 mode disabled (accessed through the public web interface without a paid subscription; April 13, 2026); and Doubao Seed 1.8, using the default “Fast” mode (accessed through the public web interface without a paid subscription; April 14, 2026). Extended-thinking, deep-reasoning, or other high-computation modes were not enabled for any model. However, the tested configurations were not matched across vendors by model tier or computational level. We did not use any additional prompt engineering, such as system instructions, role assignment, or formatting constraints. Each model generated responses solely based on the standardized user prompts designed in advance for this study.

### Virtual participant profile

We constructed a single virtual participant profile to represent a recreational female marathon runner. The participant was defined as a 39-year-old woman, 162 cm in height and 58 kg in body weight, with a body mass index of 22.1 kg/m^2^. She had 3 years of marathon running experience, a personal best of approximately 4 h, and race day was set in the luteal phase of her menstrual cycle, approximately 1 week before the expected onset of menstruation. The anthropometric and training characteristics were selected to approximate the ranges reported in previous studies of recreational endurance runners and marathon runners, rather than to represent a single mean value for any specific population. A Chinese marathon nutrition survey included 5,668 valid questionnaires, in which the mean age of female marathon runners was 41.0 ± 9.7 years (28). The NURMI study also reported similar characteristics among recreational endurance runners, with a mean age of 39 years and a median BMI of 21.72 kg/m^2^ (29). In addition, data from more than 70,000 Chinese half-marathon and marathon finishers showed that marathon participants included a substantial proportion of middle-aged adult runners (30). Therefore, 3 years of running experience and an approximate 4-h finishing goal were used to position the virtual participant as a recreational runner rather than an elite athlete.

The luteal-phase race-day scenario was included to construct a female-specific endurance running context, rather than a sex-neutral case. Although menstrual cycle phase may be related to metabolism, thermoregulation, gastrointestinal tolerance, fluid regulation, and perceived fatigue, evidence regarding its direct effects on exercise performance remains inconsistent (31, 32). Therefore, we treated the luteal phase as a contextual variable to evaluate whether LLMs could incorporate female-specific nutritional considerations.

### Prompt construction

We developed 15 standardized prompts covering four decision-making stages: T1, pre-race nutrition (Q1–Q4); T2, in-race fueling and hydration (Q5–Q7); T3, unexpected in-race situations (Q8–Q11); and T4, supplement selection and female-specific nutritional risks (Q12–Q15). Q4 was the explicit behavioral safety-boundary probe; Q14 was a knowledge-oriented test of whether the training-first principle was maintained. Q4 addressed a large strong coffee before the race, whereas Q14 addressed first use of a new supplement on race day. Q10 addressed early menstruation during the race as a female-specific scenario; NSAID-related content in Q10 was recorded only as an unprompted descriptive extension because the prompt did not mention pain, analgesics, or NSAIDs.

All prompts used direct, first-person, consumer-style wording. The fixed profile preamble was concatenated verbatim with each question; Supplementary material S1 provides the exact prompt components and concatenation rule, allowing each submitted prompt to be reconstructed verbatim. Because the preamble explicitly supplied the luteal-phase context in every prompt, mentions of the luteal phase were interpreted as prompt acknowledgment. Substantive physiological integration and unprompted identification of female-specific risks were coded separately. The direct Q15 scenario was therefore treated as a prompted coverage assessment, not as a test of spontaneous risk discovery.

### Evaluation framework

We used a written descriptive coding framework to assess content quality, scientific accuracy, numerical recommendations, output stability, female- and population-specific applicability, and safety boundaries. The framework was informed by prior LLM audits (20–24) and supplemented with prompt-specific reference criteria in Supplementary material S3. Detailed general and indicator-specific operational definitions, coding procedures, and the METRICS-oriented reporting checklist are provided in Supplementary material S4. All advice was judged against the target recreational female runner profile rather than an elite-athlete default.

Four authors contributed to the development of the framework. All had academic and research backgrounds in sports science and at least 2 years of research experience related to sports nutrition. Two authors (XC and FW) were postgraduate students and researchers in sports science who had received formal training in exercise physiology, sports nutrition, and sports science research methods. Their research experience included sports nutrition, athletic performance, and nutrition- and health-related issues relevant to female athletes. The other two authors (ZL and HS) were faculty members and researchers in sports science, with teaching and research experience in sports nutrition, athletic performance, and athlete health. All four authors had experience using and interpreting sports nutrition guidelines, consensus statements, and relevant scientific evidence. They were also familiar with the principal evidence-based recommendations underpinning the present evaluation framework, including those related to female athlete nutrition and health.

The coding framework was not preregistered. Before full coding, two authors (XC and FW) developed a preliminary framework and applied it to a small initial subset of responses (50) to assess its interpretability and practical application. This initial calibration exercise identified areas in which the coding rules required greater operational specificity. Another two authors (ZL and HS) subsequently refined the framework by clarifying inclusion and exclusion criteria, contextual scientific benchmarks, and rules for distinguishing response stance, factual accuracy, safety-information completeness, and substantive integration of topic-specific information. These refinements were intended to improve consistency and reduce ambiguity in the application of the framework across questions and models.

The revised framework was then finalized and frozen before formal duplicate coding of the full set of 750 responses. All responses, including those examined during the initial calibration exercise, were coded using the finalized framework. No coding rules were modified after full duplicate coding commenced. Supplementary material S8 compares the original and finalized evaluation frameworks and documents the operational changes.

Using the finalized operational definitions, two author-raters (XC and FW) independently coded all 750 responses, yielding 5,150 paired response-indicator records. We replaced response identifiers with blind IDs, and the two author-raters remained blinded to model identity and each other's ratings until coding was complete. We calculated inter-rater agreement from the original independent ratings before the two author raters resolved discrepancies by consensus under the same finalized rules to establish the final author ratings used in the study analyses. Supplementary material S6 provides the paired response-level coding sheets and agreement summary.

To provide independent validation of the finalized framework and response-level judgments, including those concerning scientific accuracy and safety, two independent external raters with expertise in sports nutrition each evaluated all 5,150 blinded records using the finalized definitions and prompt-specific benchmarks. Both had formal academic training and relevant research experience in exercise science and sports nutrition. Neither was an author, and neither had participated in framework development, refinement, or coding by the two authors. Each received a workbook with an independently randomized response order and was blinded to model identity and version, the two authors' ratings, model-level study results, and the other expert's ratings. The experts did not discuss responses or coding decisions before completing their workbooks and did not add or modify any coding rules. We calculated agreement from their original independent ratings without consensus adjustment. Supplementary material S7 provides the complete coding sheets and agreement summary.

Content quality included clarity of language, depth of information, structural organization, and supplementary information. Scientific accuracy included correctness, contextual numerical appropriateness, internal consistency, and plan rationality. Error categories were: (1) hard scientific error, a clear violation of established consensus; (2) outside an applicable reference benchmark, a value outside a context-relevant comparison range without implying a hard error; (3) internal contradiction, incompatible claims or targets within one response; and (4) key-information omission, absence of a specified coding element relevant to the prompt. The complete prompt-specific matrix, target context, units, time scale, borderline rules, and sources are provided in Supplementary material S3. Q1 used 10–12 g/kg/day as a race-focused target for the final 36–48 h, while lower values within the 3-day plan were interpreted contextually. Q5 carbohydrate values below 30 g/h and above 90 g/h were classified symmetrically as outside the applicable comparison range. No universal fixed hourly sodium ceiling was applied; Q7 sodium was interpreted using sweat rate, sweat sodium concentration, fluid balance, environmental conditions, race duration, and individual tolerance. Exceeding a comparison range did not automatically constitute a hard scientific error.

Stability was evaluated across 10 runs per prompt using core-consistency frequencies and the observed minimum-to-maximum span of key numerical outputs. The span was treated as a descriptive range, not as a robust dispersion estimate. Female-specific applicability was assessed by distinguishing prompt acknowledgment, substantive physiological integration, and coverage of prompted risk indicators. Safety-boundary handling was coded across separate elements: risk recognition, dose or prior-tolerance assessment, recommendation against untested strategies, adverse-symptom recognition, stopping or adjustment advice, and the scientific appropriateness of conditional advice. Q4 was the explicit behavioral safety-boundary probe; Q14 was a knowledge-oriented test of whether the training-first principle was maintained. Q10 NSAID mentions were recorded only as unprompted descriptive extensions. Clarifying-question responses were retained in the denominator; indicators not present in such responses were coded as absent, and their full text remained in the readability dataset.

### Readability analysis

We used the WebFX Readability Test Tool (https://www.webfx.com/tools/read-able/; accessed April 2026) to assess the processed texts derived from all 750 responses. Seven indices were extracted: Flesch Reading Ease (FRE), Flesch–Kincaid Grade Level (FKGL), Gunning Fog Score, Simple Measure of Gobbledygook (SMOG) Index, total number of sentences, total word count, and mean words per sentence. Formula-based readability metrics have been widely used in studies of patient and consumer health information (33) and have also been adopted in recent evaluations of LLM-generated health information (22, 34). FRE ranges from 0 to 100, with higher scores indicating greater readability (35). FKGL estimates the US school grade level required to understand a text (36). The Gunning Fog Score estimates reading difficulty based on sentence length and the use of complex words (37), whereas the SMOG Index estimates the years of education required to understand a text based on the density of polysyllabic words (38). For interpretation, higher FRE scores and lower FKGL, Gunning Fog, and SMOG values indicate greater readability. Sentence count, word count, and mean words per sentence were used as descriptive text-structure indicators to support interpretation of the formula-based readability results. Health materials intended for patients and general users are typically recommended to be written at approximately a US sixth- to eighth-grade reading level to improve accessibility for general users (27).

### Statistical analysis

We used descriptive analysis only. For categorical indicators, frequencies and proportions were calculated across the 10 runs for each model and prompt, with all 10 runs retained in the denominator, including clarifying-question responses. For numerical recommendations, observed minimum-to-maximum ranges described the span of outputs; because many responses contained intervals or conditional alternatives, medians, interquartile ranges, and coefficients of variation were not retrospectively imposed on the coded content variables. Readability was summarized across all 750 processed responses using model-level means, standard deviations, ranges, medians, and interquartile ranges. No inferential tests, confidence intervals, hypothesis tests, or prespecified composite score were used. Accordingly, model comparisons are descriptive, prompt-specific, and domain-specific rather than ordinal rankings. We separately analyzed the reproducibility of the two authors' coding, the agreement between the independent experts, and the concordance of each independent expert with the final author ratings used in the primary analyses. For each paired evaluation, raw percent agreement was calculated for the binary indicators and the three-category Q4 response-stance variable. Cohen's κ was calculated for the same indicators. Author–expert concordance was calculated for all binary indicators and separately for the scientific-accuracy-related and safety-boundary subsets identified from the finalized operational definitions. For numeric fields, exact agreement was calculated across all paired records, and two-way random-effects, absolute-agreement, single-measure ICCs [ICC(2,1)] were calculated separately for each indicator when both evaluators recorded numeric values. Jointly coded “not stated” entries contributed to exact agreement but were excluded from ICC calculations. ICCs were not pooled because the numeric indicators differed in units and scales. Agreement between the two authors was calculated from their original independent ratings before author reconciliation, whereas agreement between the independent experts was calculated from their original ratings without consensus adjustment. Discrepancies between the two authors were then resolved by consensus to establish the final author ratings used in the study analyses, and these ratings were used to calculate author–expert concordance. Records for which both independent experts assigned the same classification and both differed from the final author rating were reviewed against the original response text and the finalized operational definitions, and their potential effects on the reported results and conclusions were assessed. For the two numeric indicators with the lowest ICCs, discrepant expert values were also reviewed at the response level to characterize possible context- or time-scale-related sources of extraction disagreement; the original expert ratings were not modified.

Readability indicators were summarized across the processed texts derived from all 750 responses. Mean, standard deviation, and range are reported in Table 1; medians and interquartile ranges are reported in Supplementary material S2; and prompt-stratified means are provided in Supplementary Table S2.2. The kernel-density figure displays individual responses and model means without inferential confidence intervals. Because responses were clustered within prompts, the model-level summaries are descriptive; future inferential work should use mixed-effects models with prompt as a random effect.

**Table 1:** Readability and text-structure metrics by model.

Model	FRE	FKGL	Gunning fog	SMOG	Sentences	Words	Words/sentence
ChatGPT	56.6 (10.0), 22.3–75.3	8.6 (2.1), 4.9–15.0	11.0 (2.0), 7.9–16.7	8.3 (1.6), 6.0–12.9	35.7 (12.8), 11–87	435.6 (118.9), 116–734	13.0 (4.0), 6.6–23.9
Gemini	53.1 (7.4), 30.2–69.7	9.8 (1.3), 6.9–13.8	11.7 (1.4), 9.1–15.7	9.1 (1.0), 7.0–12.3	30.6 (4.6), 10–42	480.7 (55.8), 178–613	15.9 (1.7), 12.2–20.3
Claude	45.5 (9.9), 18.3–74.1	11.3 (1.9), 6.1–15.9	13.8 (2.0), 8.5–19.4	10.3 (1.5), 6.6–14.4	30.5 (9.1), 7–65	523.7 (125.1), 193–830	17.7 (3.0), 11.1–27.6
DeepSeek	57.8 (8.5), 27.8–76.5	8.7 (1.5), 5.0–13.5	11.2 (1.6), 7.4–17.2	8.4 (1.2), 5.7–12.1	41.2 (12.5), 12–76	568.8 (145.3), 201–922	14.2 (2.4), 9.7–22.9
Doubao	54.1 (11.8), 24.8–85.6	9.8 (2.4), 4.0–15.4	11.8 (2.3), 5.8–17.4	9.1 (1.8), 5.0–13.9	26.2 (10.9), 6–61	393.6 (109.9), 86–697	16.4 (4.5), 6.8–24.9

Values are mean (SD), range across 150 responses per model.

FRE, Flesch Reading Ease; FKGL, Flesch–Kincaid Grade Level; SMOG, Simple Measure of Gobbledygook. Higher FRE scores and lower FKGL, Gunning Fog, and SMOG values indicate greater readability.

## Results

Independent expert validation covered all 5,150 response-indicator records. Using the experts' original ratings without consensus adjustment, agreement across 4,500 binary judgments was 91.24% (Cohen's κ = 0.780), and agreement across 50 three-category Q4 response-stance judgments was 84.0% (κ = 0.481). Across the 4,500 binary records, agreement with the final author ratings used in the primary analyses was 87.76% for Expert A (κ = 0.703) and 89.98% for Expert B (κ = 0.763). For the 650 scientific-accuracy-related records, agreement was 80.46% with Expert A (κ = 0.547) and 84.62% with Expert B (κ = 0.651); for the 1,200 safety-boundary records, agreement was 84.00% with Expert A (κ = 0.524) and 84.42% with Expert B (κ = 0.583). Both experts agreed with each other but differed from the final author rating in 304 of 4,500 binary records (6.76%), including 70 of 650 scientific-accuracy-related records (10.77%) and 125 of 1,200 safety-boundary records (10.42%).

Between the two experts, exact agreement across 600 numeric extraction records was 75.0%. Among records for which both experts entered numeric values, indicator-specific ICC(2,1) values ranged from 0.264 to 0.928. The lowest ICCs were observed for Q7 sodium intake maximum (0.264) and Q1 carbohydrate target minimum (0.384); response-level audits indicated that discrepancies often involved the numeric context or time scale selected for Q7 sodium and the stage selected from multistage carbohydrate-loading plans for Q1. Supplementary materials S7, S9, and S10 provide the original expert coding and agreement summary, the author–expert concordance and joint-disagreement assessment, and all 12 indicator-specific ICCs and low-reproducibility audits, respectively.

As shown in Table 2, all five configurations generated pre-race plans covering carbohydrate loading, race-day breakfast, pre-race supplements, and strong coffee use, with no hard scientific errors detected. Gemini had the most conservative Q4 response stance, with no endorsement in any of the 10 runs, but it also provided no caffeine-dose guidance in Q4 (0/10). Claude showed conditional endorsement in 6/10 runs and no endorsement in 4/10 runs, whereas ChatGPT and DeepSeek showed conditional endorsement and Doubao showed endorsement in all 10 runs. DeepSeek's Q1 carbohydrate recommendations spanned 6–12 g/kg/day across runs. Doubao covered iron in 10/10 runs in Q3, whereas the luteal-sodium link was present in 2/10 runs.

**Table 2:** Comparative analysis of LLM-generated plans under pre-race nutrition preparation.

Dimension	ChatGPT (GPT-5.3 Instant)	Gemini (Gemini 3 Flash)	Claude (Haiku 4.5)	DeepSeek (V3.2)	Doubao (Seed 1.8)
Content quality					
Language quality	Simple-to-detailed; conversational openings; emoji-segmented	Academic-professional; occasional formulas; table-based	Structured; occasionally asks clarifying questions before answering (Q1 3/10; Q2 4/10; Q3 2/10)	Simple-to-detailed; summary-then-breakdown; mechanism-focused exposition	Direct to points; short headed paragraphs; templated structure
Depth of information	Q1 carbs: 6–11 g/kg/day; Q3 caffeine/nitrate/β-alanine 10/10	Q1 carbs: 7–12 g/kg/day; Q3 top-3 supplements 10/10	Q1 carbs: 8–12 g/kg/day in the main 3-day race-week loading recommendations; Q3 caffeine 10/10, nitrate 5/10, β-alanine 7/10; iron 7/10; RED-S 5/10	Q1 carbs: 6–12 g/kg/day; Q1 luteal-phase mechanism covered progesterone, insulin, and gastric emptying	Q1 carbs: 7–12 g/kg/day; Q3 iron 10/10
Structural clarity	Multi-level sections; numbered headings; concluding summary	Tables and day-by-day timelines; bullet points	Hierarchy; timeline tables; closed by “Final Tips”	Six numbered sections; quantitative data in tables	Short headed paragraphs; recurring template across runs
Additional content	Sample menus 10/10; proactively offers to create full meal plan	Sample menus 10/10; “Pro Tip” boxes; tables	Sample menus 10/10; day-by-day tables; web-searched for Q3 on some runs	Sample menus 10/10; food-carb quantification tables; offers follow-up	Sample menus 10/10.
Scientific accuracy					
Scientific accuracy	Q1 carbohydrate recommendations ranged from 6 to 11 g/kg/day; Q4 caffeine dose guidance 5/10	Q1 carbohydrate recommendations ranged from 7 to 12 g/kg/day; Q3 top-3 supplements 10/10	Q1 recommendations ranged from 8 to 12 g/kg/day across runs; Q3 iron 7/10	Q1 recommendations varied from 6–7 to 10–12 g/kg/day across runs	No outside-benchmark Q1 recommendation was detected in the main 3-day race-week loading recommendation
Error cases (out of 10)	Hard scientific errors: none detected Outside reference benchmark: none detected for Q1 Key omissions: Q3 iron 4/10; Q3 female-specific risk (RED-S/bone density) 0/10	Hard scientific errors: none detected Outside reference benchmark: none detected for Q1 Key omissions: Q3 iron 0/10; Q3 RED-S 0/10; Q4 caffeine dose guidance 0/10	Hard scientific errors: none detected Outside reference benchmark: none detected in the main 3-day race-week loading recommendation Key omissions: Q3 nitrate 5/10	Hard scientific errors: none detected Outside reference benchmark: none detected for Q1 Key omissions: Q3 nitrate 1/10 (mentioned only once in 10 runs); Q3 RED-S 0/10	Hard scientific errors: none detected Outside reference benchmark: none detected in the main 3-day race-week loading recommendation Key omissions: Q3 nitrate 2/10; Q3 RED-S 0/10
Plan rationality	Sample meals 10/10	Included quantitative recommendations.	Included reminders to test the strategy in training	Recommendations were organized into six sections	Used a recurring plan template across runs
Stability (across 10 runs)					
Core recommendation consistency	8/10. A day-specific lower bound of 6 g/kg/day appeared in 2 runs	9/10. Q1 carbs anchored at 7–10 or 8–10 g/kg/day; reached 12 g/kg/day once	8/10. Luteal-phase stance differed in 2 runs	7/10. Q1 recommendations ranged across 6–7, 8–9, and 10–12 g/kg/day	6/10. Q1 luteal-phase conclusions differed across runs
Variability in key numerical values	Q1 carbs range 5 g/kg/day (6–11)	Q1 carbs range 5 g/kg/day (7–12)	Q1 carbs range 4 g/kg/day (8–12)	Q1 carbs range 6 g/kg/day (6–12)	Q1 carbs range 5 g/kg/day (7–12)
Sex- and population-specific applicability					
Coverage of female-specific risks	Luteal mentions 40/40; Q1 luteal-sodium link 5/10 Female-specific risks: Q3 iron 4/10; RED-S 0/10	Luteal mentions 40/40; Q1 luteal-sodium link 10/10 Female-specific risks: Q3 iron 0/10; RED-S 0/10	Luteal mentions 40/40; Q1 luteal-sodium link 6/10 Female-specific risks: Q3 iron 7/10; RED-S 5/10	Luteal mentions 40/40; luteal-sodium link 9/10 Female-specific risks: Q3 iron 5/10; RED-S 0/10	Luteal mentions 40/40; luteal-sodium link 2/10 Female-specific risks: Q3 iron 10/10; RED-S 0/10
Applicability to recreational female runners	Daily-use sample menus 10/10	Included formulas and technical terms	Formal language; clarifying questions occurred in Q1 3/10, Q2 4/10, and Q3 2/10	Six-section structure with quantitative tables	Short headed paragraphs with a recurring template
Safety					
Q4 response stance and safety-information provision	Stance: conditional endorsement 10/10. Safety information: warnings 9/10; GI 10/10; heart rate/anxiety 10/10; dehydration 7/10; dose guidance 5/10; empty-stomach risk 7/10	Stance: no endorsement 10/10. Safety information: warnings 9/10; GI 10/10; heart rate/anxiety 10/10; dehydration 9/10; dose guidance 0/10; empty-stomach risk 5/10	Stance: conditional endorsement 6/10; no endorsement 4/10. Safety information: warnings 9/10; GI 10/10; heart rate/anxiety 10/10; dehydration 6/10; dose guidance 3/10; empty-stomach risk 9/10	Stance: conditional endorsement 10/10. Safety information: warnings 10/10; GI 10/10; heart rate/anxiety 10/10; dehydration 6/10; dose guidance 7/10; empty-stomach risk 9/10	Stance: endorsement 10/10. Safety information: warnings 10/10; GI 10/10; heart rate/anxiety 9/10; dehydration 10/10; dose guidance 3/10; empty-stomach risk 8/10
Overall					
Key characteristics	Conversational openings; sample menus 10/10; core recommendation consistency 8/10	Academic-professional language; table-based; Q4 no endorsement 10/10 and caffeine-dose guidance 0/10	Q3 iron 7/10; RED-S 5/10; clarifying questions occurred in Q1 3/10, Q2 4/10, and Q3 2/10	Q1 mechanism discussion included progesterone, insulin, and gastric emptying; luteal-sodium link 9/10; core recommendation consistency 7/10	Short headed paragraphs with a recurring template; core recommendation consistency 6/10; luteal-sodium link 2/10; Q3 iron 10/10; RED-S 0/10
Strengths	Sample menus 10/10; core recommendation consistency 8/10	Q4 gastrointestinal and heart-rate/anxiety warnings 10/10; luteal-sodium link 10/10	Q3 iron 7/10; RED-S 5/10	Q1 luteal-phase mechanism covered progesterone, insulin, and gastric emptying; luteal-sodium link 9/10	Q3 iron 10/10
Limitations	Q1 luteal-sodium link 5/10; RED-S 0/10; Q4 warnings 9/10 and conditional endorsement 10/10	Q3 iron 0/10; Q4 dose guidance 0/10	Clarifying questions occurred in Q1 3/10, Q2 4/10, and Q3 2/10. Luteal-phase stance varied across runs	Core recommendation consistency 7/10; Q1 recommendations ranged from 6 to 12 g/kg/day; Q3 nitrate 1/10	RED-S 0/10; opposite conclusions on luteal-phase carbohydrate adjustment occurred across runs

Questions Covered: Q1–Q4 (3-day carb loading; race-day breakfast; pre-race supplements; strong black coffee). Data source: 5 models × 4 questions × 10 runs = 200 responses.

Error Classification: (1) hard scientific errors—clearly violating scientific consensus; (2) outside reference benchmark—outside the referenced benchmark range but not necessarily a hard scientific error; (3) internal contradictions; (4) key-information omissions. Prompt-specific reference parameters, applicability conditions, classification rules, and supporting sources are provided in Supplementary material S3.

As shown in Table 3, the five models showed more pronounced differences in numerical precision, stability, and safety boundaries for in-race fueling. Under the tested web-interface conditions, Gemini showed the least variation in its Q5 numerical recommendations, with energy-gel recommendations ranging from 5 to 6 gels and carbohydrate recommendations ranging from 50 to 60 g/h. ChatGPT's Q5 recommendations ranged from 5 to 10 energy gels and from 30 to 90 g/h of carbohydrate, while physiological explanation of the luteal-phase context was limited. Claude's Q5 energy-gel recommendations ranged from 4 to 12 gels, with 10–12 gels appearing in three of the 10 runs. This variability should be interpreted in relation to carbohydrate content per gel, timing, and individual tolerance. DeepSeek provided detailed

**Table 3:** Comparative analysis of LLM-generated plans under in-race nutrition supplementation.

Dimension	ChatGPT (GPT-5.3 Instant)	Gemini (Gemini 3 Flash)	Claude (Haiku 4.5)	DeepSeek (V3.2)	Doubao (Seed 1.8)
Content quality					
Language quality	Simple-to-detailed; conversational openings; emoji-segmented	Academic-professional; includes pace/math expressions; table-based	Structured; begins with energy-expenditure calculation; ends with personalization question	Summary first, followed by mechanism-focused exposition	Templated “Your Marathon Fuel Plan” openings; short bulleted paragraphs; recurring structure across runs
Depth of information	Q5 gels: 5–10; Q5 carbs/hr: 30–90 g/h; Q6 all 3 mechanisms (blood glucose/glycogen/absorption) 10/10; Q7 fluids 400–800 mL/h, sodium 200–700 mg	Q5 gel recommendations ranged from 5 to 6; carbohydrate recommendations ranged from 50 to 60 g/h; pre-race gel and the luteal-sodium link were each present in 10/10 runs	Q5 gel recommendations ranged from 4 to 12; carbohydrate recommendations ranged from 30 to 90 g/h; responses included kcal and glycogen estimates	Q5 gel recommendations ranged from 4 to 10, carbohydrate recommendations ranged from 15 to 120 g/h, and sodium recommendations ranged from 200 to 3,000 mg, with one run reporting a race-total estimate	Q5 gels: 4–8; Q5 carbohydrate: 5–145 g/h; Q7 sodium recommendations varied across runs, with 800–1,200 mg/h recommended in 2/10 runs
Structural clarity	Multi-level sections; numbered+emoji subtitles; “Bottom line” ending	Timetables and “Pro Tip” boxes; dual mile/km units	Hierarchy; timing tables with alternative plans; ends with clarifying question	Multi-section numbering, tables, and timelines	Fixed “Key Context/Recommended Plan/Why/Tips” sequence
Additional content	Timing schedules; proactively offers minute-level customization	Timetables; names specific gel brands (Maurten/GU/SIS); Pro Tip sections	Kcal calculations; ends with questions about past experience and gel-brand preferences	Contrasts multiple fueling strategies (gel-only vs. gel+sports drink)	Mile/km timing tables
Scientific accuracy					
Scientific accuracy	Q5 carbohydrate recommendations ranged from 30 to 90 g/h within the applicable reference benchmark; Q6 blood glucose 9/10, glycogen 10/10, and absorption 10/10; Q7 addresses hydration and sodium intake	Q5 gel recommendations ranged from 5 to 6 and carbohydrate recommendations from 50 to 60 g/h; Q7 luteal+progesterone mechanism 10/10	Q5 included kcal and glycogen-store estimates; 10–12 gels appeared in 3/10 runs; Q7 hyponatremia warning 10/10	Q7 luteal+progesterone mechanism 10/10; Q5 carbohydrate recommendations ranged from 15 to 120 g/h, crossing both the lower and upper limits of the applicable 30–90 g/h reference benchmark	Q5 carbohydrate recommendations ranged from 5 to 145 g/h, crossing both limits of the 30–90 g/h benchmark. Run 2 stated 2.0–2.5 g/kg/h (≈115–145 g/h). Q7 luteal+progesterone mechanism 3/10
Error cases (out of 10)	Hard scientific errors: none detected Outside reference benchmark: none detected for parameters with an applicable comparison range Key omissions: Q5 pre-race gel 1/10; Q5 luteal-sodium link 1/10	Hard scientific errors: none detected. Outside reference benchmark: none detected Key omissions: none listed	Hard scientific errors: none detected. Outside reference benchmark: not assigned from gel count alone; 10–12 gels appeared in 3/10 runs Key omissions: gel carbohydrate content and timing were not consistently specified	Hard scientific errors: none detected. Outside reference benchmark: Q5 carbohydrate recommendations crossed both limits of the 30–90 g/h comparison range (15–120 g/h) Internal contradictions: none clearly detected. Q7 sodium was not scored against a universal fixed hourly threshold Key omissions: none listed	Hard scientific errors: none detected. Outside reference benchmark: Q5 range 5–145 g/h crossed both limits (< 30 and >90 g/h). Q7 sodium was not scored against a universal fixed hourly threshold Internal contradictions: one Q5 response stated a 115–145 g/h target while its accompanying energy-gel schedule provided 37.5–45 g/h Key omissions: Q5 caffeine gel 0/10; Q5 pre-race gel 0/10; Q5 luteal-sodium link 1/10
Stability (across 10 runs)					
Plan rationality	Example timings were provided	Recommendations named specific gel brands and included “Pro Tip” sections	Responses included clarifying questions	Presented multiple alternative fueling strategies	Run 2 numerical target contradicted the actual gel plan
Core recommendation consistency	8/10. Q5 gel count varies 5–10; Q5 carbs/hr consistent 30–90 g/h	10/10. Q5 gels stable 5–6; Q5 carbohydrate intake: 50–60 g/h; Q7 consistent	6/10. Q5 gel count fluctuates 4–12; some runs (5/7/10) recommend 10–12	7/10. Q5 gels fluctuate 4–10; Q7 sodium spans 200 to 3,000 mg (includes race-total figures)	6/10. Q5 carbs/hr target spans 5–145 g/h; Run 2 differed from other runs
Variability in key numerical values	Q5 gels range 5 (5–10); Q5 carbs/hr range 60 (30–90 g/h)	Q5 gels range 1 (5–6); Q5 carbs/hr range 10 (50–60 g/h)	Q5 gels range 8 (4–12); Q5 carbs/hr range 60 (30–90 g/h)	Q5 gels range 6 (4–10); Q5 carbs/hr range 105 (15–120 g/h)	Q5 gels range 4 (4–8); Q5 carbs/hr range 140 (5–145 g/h)
Sex- and population-specific applicability					
Coverage of female-specific risks	Luteal mentions 30/30; Q7 luteal+progesterone mechanism 0/10	Luteal mentions 30/30; Q7 luteal+progesterone mechanism 10/10; Q5 luteal-sodium link 10/10	Luteal mentions 30/30; Q7 luteal+progesterone mechanism 2/10	Luteal mentions 30/30; Q7 luteal+progesterone mechanism 10/10	Luteal mentions 30/30; Q7 luteal+progesterone mechanism 3/10
Applicability to recreational female runners	Example timings were provided	Responses included pace conversions and calculations	Formal language	Multiple fueling strategies were presented	Templated responses
Safety					
Safety-boundary handling	Q6 rejects “wait for fatigue” 9/10; Q7 “water alone insufficient” 10/10; Q7 hyponatremia warning 10/10	Q6 rejects 7/10; Q7 hyponatremia warning 10/10; Q7 sodium recommendations ranged from 500 to 700 mg/h	Q6 rejects 6/10; Q7 hyponatremia warning 10/10; Q7 sodium 300–700 mg/h	Q6 rejects 10/10; Q7 hyponatremia warning 10/10; but Q7 sodium 200–3,000 mg range (race-total vs. hourly ambiguity)	Q6 rejects 10/10; Q7 hyponatremia warning 10/10
Overall					
Key characteristics	Core recommendation consistency 8/10; Q5 pre-race gel 1/10; Q5 luteal-sodium link 1/10	Q5 gel recommendations ranged from 5 to 6 and carbohydrate recommendations from 50 to 60 g/h; core recommendation consistency 10/10; Q7 luteal+progesterone mechanism 10/10	Q5 gel recommendations ranged from 4 to 12; core recommendation consistency 6/10; responses included clarifying questions	Core recommendation consistency 7/10	Templated responses; Q5 carbohydrate recommendations ranged from 5 to 145 g/h; core recommendation consistency 6/10
Strengths	Q6 glucose mechanism 9/10, glycogen mechanism 10/10, and absorption mechanism 10/10; Q7 “water alone insufficient” 10/10 and hyponatremia warning 10/10	Q5 gel recommendations ranged from 5 to 6; Q5 luteal-sodium link 10/10; Q5 pre-race gel 10/10; Q7 luteal+progesterone mechanism 10/10	Q5 includes kcal calculations; individualized guidance for luteal-phase GI sensitivity; proactive personalization questions	Q6 rejects fatigue-triggered fueling 10/10; Q7 luteal+progesterone mechanism 10/10	Q6 rejects fatigue-triggered fueling 10/10; Q7 dehydration warning 10/10
Limitations	Q5 pre-race gel 1/10; Q5 luteal-sodium link 1/10; Q7 luteal+progesterone mechanism 0/10	Q6 rejects fatigue-triggered fueling 7/10	Q5 gel recommendations ranged from 4 to 12; Q6 rejects fatigue-triggered fueling 6/10; Q5 caffeine gel 0/10	Q5 carbs/hr ceiling 120 g (single run) exceeds the referenced upper benchmark of 90 g/h; Q7 sodium race-total vs. hourly ambiguity	Q5 carbohydrate recommendations ranged from 5 to 145 g/h, crossing both limits of the 30–90 g/h comparison range. One response stated a 115–145 g/h target while its energy-gel schedule provided 37.5–45 g/h

Questions Covered: Q5–Q7 (energy gel count; fueling rhythm; 25 °C hydration strategy). Data source: 5 models × 3 questions × 10 runs = 150 responses.

Error Classification: (1) hard scientific errors—clearly violating scientific consensus; (2) outside reference benchmark—outside the referenced benchmark range but not necessarily a hard scientific error; (3) internal contradictions; (4) key-information omissions. Prompt-specific reference parameters, applicability conditions, classification rules, and supporting sources are provided in Supplementary material S3.

physiological rationale, but its Q5 carbohydrate recommendations ranged from 15 to 120 g/h, crossing both the lower and upper limits of the 30–90 g/h reference benchmark, while the 3,000 mg sodium value in Q7 was closer to an estimated whole-race total than a per-hour recommendation. Doubao showed an even wider Q5 carbohydrate range of 5–145 g/h, likewise crossing both limits of the reference benchmark. In one Q5 response, the stated target of 115–145 g/h was internally inconsistent with the accompanying energy-gel schedule, which provided only 37.5–45 g/h. In Q7, Doubao recommended a sodium intake of 800–1,200 mg/h in 2 of 10 runs.

As shown in Table 4, no hard scientific errors were detected in the five configurations for the unexpected in-race scenarios. Differences were observed in completeness, emergency decision pathways, and safety emphasis. Doubao generated the shortest Q9 responses and omitted several emergency-management elements. In Q10, spontaneous NSAID-related kidney-risk mentions occurred in Gemini (10/10), DeepSeek (8/10), and ChatGPT (5/10), but not in Claude or Doubao (0/10); these were descriptive unprompted extensions, not safety-boundary scores. Gemini also sometimes described menstruation onset as a transition toward the follicular phase; because evidence for phase-related performance effects is inconsistent, this observation was not treated as a strength. For Q11, DeepSeek provided a detailed integrated rationale covering dehydration, electrolytes, neuromuscular factors, and the luteal-phase context.

**Table 4:** Comparative analysis of LLM-generated plans under in-race emergency situations.

Dimension	ChatGPT (GPT-5.3 Instant)	Gemini (Gemini 3 Flash)	Claude (Haiku 4.5)	DeepSeek (V3.2)	Doubao (Seed 1.8)
Content Quality					
Language quality	Simple-to-detailed; emergency steps as checklists; emoji-segmented with “Bottom line” closure	Academic; uses medical terminology (prostaglandins/aldosterone/plasma volume); table-based	Structured; mechanism explanation and decision tree (“if X, then Y”)	“Short answer” first, followed by sections and multiple alternative comparisons	Templated structure; short paragraphs and multi-level headings
Depth of information	Q10 sometimes described a luteal-to-follicular transition; because evidence for phase-related performance effects is inconsistent, this was not treated as a strength. Q11 progesterone link 6/10	Q8 stop-solid-gel 8/10; Q10 described menstruation as a follicular-phase shift (“power phase”); evidence for phase-related performance effects is inconsistent; Q11 progesterone link 6/10	Q8 continue-cautious 10/10; Q9 underfuel-risk 5/10; Q10 iron-not-acute 10/10; Q11 dehydration mechanism 8/10	Q8 stop-solid-gel 9/10; Q8 pace-adjustment 8/10; Q11 progesterone-luteal cramp link 10/10; Q11 magnesium 10/10; Q11 neuromuscular mechanism 9/10	Q9 hot-weather factor 1/10; Q11 dehydration mechanism 2/10
Structural clarity	Multi-level headings+emoji segments; emergency steps as checklists	Q10 included a “luteal vs. follicular” comparison table; numbered sections	Hierarchy; “if-then” decision logic; ends with clarifying questions	Short answer, multi-section structure, and comparison tables	Templated structure; short paragraphs; Q9 mean response length was 327.4 words
Additional content	Proactively offers to personalize for “4-h goal + luteal phase” plan	Q10 includes luteal vs. follicular physiological comparison table; Q11 includes nutrient function table (Mg/Ca/anti-inflammatories)	Q8 tiered responses for mild/moderate/severe GI distress; ends with questions about past experience	Q11 discussion addressed neuromuscular, electrolyte, and dehydration mechanisms	Emergency-flow descriptions were provided
Scientific accuracy					
Scientific accuracy	Q10 sometimes described a luteal-to-follicular transition; evidence for phase-related performance effects is inconsistent, so this was not classified as a strength. Q11 progesterone link 6/10	Q10 framed menstruation onset as a shift into the follicular phase and discussed glycogen access; Q11 progesterone-luteal cramp link 6/10; Q10 carbohydrate recommendations ranged from 60 to 90 g/h	Q10 “no major change” was explicit in 4/10 runs; Q11 magnesium 8/10	Q11 discussion addressed dehydration, electrolyte, and neuromuscular mechanisms	Q9 hot-weather factor 1/10 and underfuel-risk 4/10; Q11 neuromuscular mechanism 1/10
Error cases (out of 10)	Hard scientific errors: none detected Outside reference benchmark: none detected Key omissions: Q11 magnesium 0/10; Q8 pace-reduction 4/10 and DNF option 1/10	Hard scientific errors: none detected Outside reference benchmark: none clearly detected; however, Q10 maintained 60–90 g/h carbohydrate intake in some runs, within the referenced benchmark. Internal contradictions: none detected Key omissions: Q10 sanitary products 0/10 (does not address physical menstrual management during race); Q10 “no new on race day” principle only 3/10	Hard scientific errors: none detected Outside reference benchmark: none detected Key omissions: Q9 hot-weather factor 2/10	Hard scientific errors: none detected Outside reference benchmark: none detected Key omissions: Q9 pause-and-assess dimension 0/10 (jumps directly to reduce/switch without suggesting observation pause); Q9 underfuel-risk only 1/10	Hard scientific errors: none detected Outside reference benchmark: none detected Internal contradictions: none detected Key omissions: Q9 hot-weather factor 1/10, Q9 no-force principle 1/10, Q11 dehydration mechanism 2/10, Q11 neuromuscular mechanism 1/10, and Q10 sanitary products 0/10
Plan rationality	Emergency steps were presented as checklists	Recommendations were organized around physiological mechanisms	“If-then” logic covered different severity levels; responses included clarifying questions	Responses included multiple alternative paths for emergency scenarios	Templated responses
Stability (across 10 runs)					
Core recommendation consistency	8/10. Q10 explicit “no major change” only 3/10, but core advice (maintain carbs/emphasize electrolytes) consistent across runs	10/10. Core recommendations highly consistent across runs for all sub-questions; Q10 consistently frames menstruation as “luteal to follicular shift”	8/10. Q10 core stance consistent (“no major change”); but Q8 response granularity varies (mild/moderate/severe tiering omitted in some runs)	7/10. Q10 progesterone mechanism in 9/10 runs, but “no major change” stance in 0/10 explicit (default leans toward “adjust”); Q11 mechanism explanations consistent	7/10. Templated structure consistent, but information coverage varies across runs
Variability in key numerical values	Q10 did not quantify additional caffeine intake in mg; Q11 did not specify carbohydrate amounts in grams for crampmanagement	Q10 carbohydrate recommendations ranged from 60 to 90 g/h	Q11 magnesium dose was not quantified.	Q11 specific quantities, including magnesium mg and pickle juice mL, varied across runs	Q9 used qualitative amounts such as “small amount” and “slowly” in emergency advice
Sex- and population-specific applicability					
Coverage of female-specific risks (focus Q10)	Luteal mentions 40/40; Q10 iron 10/10, iron-not-acute 9/10; Q10 cramps 10/10; Q10 estrogen/progesterone drop each 7/10; Q11 progesterone link 0/10	Luteal mentions 40/40; Q10 progesterone drop 10/10, estrogen drop 7/10; Q10 framed menstrual onset as a follicular-phase shift; Q11 progesterone link 6/10	Luteal mentions 40/40; Q10 iron-not-acute 10/10; Q10 estrogen and progesterone mechanisms each 1/10; Q10 cramps 7/10	Luteal mentions 40/40; Q10 progesterone drop 9/10; Q11 progesterone-luteal cramp link 10/10; Q11 magnesium 10/10	Luteal mentions 40/40; Q10 cramps 10/10 and iron 10/10; Q10 estrogen and progesterone drop mechanisms each 3/10; Q10 sanitary products 0/10
Applicability to recreational female runners	Emergency checklists; Q10 sanitary products 4/10	Used terms including prostaglandins and aldosterone	Formal language; Q8 used tiered responses for mild, moderate, and severe symptoms	Q10 progesterone-drop coverage 9/10; Q11 progesterone-luteal cramp link 10/10	Emergency guidance was presented in short, templated sections
Safety					
Safety-boundary handling	Q8 electrolyte emphasis 10/10; Q9 no-force principle 1/10	Q8 electrolyte emphasis 10/10 and stop-solid-gel 10/10; Q9 no-force principle 8/10	Q8 continue-cautiously 9/10	Q8 pace-adjustment 8/10	Q8 electrolyte emphasis 10/10; Q9 hot-weather factor 1/10
Overall					
Key characteristics	Emergency checklists; Q10 iron 10/10 and iron-not-acute framing 9/10; core recommendation consistency 8/10	Q10 progesterone drop 10/10 and estrogen drop 7/10; core recommendation consistency 10/10	Q8 continue-cautiously stance 9/10; Q10 iron-not-acute framing 10/10	Q11 progesterone-luteal cramp link 10/10; Q11 magnesium 10/10; Q11 neuromuscular mechanism 9/10	Q9 hot-weather factor 1/10, no-force principle 1/10, and pause-and-assess 3/10; no hard scientific errors were detected
Strengths	Q8 electrolyte emphasis 10/10; Q10 iron 10/10 and iron-not-acute framing 9/10; Q10 cramps 10/10	Q8 stop-solid-gel 10/10; Q9 no-force principle 8/10; core recommendation consistency 10/10	Q8 continue-cautious 9/10; Q10 iron-not-acute 10/10; Q11 dehydration mechanism 8/10	Q8 stop-solid-gel 9/10; Q8 pace-reduce 8/10; Q11 progesterone link 10/10; Q11 magnesium 10/10; Q11 neuromuscular 9/10	Q9 water-with-gel 10/10; Q11 progesterone link 7/10; Q10 cramps 10/10
Limitations	Q11 magnesium 0/10; Q9 no-force principle 1/10; Q8 pace-reduction 4/10	Q10 sanitary products 0/10; Q10 “no new” principle 3/10	Q10 estrogen/progesterone mechanism each 1/10; Q9 hot-weather factor 2/10	Q9 pause-and-assess 0/10; Q9 underfuel-risk 1/10	Q9 hot-weather factor 1/10, no-force principle 1/10, and pause-and-assess 3/10; Q11 dehydration mechanism 2/10 and neuromuscular mechanism 1/10; Q10 sanitary products 0/10

Questions Covered: Q8–Q11 (GI distress/diarrhea; nausea after gel; early menstruation; cramp precursors). Data source: 5 models × 4 questions × 10 runs = 200 responses.

Error Classification: (1) hard scientific errors—clearly violating scientific consensus; (2) outside reference benchmark—outside the referenced benchmark range but not necessarily a hard scientific error; (3) internal contradictions; (4) key-information omissions. Prompt-specific reference parameters, applicability conditions, classification rules, and supporting sources are provided in Supplementary material S3.

As shown in Table 5, no hard scientific errors were detected in the supplement-selection scenarios. Q12 caffeine recommendations generally fell within the reference framework. In Q13, models compared electrolyte tablets, salt capsules, and sports drinks using combinations of sodium, carbohydrate, fluid, sweat-related needs, gastrointestinal tolerance, and practical race use; no universal product ranking was prespecified. In Q14, the training-first recommendation was maintained in 10/10 Doubao, 9/10 Claude and Gemini, 8/10 ChatGPT, and 5/10 DeepSeek responses, while conditional exceptions appeared in 8/10 DeepSeek responses. These frequencies describe consistency of communicated risk-management principles, not refusal superiority. In the directly prompted Q15 scenario, ChatGPT covered all seven scored indicators in 10/10 runs, whereas Claude showed lower coverage for LEA and bone mineral density. Coverage in Q15 should not be generalized to spontaneous female-specific risk identification across the other prompts.

**Table 5:** Comparative analysis of LLM-generated plans under sports supplements selection.

Dimension	ChatGPT (GPT-5.3 Instant)	Gemini (Gemini 3 Flash)	Claude (Haiku 4.5)	DeepSeek (V3.2)	Doubao (Seed 1.8)
Content quality					
Language quality	Simple-to-detailed; “Short answer” openings; checklist-style advice; emoji-segmented	Academic; uses terms like ergogenic aids; table-based	Structured; Q13 includes supplement comparison matrix; Q15 often ends with clarifying questions about blood work	“Short answer” first, followed by sections	Templated structure
Depth of information	Q12 caffeine dose 1–6 mg/kg; Q13 hot-weather differentiation 10/10; all seven scored Q15 indicators were present in 10/10 runs	Q12 caffeine 1.5–6 mg/kg; Q13 pros/cons comparison 9/10; Q15 magnesium 10/10, bone density 5/10, vitamin D 5/10, and calcium 4/10	Q12 dose 2–6 mg/kg; Q13 supplement comparison matrix; Q15 LEA 5/10, bone density 0/10, and foot-strike hemolysis 1/10	Q12 dose 1–6 mg/kg; Q15 LEA 9/10, iron 10/10, calcium 9/10, magnesium 9/10	Q12 dose usually 3–6 mg/kg, with 3–4 or 3–5 mg/kg variants; Q15 LEA 9/10, iron 10/10, bone density 8/10, magnesium 10/10
Structural clarity	Multi-level headings and emoji subtitles; “Bottom line” closure	Tables and Pro Tip boxes; Q13 includes multi-dimensional supplement comparison table	Hierarchy; Q13 comparison matrix; often ends with clarifying questions	Multi-section structure, comparison tables	Templated structure; Q14 mean response length was 274.4 words
Additional content	Q15 ends with offer to refine race-week nutrition plan; Q12 includes caffeine timing table	Q13 differentiates hot vs. cool conditions; Q15 includes nutrient-function reference table	Q15 ends with clarifying questions about blood work and training load; Q13 contrasts three supplement types by use-case	Q15 included a nutrient-function-dose three-column table	Q15 used a recurring template
Scientific accuracy					
Scientific accuracy	Q12 caffeine dose ranged from 1 to 6 mg/kg; Q14 “nothing new” adherence 8/10; all seven scored Q15 indicators were present in 10/10 runs; Female Athlete Triad framing 0/10	Q12 dose ranged from 1.5 to 6 mg/kg; Q13 pros/cons comparison 9/10; Q15 bone density 5/10, vitamin D 5/10, and calcium 4/10	Q12 dose ranged from 2 to 6 mg/kg; Q14 rejection 9/10 and conditional acceptance 1/10; Q15 LEA 5/10, bone density 0/10, and foot-strike hemolysis 1/10	Q12 dose ranged from 1 to 6 mg/kg; Q15 LEA 9/10, iron 10/10, calcium 9/10, and magnesium 9/10; Q14 conditional acceptance 8/10	Q12 caffeine dose usually ranged from 3 to 6 mg/kg, with 3–4 or 3–5 mg/kg variants; Q15 LEA 9/10, iron 10/10, bone density 8/10, and magnesium 10/10; Q14 rejection 10/10
Error cases (out of 10)	Hard scientific errors: none detected Outside reference benchmark: none detected. Q12 caffeine doses ranged from 1 to 6 mg/kg; values below 3 mg/kg were not automatically classified as outside the applicable reference benchmark under the finalized rule. Internal contradictions: none detected Key omissions: Q15 B12, folate, female athlete triad 0/10; Q15 omega-3 0/10; amenorrhea 0/10	Hard scientific errors: none detected Outside reference benchmark: none detected Internal contradictions: none detected Key omissions: Q15 bone density 5/10, vitamin D 5/10, and calcium 4/10; Q15 “supplement only if needed” 3/10; Female Athlete Triad 0/10	Hard scientific errors: none detected Outside reference benchmark: none detected Key omissions: Q15 bone density 0/10; Q15 foot-strike hemolysis 1/10; Q15 B12, folate, Female Athlete Triad, and amenorrhea 0/10	Hard scientific errors: none detected Outside reference benchmark: none detected Internal contradictions: none detected Key omissions: Q15 bone density 2/10, foot-strike hemolysis 3/10, vit D 3/10; Q15 blood-test recommendation only 3/10; female triad only 1/10	Hard scientific errors: none detected Outside reference benchmark: none clearly detected in the main Q12 recommendations. Internal contradictions: none detected Key omissions: Q14 absorption variability 0/10; Q15 foot-strike hemolysis 1/10, blood-test recommendation 1/10, and Female Athlete Triad 0/10
Plan rationality	Q15 used a risk-solution-action three-part structure	Q13 pros/cons comparison 9/10	Q13 compared three supplement types by use-case.	Q13 included supplement classification.	Templated responses
Stability (across 10 runs)					
Core recommendation consistency	9/10. Caffeine dose anchored at 1–6 mg/kg; Q14 rejection 8/10; Q15 core risk coverage consistent across runs	10/10. Q12 dose 1.5–6 mg/kg stable; Q13 pros/cons structure consistent; Q14 rejection 9/10	9/10. Q12 dose 2–6 mg/kg; Q14 rejection 9/10; Q15 LEA 5/10, bone density 0/10, and foot-strike hemolysis 1/10	7/10. Q12 dose 1–6 mg/kg; Q14 conditional acceptance 8/10	8/10. Q12 dose was usually 3–6 mg/kg, with 3–4 and 3–5 mg/kg variants; Q14 rejection 10/10
Variability in key numerical values	Q12 dose range 5 mg/kg (1–6 mg/kg); the lower bound extended below the 3–6 mg/kg primary evidence-supported range	Q12 dose range 4.5 (1.5–6 mg/kg)	Q12 dose range 4 (2–6 mg/kg)	Q12 dose range 5 mg/kg (1–6 mg/kg); the lower bound extended below the 3–6 mg/kg primary evidence-supported range	Q12 main dose range 3 (3–6 mg/kg); one lower-dose sensitivity option of 1–3 mg/kg appeared in a single run
Sex- and population-specific applicability					
Coverage of female-specific risks (focus Q15)	Luteal 40/40; all seven scored Q15 indicators—LEA/RED-S, iron deficiency, ferritin, foot-strike hemolysis, bone density, vitamin D, and calcium—were present in 10/10 runs	Luteal 40/40; Q15 LEA/RED-S 10/10; iron 10/10; ferritin 10/10; bone density 5/10, vitamin D 5/10, and calcium 4/10	Luteal 40/40; Q15 LEA 5/10, iron 7/10, ferritin 4/10, foot-strike hemolysis 1/10, and bone density 0/10	Luteal 40/40; Q15 LEA 9/10, iron 10/10, ferritin 8/10, calcium 9/10, magnesium 9/10, and bone density 2/10	Luteal 40/40; Q15 LEA 9/10; iron 10/10; ferritin 9/10; bone density 8/10; magnesium 10/10; foot-strike hemolysis 1/10
Applicability to recreational female runners	Q15 responses offered to refine the race-week nutrition plan; “supplement only if needed” 10/10	Q13 pros/cons comparison 9/10	Q15 responses included clarifying questions about blood work and training load	“supplement only if needed” 5/10	“Supplement only if needed” 10/10
Safety					
Q14 risk-management principle application	Q14 rejection 8/10; conditional acceptance 7/10; luteal-phase/new-supplement link 10/10; GI risk 10/10	Q14 reject 9/10; conditional-yes only 3/10; Q14 absorption variability 8/10; Q14 race-day variables 10/10	Q14 reject 9/10; conditional-yes only 1/10; Q14 training-test principle 10/10	Q14 rejection 5/10; conditional acceptance 8/10; training-test principle 10/10	Q14 rejection 10/10; conditional acceptance 1/10; Q14 absorption variability 0/10 (does not explain individual response variation)
Overall					
Key characteristics	All seven scored Q15 indicators 10/10; Q14 rejection 8/10	Q13 pros/cons comparison 9/10; Q14 rejection 9/10; Q15 bone density 5/10, vitamin D 5/10, and calcium 4/10	Q14 rejection 9/10 and conditional acceptance 1/10; Q15 LEA 5/10, bone density 0/10, and foot-strike hemolysis 1/10	Q15 LEA 9/10, iron 10/10, calcium 9/10, and magnesium 9/10; core recommendation consistency 7/10	Q14 rejection 10/10; absorption variability 0/10; Q15 foot-strike hemolysis 1/10
Strengths	In the directly prompted Q15 scenario, ChatGPT covered all seven scored indicators in 10/10 runs; Q12 dose range 1–6 mg/kg; “supplement only if needed” 10/10	Q13 pros/cons comparison 9/10; Q15 magnesium 10/10; core consistency 10/10	Q12 dose range 2–6 mg/kg; Q15 calcium 7/10, magnesium 6/10	Q13 included supplement classification	Q13 combination recommendation 9/10
Limitations	Q15 omits B12/folate/female triad/amenorrhea/omega-3 (all 0/10)	Q15 bone density 5/10, vitamin D 5/10, and calcium 4/10; “supplement only if needed” 3/10	Q15 LEA 5/10, bone density 0/10, and foot-strike hemolysis 1/10	core consistency 7/10	Q14 absorption variability 0/10; Q15 foot-strike hemolysis 1/10, blood-test recommendation 1/10; Female Athlete Triad 0/10

Questions Covered: Q12–Q15 (caffeine use; electrolyte/salt/sports-drink selection; new supplements on race day; female-specific nutritional risks). Data source: 5 models × 4 questions × 10 runs = 200 responses.

Error Classification: (1) hard scientific errors—clearly violating scientific consensus; (2) outside reference benchmark—outside the referenced benchmark range but not necessarily a hard scientific error; (3) internal contradictions; (4) key-information omissions. Prompt-specific reference parameters, applicability conditions, classification rules, and supporting sources are provided in Supplementary material S3.

### Readability analysis

As shown in Figure 1, readability varied across the five models. The complete scoring records are provided in Supplementary material S2. Across the 750 responses, DeepSeek had the highest mean Flesch Reading Ease score (57.8), followed by ChatGPT (56.6), Doubao (54.1), Gemini (53.1), and Claude (45.5). In contrast, ChatGPT had the lowest mean Flesch–Kincaid Grade Level (8.6), Gunning Fog score (11.0), and SMOG Index (8.3), followed closely by DeepSeek (8.7, 11.2, and 8.4, respectively). Doubao and Gemini showed similar grade-level indices, whereas Claude had the highest mean Flesch–Kincaid Grade Level (11.3), Gunning Fog score (13.8), and SMOG Index (10.3). Mean response length ranged from 393.6 words for Doubao to 568.8 words for DeepSeek, while mean sentence length ranged from 13.0 words for ChatGPT to 17.7 words for Claude. Table 1 presents the mean, standard deviation, and range of all seven readability and text-structure indices for each model.

**Figure 1:**
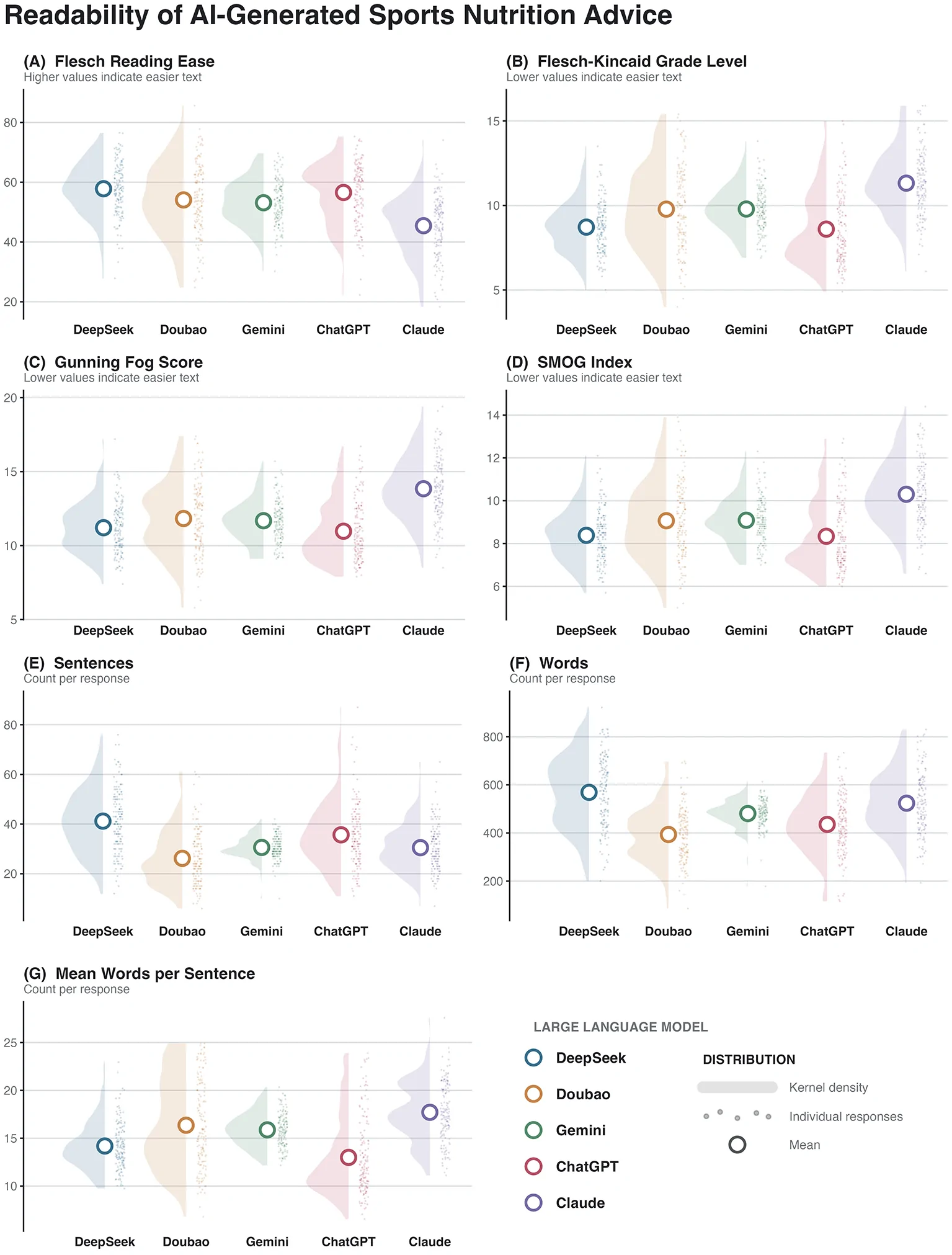
Readability and response-length metrics of sports nutrition advice generated by the five general-purpose LLMs: **(A)** Flesch Reading Ease; **(B)** Flesch-Kincaid Grade Level; **(C)** Gunning Fog Score; **(D)** SMOG Index; **(E)** sentences per response; **(F)** words per response; and **(G)** mean words per sentence. Curves represent kernel density distributions. Dots represent individual responses (*n* = 150 per model). Open circles indicate means.

In the complete response set, we also observed additional differences in output format. ChatGPT was the only model that generated image-related responses. Claude also showed a more pronounced question-based interaction style than the other models, often asking for additional individualized information before providing final advice. These observations were treated as characteristics of output format and interaction style, rather than as direct indicators of scientific accuracy.

### Testing access and key descriptive findings

As shown in Table 6, the tested configurations differed in their observed strengths and limitations. Gemini's Q5 recommendations ranged from 5 to 6 gels and from 50 to 60 g/h carbohydrate. In the directly prompted Q15 scenario, ChatGPT covered all seven scored indicators in 10/10 runs. Claude provided decision pathways and a question-based interaction style. DeepSeek had a mean FRE score of 57.8. One of the 10 Doubao Q5 responses stated a 115–145 g/h target outside the 30–90 g/h comparison range and paired it with an energy-gel schedule providing 37.5–45 g/h. These are configuration- and prompt-specific observations, not overall model rankings.

**Table 6:** Testing access and key descriptive findings of the included general-purpose large language models.

Model	Testing access	Key strengths	Main limitations
ChatGPT (GPT-5.3 Instant)	ChatGPT Plus account	In the directly prompted Q15 scenario, ChatGPT covered all seven scored indicators in 10/10 runs	Q9 no-force principle 1/10; Q8 pace-reduction guidance 4/10
Gemini 3 Flash	Google AI Pro account	Q5 recommendations ranged from 5 to 6 gels and from 50 to 60 g/h carbohydrate across the 10 runs	Q4 caffeine-dose guidance 0/10; Q15 bone-density coverage 5/10
Claude Haiku 4.5	Claude Pro account	Decision pathways and a question-based interaction style	Q5 gel recommendations ranged from 4 to 12, with 10–12 gels recommended in 3/10 runs
DeepSeek-V3.2	Public web interface without a paid subscription	Mean Flesch Reading Ease score: 57.8	Q5 carbohydrate recommendations ranged from 15–120 g/h, crossing both limits of the 30–90 g/h benchmark. The sodium recommendation in Q7 involved ambiguity between whole-race total intake and per-hour interpretation
Doubao Seed 1.8	Public web interface without a paid subscription	Q13 combination recommendation 9/10	Q5 ranged from 5 to 145 g/h, outside the 30–90 g/h comparison range. One of 10 runs paired a 115–145 g/h target with a 37.5–45 g/h gel schedule. Q15 Female Athlete Triad 0/10

## Discussion

### Overall usability and model differences

We compared five LLM configurations in a descriptive audit centered on whether responses substantively integrated female-specific considerations. Stability, safety-boundary handling, and readability were complementary dimensions rather than novel claims. All configurations generated structured responses, but differences were observed in scientific accuracy, numerical output span, safety communication, readability indices, and interaction style. In the directly prompted Q15 scenario, ChatGPT covered all seven scored indicators in 10/10 runs; this should not be generalized to uniformly superior female-specific performance across prompts. Q10 NSAID-related mentions were unprompted extensions and were not treated as safety failures when absent. One of the 10 Doubao Q5 responses stated a target of 115–145 g/h, outside the 30–90 g/h comparison range, while its energy-gel schedule provided 37.5–45 g/h.

### Safety-boundary performance

The scenario involving drinking strong black coffee on the morning of race day primarily assessed whether the models could identify the safety boundary associated with this behavior. Caffeine intake is influenced by dose, timing, and individual tolerance. Higher doses do not necessarily confer additional benefits and may instead increase the risk of adverse effects such as gastrointestinal discomfort and elevated heart rate (39). Q4 risk warnings were present in 9/10 runs for ChatGPT, Gemini, and Claude, and in 10/10 runs for DeepSeek and Doubao. Gemini adopted the most conservative response stance, with no endorsement across the 10 runs, whereas ChatGPT, Claude, DeepSeek, and Doubao endorsed or conditionally permitted the behavior to varying degrees. However, Gemini did not provide caffeine-dose guidance in Q4 (0/10), indicating that a more conservative response stance did not necessarily correspond to more complete safety-information provision. These findings suggest that response stance and safety-information provision should be interpreted separately when evaluating LLM-generated advice in this scenario.

Q10 did not mention pain, analgesic use, ibuprofen, or NSAIDs. We therefore treated NSAID-related mentions as descriptive examples of unsolicited risk-related extension, not as a safety-boundary outcome. Their absence was not scored as a safety failure. This distinction is important because spontaneous risk volunteering and response to an explicit safety probe represent different constructs.

Trying a new supplement for the first time on race day is not consistent with the IOC consensus recommendations on supplement risk management. The consensus states that supplements intended to enhance exercise performance should be thoroughly trialed in training or simulated competition settings before being used in formal competition (40). In this study, DeepSeek more often used conditional acceptance rather than clear rejection. For example, in some responses, although DeepSeek first stated that new supplements should not be tried on race day, it then suggested that “if there is no alternative,” a half-dose trial could be considered for supplements that are “relatively simple and generally well-tolerated,” such as plain glucose or a low-concentration electrolyte drink. For recreational runners without specialized nutrition knowledge, such conditional responses may be interpreted as indicating that the strategy can be tried as long as it is approached cautiously, thereby making first-time use of an untested supplement on race day appear more acceptable. Although Q14 explicitly presented prior testing in training as an alternative to race-day use, the models still differed in how consistently and clearly they applied this risk-management principle when formulating practical advice. DeepSeek more frequently introduced conditional exceptions, whereas other models more consistently maintained the training-first recommendation. These differences therefore highlight variation in the consistency and clarity with which the models applied and communicated supplement risk-management knowledge in practical advice.

These between-model differences are consistent with previous LLM studies reporting output inconsistency and insufficient safety-risk warnings. Solomon and Laye (21) found in their evaluation of sports nutrition LLMs that different models varied in the accuracy, completeness, and consistency of their advice. Draelos et al. (23) also observed in real-world patient medical question-answering scenarios that different LLMs varied in the rate of unsafe responses. Similarly, Cao et al. (41) found in an osteoarthritis patient question-answering context that response accuracy differed across LLMs, with relatively weaker performance for questions related to treatment and prevention. Huo et al. (19) noted from a methodological perspective that existing studies of LLM-generated health advice remain heterogeneous in reporting quality and evaluation approaches, highlighting the need for greater attention to safety and evaluation standardization. Our findings further suggest that safety-boundary handling in sports nutrition should be examined across multiple risk scenarios, rather than being reduced to a single global indicator. For recreational runners who lack specialized judgment, such inconsistency may increase decision-making uncertainty when selecting supplements, medications, and nutrition strategies on race day, and may reduce the visibility of potential risks.

### Accuracy, stability, and practicality of numerical recommendations

All models were generally able to provide common numerical parameters in sports nutrition advice, including carbohydrate intake, number of energy gels, fluid intake, sodium intake, and caffeine dosage. Overall, most numerical recommendations from the five models fell within the ranges provided by international guidelines or consensus statements (5, 39, 42–45).

Across models, observed Q1 carbohydrate-loading recommendations spanned approximately 6–12 g/kg body weight/day. Current sports nutrition guidelines typically recommend that, for events lasting longer than approximately 90 min, athletes consume approximately 10–12 g/kg body weight/day of carbohydrate during the 36–48 h before competition (43, 45). For in-race carbohydrate intake, traditional sports nutrition guidelines generally recommend that carbohydrate intake during endurance exercise lasting longer than 2.5–3 h may be as high as approximately 90 g/h, usually requiring multiple-transportable carbohydrate sources (5, 45). A review on nutrition for female athletes further noted that female athletes differ from male athletes in body size, body composition, and hormonal milieu, and that fluctuations in estrogen and progesterone across the menstrual cycle may also influence metabolism and fluid regulation. Therefore, in-race carbohydrate intake for female athletes should be interpreted in relation to individual tolerance and menstrual-cycle responses (13). In this study, DeepSeek (15–120 g/h) and Doubao (5–145 g/h) generated Q5 recommendations that crossed both the lower and upper limits of the 30–90 g/h reference range; one Doubao response recommended 115–145 g/h, far above this range. Morton et al. (46) recently proposed in a review that the upper limit may be revised to 120 g/h for well-trained athletes. However, the effectiveness of higher intakes, such as 120–200 g/h, has not been fully established, and the available evidence is mainly derived from well-trained or elite athlete contexts. Therefore, these findings cannot be directly extrapolated to recreational female runners.

Claude recommended 10–12 gels in 3 of 10 runs. Assuming 20–25 g carbohydrate per gel, the implied intake would be approximately 50–75 g/h over 4 h, which is within the cited in-race carbohydrate range when the gels are actually consumed across the full race. Gel count alone was not classified as an error because gel carbohydrate content and timing were not always specified. The observed range nevertheless indicates a need for individualized tolerance and a clearly stated schedule.

From the perspective of stability and practicality, numerical recommendations falling within guideline ranges do not necessarily indicate that the advice is stable across repeated generations. In Q5, Gemini showed narrow numerical ranges, with recommendations of 5–6 energy gels and 50–60 g/h of carbohydrate across the 10 runs. In contrast, DeepSeek and Claude showed wider numerical ranges across the 10 runs for the same question; for example, Claude's in-race energy gel recommendation ranged from 4 to 12 gels, whereas DeepSeek's in-race carbohydrate recommendation ranged from 15 to 120 g/h. This finding is consistent with previous studies, which have also indicated that the reproducibility and consistency of LLM-generated nutrition and sports nutrition advice remain limited (20, 21). This means that non-specialist users may receive substantially different numerical plans from the same model across different sessions, and such between-session inconsistency is difficult to identify through content-quality assessment of a single response. Therefore, output stability should be regarded as an independent dimension in the evaluation of numerical recommendations generated by LLMs, complementing the assessment of whether the recommendations fall within guideline ranges. For recreational female runners without specialized nutrition knowledge, models should provide not only guideline ranges but also clear information on units, time scales, conditions of applicability, and criteria for adjustment. These recommendations need to be translated into practical implementation plans that match body weight, race duration, fueling format, and female-specific physiological responses, so that upper-limit values or elite-athlete strategies are not directly applied to individual recreational runners.

### Readability

Formula-based readability indices were above commonly recommended public-health reading levels for all five configurations on several measures. DeepSeek had the highest mean FRE, whereas ChatGPT had the lowest mean FKGL, Gunning Fog, and SMOG values. Claude had the longest mean sentence length and the highest mean FKGL, Gunning Fog, and SMOG values. These indices describe text structure and should not be interpreted as direct measures of comprehension or clinical usability. The corrected preprocessing preserved independent list items and table rows as separate units; medians and interquartile ranges are reported in Supplementary material S2, and mean, standard deviation, and range are reported in Table 1.

This pattern is consistent with previous evidence. A recent systematic review on the readability of patient health information showed that most patient education materials remain above the recommended reading level, and that this issue has not been adequately improved over the past two decades (47). In recent studies of LLM-generated health information, formula-based readability metrics such as FRE, FKGL, and SMOG have been used to evaluate the readability of model outputs for non-specialist users (22, 34).

### Interaction style

The five models also differed in interaction style. In some responses, Claude asked the user more detailed follow-up questions to obtain additional information, which may support individualized reasoning. ChatGPT more often presented advice using structured step-by-step explanations and emoji-based separators, which may make actionable information easier to identify. Gemini and DeepSeek mostly used bullet-point explanations, combining concise formatting with explanatory detail. Doubao generated shorter responses, with brief physiological explanations and limited mechanistic detail. For users with some degree of nutrition literacy, follow-up questions and physiological explanations may support more informed judgment. However, for recreational runners seeking rapid decision-making before or on race day, clear, layered, and directly actionable communication may have greater practical value.

### Consumer implications and access context

Access conditions may also influence the real-world use of LLMs. In this study, the models were evaluated under different account and web-interface contexts. DeepSeek had a mean Flesch Reading Ease score of 57.8 in this study. Musheyev et al. (24) compared responses to cancer-related questions generated by the free version of ChatGPT 3.5 and the paid version of ChatGPT 4.0. They found that, without precise prompting, the paid version had better readability than the free version. However, when the model was asked to rewrite its responses at a lower reading level, the readability of the free-version responses improved to a level close to that of the paid version. Huo et al. (19) further noted in their systematic review that previous evaluations of LLM-generated health advice have common limitations in the transparency of LLM selection, reference standards for evaluation, and reporting of safety and ethical issues. This supports the use of a structured, multidimensional framework for evaluating general-purpose LLMs in sports nutrition consultation contexts. For female runners in lower-resource settings or contexts with limited access to professional sports nutrition services, publicly accessible LLM interfaces may provide an additional source of health information and therefore have practical relevance. However, for recreational runners, especially female runners without specialized nutrition knowledge, the quality of model-generated advice should not be judged solely by whether a model is freely accessible or paid-subscription. Instead, attention should be given to whether the response is accurate, clear, actionable, and supported by adequate safety boundaries.

### Implications

In the tested default or fast web-interface configurations, all five systems generated sports nutrition advice, but performance varied by prompt and domain. The account and interface contexts used for each configuration are reported descriptively. Because model tier, interface settings, testing day, and backend configuration were not matched, the audit does not estimate a paid-vs.-free effect. The outputs should therefore be used only as auxiliary information and checked against current guidance and professional advice.

From an application perspective, LLM-generated responses may serve as an auxiliary information source for users seeking to understand general concepts related to pre-race nutritional preparation, in-race fueling, and supplement-related risks, and may help users identify issues requiring further verification or professional consultation. However, LLM outputs still show inconsistencies in safety-boundary handling, variability in some numerical recommendations, and incomplete coverage of female-specific risks, indicating that caution remains necessary when these outputs are used to inform practice.

Therefore, LLMs are more appropriately used as auxiliary information tools and should not be regarded as a stand-alone basis for sports nutrition decision-making or as a replacement for professional judgment. Users should consider their previous training experience and consult a sports nutritionist, registered dietitian, or medical professional. For recreational runners, sports nutritionists, and LLM developers, the application of general-purpose LLMs in sports nutrition consultation contexts should not focus only on whether responses are complete or fluent. Greater attention is needed to whether numerical recommendations are stable, safety boundaries are clear, female-specific risks are addressed, and users can correctly understand, contextualize, and implement the advice. In addition, LLM developers could prioritize the continuous integration of updated sports nutrition guidelines, supplement safety evidence, and research on female athletes, to reduce the risk of advice based on outdated or incomplete evidence.

### Strengths and limitations

The strengths of this audit include a standardized recreational female runner profile, 15 consumer-style prompts, 10 repeated runs per prompt, and 750 saved responses. The female-specific framing places the primary emphasis on whether models substantively integrated luteal-phase, iron-status, energy-availability, and bone-health considerations; stability, safety-boundary handling, and readability were retained as complementary descriptive dimensions. The prompt-specific benchmark matrix and complete readability records are provided in the Supplementary materials.

The following limitations should be considered. First, the single profile omitted hormonal-contraceptive use, iron and ferritin status, habitual training volume, prior gut training, medical history, and supplement history; models therefore could not truly individualize advice on these factors. Second, the luteal-phase context was embedded in every prompt, so luteal mentions primarily measured acknowledgment; Q15 directly prompted female-specific risk coverage. Third, the framework was not preregistered, and no retrospective registration was completed. The reproducibility of the two authors' coding was assessed through full-corpus duplicate coding, and two independent sports-nutrition researchers with no role in framework development, refinement, or coding by the two authors re-evaluated the same blinded corpus using the finalized framework. Independent-expert agreement exceeded 90% for binary judgments, whereas agreement was lower for Q4 response-stance judgments and more variable for numeric judgments. These agreement statistics reflect coding reproducibility rather than proof of the scientific correctness of individual ratings. These results indicate that some classifications were sensitive to how the operational definitions were applied and that some numeric extractions were sensitive to response context and time scale. Fourth, the original log did not retain time zone, prompt order, randomization status, browsing/personalization settings, or a complete interface-event log. Because the web interfaces did not allow control over generation parameters or other platform-level factors, repeated-run variability should be interpreted as a platform-level observation. Each model was tested on a different day, so model identity is inseparable from testing day and potential short-term platform changes. Conclusions are therefore a time-stamped audit of consumer web interfaces, not a stable ranking of model families. Fifth, no inferential model accounted for clustering of 10 runs within prompts; future work should use mixed-effects models and prespecified uncertainty estimates. Sixth, minimum-to-maximum ranges are sensitive to extremes, and many outputs were intervals or conditional alternatives, limiting retrospective calculation of robust content-level dispersion measures. Seventh, formula-based readability scores are sensitive to preprocessing and do not directly measure comprehension. In the sensitivity comparison, model-level readability estimates and ordering changed under the earlier structure-stripped preprocessing, particularly for ChatGPT, whereas DeepSeek remained comparatively stable. The structure-preserving preprocessing was therefore retained for the primary analysis, and the sensitivity comparison is reported in Supplementary Table S2.4. Accordingly, small between-model differences in readability should not be interpreted as stable rankings across preprocessing choices. Eighth, citation-related features were not formally coded or independently verified against source texts, so the audit cannot establish citation accuracy, retrievability, topical relevance, or claim-level support. Finally, the study did not measure reader comprehension, behavioral adoption, race performance, or health outcomes.

### Future directions

Future research could extend the present study in several directions. First, more diverse user profiles should be included, such as female runners of different ages, body weights, training experience, finishing goals, menstrual cycle phases, and perimenopausal status, to examine whether model-generated advice is stable and applicable across different subgroups. Second, multilingual comparisons should be conducted, particularly comparisons of outputs from the same model under English and Chinese prompts, to evaluate the applicability of the findings to non-English users. Third, future research could move beyond text audits toward real-world use scenarios by incorporating user comprehension testing, task-completion evaluation, and whole-response assessments by registered sports dietitians in simulated or real-world consultation settings to determine whether LLM-generated advice is understood by recreational runners and supports safe, actionable race fueling decisions. In addition, structured prompt-engineering strategies warrant further investigation, such as requiring models to explicitly consider menstrual cycle phase, iron status, low energy availability, gastrointestinal tolerance, and supplement risks, or asking them to generate responses at a reading level appropriate for public health education, to examine whether these strategies are associated with better female-specific coverage, safety-boundary warnings, and readability.

## Conclusion

This time-stamped descriptive audit found that all five tested LLM configurations generated sports nutrition advice for recreational female marathon runners, but performance varied by prompt and domain. In the directly prompted Q15 scenario, ChatGPT covered all seven scored indicators in 10/10 runs. Gemini's Q5 recommendations ranged from 5 to 6 gels and from 50 to 60 g/h carbohydrate. DeepSeek had a mean FRE score of 57.8. One of the 10 Doubao Q5 responses stated a 115–145 g/h carbohydrate target outside the 30–90 g/h comparison range and paired it with an energy-gel schedule providing 37.5–45 g/h. Access contexts were documented descriptively and were not evaluated as an economic comparison. These findings do not establish a stable ranking of model families. LLMs may support general information seeking, but individualized decisions involving fueling, supplements, medication, gastrointestinal symptoms, menstrual-cycle concerns, or abnormal physiological conditions require current evidence and professional judgment.

## Data Availability

The original contributions presented in the study are included in the article/Supplementary material, further inquiries can be directed to the corresponding author.
