# Supplementary material for: Evaluating sports nutrition advice provided by general-purpose large language models for recreational female marathon runners: a multidimensional descriptive audit
Source: Front Nutr. 2026 Sep 21;13:1902377. doi: 10.3389/fnut.2026.1902377 (PMC13635112; doi:10.3389/fnut.2026.1902377)
Supplement: Supplementary file 2 [file Table_2.docx]

**Supplementary Material 2**

**Complete readability scoring records for the 750 responses generated by five general-purpose large language models**

This supplement provides the complete readability scoring records for 750 responses (5 models × 15 prompts × 10 runs) generated by ChatGPT, Gemini, Claude, DeepSeek, and Doubao. The WebFX output was recorded for seven indices: Flesch Reading Ease (FRE), Flesch–Kincaid Grade Level (FKGL), Gunning Fog Score, SMOG Index, sentence count, word count, and mean words per sentence. The records are descriptive and are not inferential estimates.

## **Readability Preprocessing**

## Higher FRE scores and lower FKGL, Gunning Fog, and SMOG values indicate greater readability. Before WebFX analysis, formatting markers were normalized uniformly across all five configurations. Substantive wording, numerical values, units, and existing punctuation were retained. Independent bullet-list items and table rows remained separate text units; a period was added only when such a unit lacked recognizable terminal punctuation. Continuous prose was not arbitrarily split. The complete records permit inspection of the preprocessing-sensitive results.

The preprocessing procedure was limited to formatting normalization and did not alter the substantive wording, numerical values, units, or recommendations in the model responses. Emphasis markers and heading markers were removed, while existing sentence-ending punctuation was retained. Independent bullet-list items and table rows were preserved as separate text units. When an independent list item or table row lacked terminal punctuation recognizable by WebFX, a period was added at the end of that unit. Continuous prose was not arbitrarily split into shorter sentences.

## **Table S2.1. Median and interquartile ranges of readability and text-structure indices by model**

## *Values are median (Q1–Q3) across 150 responses per model (15 prompts × 10 independent runs). Q1 and Q3 represent the 25th and 75th percentiles. Means, standard deviations, and ranges are reported in the main-text readability table; prompt-stratified means are reported in Table S2.2.*

| **Model** | **FRE** | **FKGL** | **Gunning Fog** | **SMOG** | **Sentences** | **Words** | **Words/sentence** |
| --- | --- | --- | --- | --- | --- | --- | --- |
| **ChatGPT** | **59.15 (49.65–63.98)** | **8.00 (7.00–10.10)** | **10.50 (9.50–12.40)** | **7.80 (7.20–9.40)** | **34.00 (27.00–42.75)** | **432.50 (360.50–509.00)** | **11.79 (10.10–14.82)** |
| **Gemini** | **53.15 (48.13–58.38)** | **9.70 (8.93–10.60)** | **11.60 (10.80–12.48)** | **9.00 (8.43–9.78)** | **30.00 (28.00–34.00)** | **484.50 (454.00–513.75)** | **15.83 (14.51–16.99)** |
| **Claude** | **46.50 (39.30–51.98)** | **11.20 (10.13–12.50)** | **13.80 (12.53–15.20)** | **10.20 (9.20–11.30)** | **29.00 (24.00–36.00)** | **518.50 (433.50–610.25)** | **17.49 (15.76–20.12)** |
| **DeepSeek** | **57.90 (52.08–63.78)** | **8.70 (7.60–9.60)** | **11.10 (10.00–12.30)** | **8.40 (7.50–9.10)** | **41.00 (32.00–50.00)** | **576.00 (453.25–678.50)** | **13.97 (12.66–15.62)** |
| **Doubao** | **53.85 (45.88–62.60)** | **9.65 (8.00–11.58)** | **11.80 (10.13–13.50)** | **8.85 (7.73–10.20)** | **24.00 (17.25–33.00)** | **396.00 (317.25–467.25)** | **15.54 (12.67–20.21)** |

*Abbreviations: FRE, Flesch Reading Ease; FKGL, Flesch–Kincaid Grade Level; SMOG, Simple Measure of Gobbledygook. Higher FRE scores and lower FKGL, Gunning Fog, and SMOG values indicate greater readability.*

## **Supplementary Figure S2.1. Median and interquartile ranges of readability and text-structure indices by model.**

## **Table S2.2. Mean readability indices by model and prompt**

*Values are means across 10 independent runs per cell. Full prompt text is provided in Supplementary Material 1.*

| **Model** | **PromptID** | **FRE** | **FKGL** | **Gunning Fog** | **SMOG** | **Sentences** | **Words** | **Words / sentence** |
| --- | --- | --- | --- | --- | --- | --- | --- | --- |
| **ChatGPT** | **Q1** | 63.90 | 6.86 | 9.35 | 7.11 | 44.30 | 434.90 | 10.10 |
| **ChatGPT** | **Q2** | 64.75 | 6.75 | 9.33 | 7.10 | 24.50 | 254.90 | 10.13 |
| **ChatGPT** | **Q3** | 42.32 | 10.43 | 12.30 | 9.69 | 46.30 | 553.80 | 12.33 |
| **ChatGPT** | **Q4** | 65.91 | 7.16 | 9.46 | 7.10 | 34.10 | 421.60 | 12.41 |
| **ChatGPT** | **Q5** | 70.01 | 6.29 | 9.23 | 6.97 | 24.60 | 272.20 | 11.24 |
| **ChatGPT** | **Q6** | 60.39 | 7.60 | 10.69 | 7.92 | 32.10 | 355.30 | 11.19 |
| **ChatGPT** | **Q7** | 60.51 | 7.18 | 9.88 | 7.32 | 39.90 | 374.80 | 9.53 |
| **ChatGPT** | **Q8** | 51.74 | 8.87 | 11.14 | 8.29 | 43.00 | 466.10 | 11.32 |
| **ChatGPT** | **Q9** | 62.39 | 8.13 | 9.93 | 7.44 | 30.20 | 416.10 | 14.40 |
| **ChatGPT** | **Q10** | 51.71 | 10.00 | 12.59 | 9.51 | 32.10 | 473.90 | 15.99 |
| **ChatGPT** | **Q11** | 51.03 | 10.14 | 12.00 | 9.15 | 34.80 | 537.10 | 16.04 |
| **ChatGPT** | **Q12** | 60.40 | 8.62 | 11.07 | 8.37 | 35.70 | 499.50 | 15.19 |
| **ChatGPT** | **Q13** | 56.57 | 8.85 | 11.22 | 8.68 | 53.90 | 637.40 | 14.03 |
| **ChatGPT** | **Q14** | 48.53 | 10.55 | 13.30 | 9.96 | 22.70 | 333.90 | 16.33 |
| **ChatGPT** | **Q15** | 38.42 | 11.55 | 13.06 | 10.45 | 37.00 | 501.80 | 14.71 |
| **Gemini** | **Q1** | 63.96 | 8.09 | 10.14 | 7.81 | 33.50 | 501.70 | 15.14 |
| **Gemini** | **Q2** | 63.08 | 8.15 | 9.90 | 7.69 | 30.20 | 440.90 | 14.88 |
| **Gemini** | **Q3** | 49.05 | 9.80 | 11.66 | 8.97 | 36.10 | 489.20 | 13.62 |
| **Gemini** | **Q4** | 57.02 | 9.18 | 10.92 | 8.36 | 30.70 | 478.60 | 15.62 |
| **Gemini** | **Q5** | 59.51 | 8.81 | 10.87 | 8.53 | 26.90 | 415.30 | 15.48 |
| **Gemini** | **Q6** | 54.92 | 9.75 | 11.87 | 9.18 | 27.70 | 460.10 | 16.71 |
| **Gemini** | **Q7** | 48.47 | 10.47 | 12.18 | 9.58 | 28.50 | 454.20 | 16.00 |
| **Gemini** | **Q8** | 51.23 | 10.06 | 12.07 | 9.44 | 32.00 | 505.50 | 15.88 |
| **Gemini** | **Q9** | 56.59 | 9.00 | 10.78 | 8.40 | 35.90 | 521.90 | 14.62 |
| **Gemini** | **Q10** | 42.84 | 11.69 | 13.85 | 10.82 | 29.60 | 520.00 | 17.77 |
| **Gemini** | **Q11** | 50.05 | 10.42 | 11.65 | 9.23 | 30.10 | 501.30 | 16.70 |
| **Gemini** | **Q12** | 54.06 | 9.81 | 11.84 | 9.00 | 28.40 | 463.20 | 16.44 |
| **Gemini** | **Q13** | 54.20 | 9.55 | 11.07 | 8.79 | 31.30 | 480.90 | 15.46 |
| **Gemini** | **Q14** | 51.07 | 10.31 | 13.05 | 9.94 | 25.70 | 431.80 | 16.84 |
| **Gemini** | **Q15** | 41.03 | 11.74 | 13.37 | 10.56 | 32.40 | 545.40 | 16.92 |
| **Claude** | **Q1** | 49.19 | 11.27 | 13.51 | 10.11 | 32.90 | 634.60 | 19.68 |
| **Claude** | **Q2** | 56.39 | 9.81 | 11.86 | 8.82 | 34.40 | 591.00 | 17.75 |
| **Claude** | **Q3** | 33.76 | 12.94 | 15.63 | 11.67 | 33.70 | 585.40 | 17.68 |
| **Claude** | **Q4** | 55.56 | 9.57 | 11.72 | 8.55 | 26.80 | 418.80 | 16.32 |
| **Claude** | **Q5** | 50.48 | 10.52 | 12.78 | 9.59 | 25.50 | 428.10 | 17.28 |
| **Claude** | **Q6** | 48.59 | 11.06 | 14.11 | 10.36 | 22.60 | 405.20 | 18.38 |
| **Claude** | **Q7** | 41.26 | 12.33 | 14.87 | 11.24 | 25.00 | 463.20 | 19.44 |
| **Claude** | **Q8** | 46.22 | 10.93 | 13.43 | 10.03 | 41.00 | 676.30 | 16.58 |
| **Claude** | **Q9** | 53.26 | 9.77 | 12.54 | 9.26 | 32.20 | 495.40 | 15.86 |
| **Claude** | **Q10** | 34.78 | 12.88 | 15.90 | 11.73 | 30.20 | 523.50 | 17.96 |
| **Claude** | **Q11** | 44.54 | 11.11 | 13.63 | 10.08 | 40.80 | 634.40 | 16.32 |
| **Claude** | **Q12** | 49.48 | 10.78 | 12.62 | 9.39 | 30.60 | 532.40 | 17.80 |
| **Claude** | **Q13** | 47.30 | 10.91 | 13.59 | 10.20 | 32.10 | 541.90 | 17.15 |
| **Claude** | **Q14** | 44.19 | 11.50 | 14.87 | 11.04 | 20.10 | 351.80 | 17.78 |
| **Claude** | **Q15** | 26.89 | 14.39 | 16.60 | 12.43 | 29.80 | 574.10 | 19.65 |
| **DeepSeek** | **Q1** | 62.45 | 8.70 | 11.18 | 8.26 | 38.30 | 592.00 | 16.68 |
| **DeepSeek** | **Q2** | 65.18 | 7.87 | 10.01 | 7.45 | 32.20 | 480.20 | 14.90 |
| **DeepSeek** | **Q3** | 52.16 | 9.71 | 11.90 | 9.26 | 46.60 | 701.60 | 15.06 |
| **DeepSeek** | **Q4** | 67.85 | 6.90 | 9.13 | 6.81 | 43.60 | 540.70 | 12.47 |
| **DeepSeek** | **Q5** | 68.62 | 7.03 | 9.78 | 7.26 | 40.40 | 533.20 | 13.46 |
| **DeepSeek** | **Q6** | 58.71 | 8.79 | 11.65 | 8.60 | 33.00 | 487.50 | 15.00 |
| **DeepSeek** | **Q7** | 61.47 | 7.92 | 10.47 | 8.07 | 50.60 | 654.50 | 13.00 |
| **DeepSeek** | **Q8** | 47.87 | 10.13 | 13.06 | 9.76 | 49.30 | 680.10 | 14.31 |
| **DeepSeek** | **Q9** | 61.09 | 8.17 | 10.34 | 7.70 | 33.40 | 456.50 | 13.80 |
| **DeepSeek** | **Q10** | 51.08 | 9.69 | 12.62 | 9.28 | 38.90 | 543.70 | 14.30 |
| **DeepSeek** | **Q11** | 54.30 | 8.76 | 11.14 | 8.29 | 59.90 | 738.60 | 12.39 |
| **DeepSeek** | **Q12** | 63.05 | 7.99 | 10.16 | 7.58 | 38.00 | 527.30 | 14.20 |
| **DeepSeek** | **Q13** | 57.88 | 8.81 | 10.92 | 8.44 | 48.40 | 687.10 | 14.60 |
| **DeepSeek** | **Q14** | 53.39 | 9.44 | 12.24 | 9.00 | 29.80 | 423.30 | 14.55 |
| **DeepSeek** | **Q15** | 42.28 | 10.86 | 13.53 | 9.98 | 35.60 | 486.10 | 14.08 |
| **Doubao** | **Q1** | 68.63 | 6.79 | 8.93 | 6.88 | 30.60 | 365.00 | 12.55 |
| **Doubao** | **Q2** | 64.69 | 7.47 | 9.33 | 7.25 | 29.00 | 316.00 | 12.95 |
| **Doubao** | **Q3** | 43.74 | 11.39 | 13.19 | 10.49 | 20.20 | 327.20 | 17.06 |
| **Doubao** | **Q4** | 60.97 | 9.10 | 11.28 | 8.26 | 27.40 | 460.70 | 17.43 |
| **Doubao** | **Q5** | 72.57 | 7.41 | 9.85 | 7.22 | 16.50 | 268.50 | 17.21 |
| **Doubao** | **Q6** | 57.63 | 9.44 | 12.07 | 9.15 | 21.40 | 344.70 | 16.94 |
| **Doubao** | **Q7** | 53.45 | 9.67 | 11.72 | 9.05 | 27.20 | 395.20 | 15.55 |
| **Doubao** | **Q8** | 42.40 | 11.57 | 13.20 | 10.28 | 27.40 | 434.60 | 17.01 |
| **Doubao** | **Q9** | 60.16 | 9.30 | 11.63 | 8.55 | 19.10 | 327.40 | 17.82 |
| **Doubao** | **Q10** | 45.97 | 10.45 | 12.80 | 9.73 | 33.00 | 455.30 | 14.52 |
| **Doubao** | **Q11** | 49.03 | 11.01 | 12.33 | 9.69 | 30.10 | 512.00 | 18.44 |
| **Doubao** | **Q12** | 53.74 | 10.41 | 12.59 | 9.39 | 22.60 | 391.60 | 18.72 |
| **Doubao** | **Q13** | 56.83 | 8.41 | 10.42 | 8.27 | 42.90 | 527.90 | 12.45 |
| **Doubao** | **Q14** | 47.52 | 11.09 | 13.42 | 10.05 | 16.70 | 274.40 | 17.92 |
| **Doubao** | **Q15** | 33.87 | 13.21 | 14.68 | 11.70 | 28.90 | 503.20 | 18.79 |

*Abbreviations as in Table S2.1.*

## **Table S2.3. Complete record of all 750 responses**

*One row per response. Records are grouped by model and sorted by PromptID and Run.*

### **S2.3.1 ChatGPT (n = 150)**

| **PromptID** | **Run** | **FRE** | **FKGL** | **Gunning Fog** | **SMOG** | **Sentences** | **Words** | **Words / sentence** |
| --- | --- | --- | --- | --- | --- | --- | --- | --- |
| **Q1** | 1 | 66.4 | 5.9 | 8.7 | 6.4 | 51 | 389 | 7.63 |
| **Q1** | 2 | 64.1 | 6.9 | 9.4 | 7.2 | 42 | 435 | 10.36 |
| **Q1** | 3 | 61.8 | 8.1 | 10.5 | 8.2 | 31 | 426 | 13.74 |
| **Q1** | 4 | 65.8 | 6.1 | 8.2 | 6.4 | 60 | 493 | 8.22 |
| **Q1** | 5 | 63.9 | 6.8 | 9.3 | 7.2 | 41 | 407 | 9.93 |
| **Q1** | 6 | 63.5 | 6.4 | 8.9 | 6.6 | 53 | 435 | 8.21 |
| **Q1** | 7 | 68.1 | 6.4 | 8.8 | 6.7 | 44 | 463 | 10.52 |
| **Q1** | 8 | 62.9 | 6.9 | 9.8 | 7.1 | 42 | 400 | 9.52 |
| **Q1** | 9 | 60.5 | 7.6 | 9.8 | 7.8 | 40 | 451 | 11.28 |
| **Q1** | 10 | 62 | 7.5 | 10.1 | 7.5 | 39 | 450 | 11.54 |
| **Q2** | 1 | 61.3 | 7.2 | 10.6 | 7.6 | 14 | 138 | 9.86 |
| **Q2** | 2 | 74.4 | 4.9 | 8.5 | 6.2 | 17 | 139 | 8.18 |
| **Q2** | 3 | 67.6 | 6.2 | 9.7 | 7 | 13 | 122 | 9.38 |
| **Q2** | 4 | 67.8 | 5.7 | 8.5 | 6.3 | 19 | 147 | 7.74 |
| **Q2** | 5 | 50.3 | 8.9 | 12.5 | 9 | 11 | 116 | 10.55 |
| **Q2** | 6 | 68.3 | 6.2 | 8 | 6.4 | 36 | 354 | 9.83 |
| **Q2** | 7 | 66 | 7 | 8.3 | 6.9 | 34 | 407 | 11.97 |
| **Q2** | 8 | 62.5 | 7.7 | 9.1 | 7.3 | 29 | 369 | 12.72 |
| **Q2** | 9 | 60.3 | 7.5 | 9.2 | 7.4 | 34 | 358 | 10.53 |
| **Q2** | 10 | 69 | 6.2 | 8.9 | 6.9 | 38 | 399 | 10.5 |
| **Q3** | 1 | 40.3 | 10.2 | 11.5 | 9.2 | 52 | 538 | 10.35 |
| **Q3** | 2 | 42.1 | 10.8 | 12.5 | 10.2 | 41 | 562 | 13.71 |
| **Q3** | 3 | 44 | 9.6 | 11.1 | 8.8 | 58 | 567 | 9.78 |
| **Q3** | 4 | 45.1 | 9.6 | 11.9 | 9.2 | 54 | 578 | 10.7 |
| **Q3** | 5 | 41.8 | 10.6 | 12.4 | 9.8 | 48 | 604 | 12.58 |
| **Q3** | 6 | 42.9 | 10.2 | 12 | 9.5 | 50 | 595 | 11.9 |
| **Q3** | 7 | 40.7 | 10.5 | 12.9 | 9.7 | 42 | 496 | 11.81 |
| **Q3** | 8 | 43.7 | 10 | 11.9 | 9.5 | 45 | 510 | 11.33 |
| **Q3** | 9 | 38.7 | 12.4 | 14.2 | 11.1 | 27 | 488 | 18.07 |
| **Q3** | 10 | 43.9 | 10.4 | 12.6 | 9.9 | 46 | 600 | 13.04 |
| **Q4** | 1 | 69.2 | 6.7 | 8.9 | 6.6 | 26 | 319 | 12.27 |
| **Q4** | 2 | 64.8 | 7.1 | 9.5 | 7.3 | 41 | 481 | 11.73 |
| **Q4** | 3 | 66.1 | 7.3 | 9.9 | 7.4 | 31 | 410 | 13.23 |
| **Q4** | 4 | 66 | 7.2 | 9.3 | 7 | 37 | 470 | 12.7 |
| **Q4** | 5 | 71.1 | 6 | 8.4 | 6.4 | 37 | 397 | 10.73 |
| **Q4** | 6 | 66.3 | 7 | 9.5 | 7 | 32 | 380 | 11.88 |
| **Q4** | 7 | 62.3 | 8.4 | 10.6 | 7.8 | 33 | 505 | 15.3 |
| **Q4** | 8 | 63.9 | 7.6 | 10.1 | 7.6 | 32 | 414 | 12.94 |
| **Q4** | 9 | 64.7 | 7.1 | 9.5 | 7.1 | 36 | 410 | 11.39 |
| **Q4** | 10 | 64.7 | 7.2 | 8.9 | 6.8 | 36 | 430 | 11.94 |
| **Q5** | 1 | 74 | 5.7 | 8.7 | 6.7 | 24 | 272 | 11.33 |
| **Q5** | 2 | 75.3 | 5.4 | 8.5 | 6.4 | 31 | 330 | 10.65 |
| **Q5** | 3 | 73.8 | 5.4 | 8.5 | 6.3 | 23 | 221 | 9.61 |
| **Q5** | 4 | 60.6 | 8.5 | 11 | 8.5 | 18 | 267 | 14.83 |
| **Q5** | 5 | 64 | 6.7 | 9.2 | 7 | 26 | 245 | 9.42 |
| **Q5** | 6 | 69.5 | 6.1 | 8.8 | 6.5 | 26 | 263 | 10.12 |
| **Q5** | 7 | 73.7 | 5.6 | 8.8 | 6.6 | 28 | 295 | 10.54 |
| **Q5** | 8 | 72.3 | 6.8 | 10.1 | 7.6 | 21 | 305 | 14.52 |
| **Q5** | 9 | 69.2 | 6.1 | 9.6 | 7.2 | 25 | 256 | 10.24 |
| **Q5** | 10 | 67.7 | 6.6 | 9.1 | 6.9 | 24 | 268 | 11.17 |
| **Q6** | 1 | 57.7 | 8.8 | 12.2 | 9 | 22 | 317 | 14.41 |
| **Q6** | 2 | 60.9 | 7.7 | 11 | 8.4 | 29 | 348 | 12 |
| **Q6** | 3 | 60.7 | 7.4 | 10.2 | 7.6 | 34 | 360 | 10.59 |
| **Q6** | 4 | 59 | 7.7 | 11.3 | 8.3 | 33 | 358 | 10.85 |
| **Q6** | 5 | 57.4 | 8.1 | 11.5 | 8.4 | 35 | 407 | 11.63 |
| **Q6** | 6 | 64.2 | 6.8 | 9.6 | 7.2 | 32 | 320 | 10 |
| **Q6** | 7 | 58.5 | 7.6 | 10.5 | 7.7 | 37 | 379 | 10.24 |
| **Q6** | 8 | 60.2 | 7.8 | 11.2 | 8.1 | 34 | 396 | 11.65 |
| **Q6** | 9 | 61.8 | 7.1 | 9.4 | 7.1 | 33 | 332 | 10.06 |
| **Q6** | 10 | 63.5 | 7 | 10 | 7.4 | 32 | 336 | 10.5 |
| **Q7** | 1 | 64.5 | 6.5 | 9.1 | 7 | 43 | 395 | 9.19 |
| **Q7** | 2 | 60.9 | 6.8 | 10.4 | 7.3 | 44 | 368 | 8.36 |
| **Q7** | 3 | 63.3 | 6.1 | 8.5 | 6 | 44 | 288 | 6.55 |
| **Q7** | 4 | 56 | 8.1 | 10.4 | 7.8 | 33 | 349 | 10.58 |
| **Q7** | 5 | 57.7 | 7.5 | 10 | 7.3 | 39 | 362 | 9.28 |
| **Q7** | 6 | 62.5 | 7.2 | 9.6 | 7.5 | 36 | 388 | 10.78 |
| **Q7** | 7 | 64.8 | 6.6 | 10.3 | 7.4 | 37 | 350 | 9.46 |
| **Q7** | 8 | 55.8 | 8.3 | 10.7 | 8 | 36 | 404 | 11.22 |
| **Q7** | 9 | 61.2 | 6.7 | 8.9 | 6.7 | 49 | 397 | 8.1 |
| **Q7** | 10 | 58.4 | 8 | 10.9 | 8.2 | 38 | 447 | 11.76 |
| **Q8** | 1 | 54.4 | 7.9 | 10.4 | 7.6 | 50 | 443 | 8.86 |
| **Q8** | 2 | 60.8 | 7.1 | 8.7 | 6.9 | 50 | 457 | 9.14 |
| **Q8** | 3 | 47 | 11.3 | 13.6 | 10.2 | 31 | 567 | 18.29 |
| **Q8** | 4 | 49.6 | 9.4 | 11.3 | 8.6 | 40 | 494 | 12.35 |
| **Q8** | 5 | 55.8 | 8 | 10.1 | 7.7 | 46 | 464 | 10.09 |
| **Q8** | 6 | 54.5 | 7.9 | 9.8 | 7.3 | 50 | 461 | 9.22 |
| **Q8** | 7 | 46.3 | 9.9 | 12.9 | 9.3 | 30 | 378 | 12.6 |
| **Q8** | 8 | 54.7 | 8 | 10.5 | 7.6 | 53 | 497 | 9.38 |
| **Q8** | 9 | 45.3 | 10.3 | 12.3 | 9.4 | 31 | 417 | 13.45 |
| **Q8** | 10 | 49 | 8.9 | 11.8 | 8.3 | 49 | 483 | 9.86 |
| **Q9** | 1 | 65.3 | 7.9 | 9.7 | 7.2 | 30 | 454 | 15.13 |
| **Q9** | 2 | 60.3 | 8.5 | 10.3 | 7.9 | 28 | 412 | 14.71 |
| **Q9** | 3 | 60.4 | 8.8 | 10.7 | 8.1 | 27 | 427 | 15.81 |
| **Q9** | 4 | 61.4 | 7.7 | 9.7 | 7.4 | 36 | 440 | 12.22 |
| **Q9** | 5 | 61.7 | 7.9 | 10.3 | 7.8 | 32 | 424 | 13.25 |
| **Q9** | 6 | 61.8 | 8.2 | 9.9 | 7.4 | 25 | 357 | 14.28 |
| **Q9** | 7 | 60.1 | 10.1 | 11.8 | 8.1 | 20 | 418 | 20.9 |
| **Q9** | 8 | 67.4 | 6.3 | 7.9 | 6.1 | 45 | 448 | 9.96 |
| **Q9** | 9 | 61.1 | 8.9 | 10 | 7.6 | 23 | 386 | 16.78 |
| **Q9** | 10 | 64.4 | 7 | 9 | 6.8 | 36 | 395 | 10.97 |
| **Q10** | 1 | 48.6 | 11.2 | 13.7 | 10.7 | 24 | 458 | 19.08 |
| **Q10** | 2 | 48.5 | 10.1 | 12.7 | 9.6 | 29 | 426 | 14.69 |
| **Q10** | 3 | 51.7 | 10.6 | 13.2 | 9.9 | 29 | 534 | 18.41 |
| **Q10** | 4 | 38.1 | 13.7 | 15.8 | 12 | 18 | 417 | 23.17 |
| **Q10** | 5 | 51.6 | 11.1 | 13.4 | 10.1 | 21 | 429 | 20.43 |
| **Q10** | 6 | 57.7 | 7.9 | 11.3 | 8.3 | 48 | 526 | 10.96 |
| **Q10** | 7 | 54.1 | 8.5 | 11.2 | 8.5 | 42 | 469 | 11.17 |
| **Q10** | 8 | 52.8 | 10.2 | 12.7 | 9.6 | 31 | 534 | 17.23 |
| **Q10** | 9 | 53.3 | 9.5 | 12.1 | 9.2 | 33 | 495 | 15 |
| **Q10** | 10 | 60.7 | 7.2 | 9.8 | 7.2 | 46 | 451 | 9.8 |
| **Q11** | 1 | 54 | 9 | 11.2 | 8.5 | 43 | 557 | 12.95 |
| **Q11** | 2 | 52.6 | 9.4 | 11 | 8.3 | 39 | 543 | 13.92 |
| **Q11** | 3 | 48.2 | 10.8 | 12.5 | 9.8 | 28 | 484 | 17.29 |
| **Q11** | 4 | 54.6 | 9.8 | 11.5 | 8.8 | 31 | 515 | 16.61 |
| **Q11** | 5 | 48.6 | 10.9 | 12.5 | 9.8 | 33 | 586 | 17.76 |
| **Q11** | 6 | 47.7 | 10.5 | 12 | 9 | 31 | 483 | 15.58 |
| **Q11** | 7 | 58.4 | 7.7 | 10.3 | 7.7 | 48 | 493 | 10.27 |
| **Q11** | 8 | 52.1 | 10.8 | 12.9 | 9.9 | 32 | 617 | 19.28 |
| **Q11** | 9 | 41.6 | 13.2 | 15.2 | 11.2 | 25 | 582 | 23.28 |
| **Q11** | 10 | 52.5 | 9.3 | 10.9 | 8.5 | 38 | 511 | 13.45 |
| **Q12** | 1 | 60.6 | 7.9 | 10.5 | 8.2 | 42 | 528 | 12.57 |
| **Q12** | 2 | 65 | 6.9 | 9.1 | 7.1 | 46 | 498 | 10.83 |
| **Q12** | 3 | 52.1 | 11.8 | 14.3 | 10.5 | 24 | 561 | 23.38 |
| **Q12** | 4 | 66.6 | 6.7 | 9.3 | 7.1 | 42 | 457 | 10.88 |
| **Q12** | 5 | 66.6 | 6.7 | 9.1 | 7.2 | 43 | 477 | 11.09 |
| **Q12** | 6 | 55.3 | 11.3 | 14.1 | 10 | 22 | 506 | 23 |
| **Q12** | 7 | 67.1 | 7 | 9.3 | 7.1 | 42 | 523 | 12.45 |
| **Q12** | 8 | 62.4 | 7.4 | 9.8 | 7.6 | 44 | 499 | 11.34 |
| **Q12** | 9 | 56.9 | 10.1 | 12.5 | 9.4 | 26 | 497 | 19.12 |
| **Q12** | 10 | 51.4 | 10.4 | 12.7 | 9.5 | 26 | 449 | 17.27 |
| **Q13** | 1 | 56.7 | 9.7 | 11.5 | 9.4 | 33 | 572 | 17.33 |
| **Q13** | 2 | 45 | 12.9 | 15.3 | 11.6 | 28 | 669 | 23.89 |
| **Q13** | 3 | 59.3 | 8.2 | 10.1 | 8.3 | 48 | 625 | 13.02 |
| **Q13** | 4 | 60 | 7.3 | 9.8 | 7.5 | 72 | 705 | 9.79 |
| **Q13** | 5 | 60.4 | 7 | 9.6 | 7.3 | 71 | 619 | 8.72 |
| **Q13** | 6 | 63.7 | 6.8 | 8.8 | 7.1 | 75 | 717 | 9.56 |
| **Q13** | 7 | 64.8 | 6.3 | 8.5 | 6.7 | 87 | 734 | 8.44 |
| **Q13** | 8 | 50.4 | 11.8 | 14 | 10.7 | 30 | 673 | 22.43 |
| **Q13** | 9 | 46 | 11.8 | 14.3 | 11.3 | 30 | 601 | 20.03 |
| **Q13** | 10 | 59.4 | 6.7 | 10.3 | 6.9 | 65 | 459 | 7.06 |
| **Q14** | 1 | 41.5 | 12.6 | 15.7 | 11.9 | 15 | 308 | 20.53 |
| **Q14** | 2 | 49.8 | 11.6 | 13.8 | 10.4 | 18 | 383 | 21.28 |
| **Q14** | 3 | 53.9 | 9.4 | 11.8 | 9.4 | 26 | 384 | 14.77 |
| **Q14** | 4 | 52.4 | 9 | 12.7 | 9.1 | 24 | 293 | 12.21 |
| **Q14** | 5 | 51.7 | 8.1 | 10.7 | 7.3 | 37 | 303 | 8.19 |
| **Q14** | 6 | 43.9 | 12.2 | 15.2 | 11.3 | 16 | 328 | 20.5 |
| **Q14** | 7 | 53.9 | 8.1 | 10.8 | 8 | 39 | 369 | 9.46 |
| **Q14** | 8 | 37.8 | 13.1 | 15 | 11.3 | 15 | 309 | 20.6 |
| **Q14** | 9 | 47.2 | 11.4 | 14.6 | 11 | 19 | 364 | 19.16 |
| **Q14** | 10 | 53.2 | 10 | 12.7 | 9.9 | 18 | 298 | 16.56 |
| **Q15** | 1 | 39 | 12.3 | 13.8 | 11.7 | 30 | 542 | 18.07 |
| **Q15** | 2 | 40.7 | 10.8 | 12.4 | 10.1 | 43 | 564 | 13.12 |
| **Q15** | 3 | 46.8 | 9 | 10.6 | 8.3 | 70 | 635 | 9.07 |
| **Q15** | 4 | 35.4 | 12.4 | 14.1 | 11.3 | 35 | 577 | 16.49 |
| **Q15** | 5 | 39 | 11.4 | 13.7 | 10.7 | 24 | 348 | 14.5 |
| **Q15** | 6 | 47.1 | 9.8 | 11.1 | 9.1 | 50 | 628 | 12.56 |
| **Q15** | 7 | 22.3 | 15 | 16.1 | 12.7 | 16 | 311 | 19.44 |
| **Q15** | 8 | 44.2 | 9.1 | 9.6 | 7.6 | 37 | 295 | 7.97 |
| **Q15** | 9 | 27.5 | 14.8 | 16.7 | 12.9 | 26 | 559 | 21.5 |
| **Q15** | 10 | 42.2 | 10.9 | 12.5 | 10.1 | 39 | 559 | 14.33 |

### **S2.3.2 Gemini (n = 150)**

| **PromptID** | **Run** | **FRE** | **FKGL** | **Gunning Fog** | **SMOG** | **Sentences** | **Words** | **Words / sentence** |
| --- | --- | --- | --- | --- | --- | --- | --- | --- |
| **Q1** | 1 | 57.9 | 8.8 | 11.1 | 8.5 | 31 | 450 | 14.52 |
| **Q1** | 2 | 64.3 | 8.3 | 10.3 | 7.8 | 30 | 482 | 16.07 |
| **Q1** | 3 | 67.5 | 7.6 | 9.9 | 7.5 | 38 | 576 | 15.16 |
| **Q1** | 4 | 58.4 | 9.8 | 12.1 | 9 | 27 | 513 | 19 |
| **Q1** | 5 | 65.1 | 8.1 | 10 | 7.8 | 32 | 506 | 15.81 |
| **Q1** | 6 | 66.7 | 7.4 | 9.4 | 7.6 | 35 | 482 | 13.77 |
| **Q1** | 7 | 58.6 | 9.2 | 10.9 | 8.3 | 31 | 519 | 16.74 |
| **Q1** | 8 | 64.2 | 7.4 | 9.2 | 7.3 | 40 | 505 | 12.63 |
| **Q1** | 9 | 68.3 | 7.3 | 9.2 | 7.1 | 35 | 503 | 14.37 |
| **Q1** | 10 | 68.6 | 7 | 9.3 | 7.2 | 36 | 481 | 13.36 |
| **Q2** | 1 | 65.6 | 7.7 | 9.4 | 7.3 | 29 | 419 | 14.45 |
| **Q2** | 2 | 62.5 | 8.2 | 10.1 | 7.9 | 27 | 402 | 14.89 |
| **Q2** | 3 | 61.3 | 8.8 | 10.7 | 7.8 | 30 | 500 | 16.67 |
| **Q2** | 4 | 58.7 | 8.6 | 10.2 | 8.2 | 34 | 482 | 14.18 |
| **Q2** | 5 | 61.7 | 8.1 | 9.1 | 7.3 | 35 | 479 | 13.69 |
| **Q2** | 6 | 58.5 | 9.5 | 11.8 | 8.9 | 10 | 178 | 17.8 |
| **Q2** | 7 | 66.4 | 7.9 | 9.8 | 7.7 | 32 | 502 | 15.69 |
| **Q2** | 8 | 65.9 | 7.6 | 9.5 | 7.1 | 33 | 475 | 14.39 |
| **Q2** | 9 | 69.7 | 6.9 | 9.2 | 7 | 36 | 486 | 13.5 |
| **Q2** | 10 | 60.5 | 8.2 | 9.2 | 7.7 | 36 | 486 | 13.5 |
| **Q3** | 1 | 49.2 | 9.6 | 11.5 | 8.8 | 35 | 450 | 12.86 |
| **Q3** | 2 | 45.3 | 10.5 | 11.9 | 9.2 | 34 | 488 | 14.35 |
| **Q3** | 3 | 51.8 | 9.9 | 11.8 | 9.1 | 33 | 516 | 15.64 |
| **Q3** | 4 | 46.8 | 10.3 | 13 | 9.8 | 33 | 480 | 14.55 |
| **Q3** | 5 | 55.2 | 8.7 | 10.8 | 8.3 | 37 | 475 | 12.84 |
| **Q3** | 6 | 49.5 | 9.4 | 11.1 | 8.7 | 42 | 511 | 12.17 |
| **Q3** | 7 | 50.6 | 9.5 | 11.1 | 8.8 | 39 | 510 | 13.08 |
| **Q3** | 8 | 50.3 | 9.9 | 12 | 9.2 | 34 | 500 | 14.71 |
| **Q3** | 9 | 44.7 | 10.3 | 11.7 | 8.9 | 37 | 486 | 13.14 |
| **Q3** | 10 | 47.1 | 9.9 | 11.7 | 8.9 | 37 | 476 | 12.86 |
| **Q4** | 1 | 54.3 | 9.6 | 11 | 8.4 | 28 | 444 | 15.86 |
| **Q4** | 2 | 60.7 | 8.1 | 9.5 | 7.4 | 33 | 446 | 13.52 |
| **Q4** | 3 | 52.9 | 9.6 | 11 | 8.6 | 29 | 437 | 15.07 |
| **Q4** | 4 | 53.9 | 10 | 11.9 | 9.1 | 31 | 527 | 17 |
| **Q4** | 5 | 45.9 | 11.1 | 13 | 10 | 29 | 490 | 16.9 |
| **Q4** | 6 | 60.1 | 8.7 | 10.2 | 7.9 | 32 | 496 | 15.5 |
| **Q4** | 7 | 56.4 | 9.7 | 11.3 | 8.5 | 31 | 536 | 17.29 |
| **Q4** | 8 | 61.4 | 8.2 | 10.6 | 8 | 32 | 454 | 14.19 |
| **Q4** | 9 | 63.6 | 8 | 10.1 | 7.5 | 32 | 462 | 14.44 |
| **Q4** | 10 | 61 | 8.8 | 10.6 | 8.2 | 30 | 494 | 16.47 |
| **Q5** | 1 | 52.9 | 10.2 | 12.9 | 9.9 | 23 | 399 | 17.35 |
| **Q5** | 2 | 67.1 | 7.4 | 9.5 | 7.5 | 28 | 396 | 14.14 |
| **Q5** | 3 | 58.3 | 9.2 | 12 | 9 | 24 | 394 | 16.42 |
| **Q5** | 4 | 61.1 | 8.5 | 10.5 | 8.4 | 26 | 396 | 15.23 |
| **Q5** | 5 | 58.4 | 9.2 | 10.7 | 8.6 | 29 | 481 | 16.59 |
| **Q5** | 6 | 60.6 | 8.3 | 10.1 | 7.9 | 28 | 390 | 13.93 |
| **Q5** | 7 | 58.6 | 9 | 10.9 | 8.8 | 29 | 454 | 15.66 |
| **Q5** | 8 | 60.5 | 8.7 | 10.7 | 8.4 | 26 | 402 | 15.46 |
| **Q5** | 9 | 56.2 | 9 | 10.4 | 8.2 | 28 | 403 | 14.39 |
| **Q5** | 10 | 61.4 | 8.6 | 11 | 8.6 | 28 | 438 | 15.64 |
| **Q6** | 1 | 53.1 | 9.3 | 10.8 | 8.8 | 30 | 410 | 13.67 |
| **Q6** | 2 | 59.3 | 9.4 | 11.8 | 9.1 | 28 | 500 | 17.86 |
| **Q6** | 3 | 47.6 | 10.7 | 12.5 | 9.6 | 23 | 380 | 16.52 |
| **Q6** | 4 | 54 | 9.6 | 11.8 | 9.2 | 28 | 433 | 15.46 |
| **Q6** | 5 | 57.6 | 9.1 | 11.2 | 8.9 | 30 | 470 | 15.67 |
| **Q6** | 6 | 57.5 | 10.1 | 12.3 | 9.2 | 23 | 448 | 19.48 |
| **Q6** | 7 | 58.9 | 8.8 | 11.5 | 8.7 | 30 | 458 | 15.27 |
| **Q6** | 8 | 57.6 | 9.2 | 11.3 | 8.7 | 31 | 496 | 16 |
| **Q6** | 9 | 49 | 11.1 | 13 | 10 | 29 | 550 | 18.97 |
| **Q6** | 10 | 54.6 | 10.2 | 12.5 | 9.6 | 25 | 456 | 18.24 |
| **Q7** | 1 | 44.6 | 11 | 12.8 | 10 | 29 | 463 | 15.97 |
| **Q7** | 2 | 49.7 | 10 | 11.6 | 9.2 | 32 | 472 | 14.75 |
| **Q7** | 3 | 48.5 | 11 | 12.4 | 9.8 | 25 | 457 | 18.28 |
| **Q7** | 4 | 48 | 10.4 | 12.4 | 9.4 | 27 | 418 | 15.48 |
| **Q7** | 5 | 51.4 | 9.6 | 11.2 | 8.9 | 30 | 428 | 14.27 |
| **Q7** | 6 | 48.7 | 10.4 | 12.2 | 9.6 | 30 | 470 | 15.67 |
| **Q7** | 7 | 49.6 | 10.5 | 12.5 | 9.8 | 28 | 468 | 16.71 |
| **Q7** | 8 | 47 | 10.7 | 11.9 | 9.6 | 27 | 437 | 16.19 |
| **Q7** | 9 | 49.1 | 10.8 | 12.5 | 9.8 | 28 | 490 | 17.5 |
| **Q7** | 10 | 48.1 | 10.3 | 12.3 | 9.7 | 29 | 439 | 15.14 |
| **Q8** | 1 | 50.8 | 10.5 | 12.5 | 9.9 | 31 | 544 | 17.55 |
| **Q8** | 2 | 45.7 | 10.8 | 12.8 | 10 | 33 | 523 | 15.85 |
| **Q8** | 3 | 52.1 | 10.1 | 12.2 | 9.6 | 28 | 466 | 16.64 |
| **Q8** | 4 | 52.3 | 9.9 | 12.3 | 9.5 | 32 | 507 | 15.84 |
| **Q8** | 5 | 52.5 | 9.6 | 11.9 | 9.2 | 34 | 505 | 14.85 |
| **Q8** | 6 | 51.2 | 9.7 | 12.1 | 9.5 | 34 | 485 | 14.26 |
| **Q8** | 7 | 52.4 | 10 | 11.6 | 9.2 | 34 | 552 | 16.24 |
| **Q8** | 8 | 50 | 10.2 | 11.7 | 9.1 | 31 | 483 | 15.58 |
| **Q8** | 9 | 54.8 | 9.2 | 11.4 | 8.9 | 37 | 533 | 14.41 |
| **Q8** | 10 | 50.5 | 10.6 | 12.2 | 9.5 | 26 | 457 | 17.58 |
| **Q9** | 1 | 56.8 | 8.6 | 10 | 8 | 37 | 479 | 12.95 |
| **Q9** | 2 | 59.8 | 8.9 | 10.5 | 7.9 | 34 | 549 | 16.15 |
| **Q9** | 3 | 53.5 | 9.4 | 11.1 | 8.7 | 38 | 551 | 14.5 |
| **Q9** | 4 | 54.2 | 9.1 | 11 | 8.5 | 40 | 542 | 13.55 |
| **Q9** | 5 | 57.8 | 9.3 | 11.4 | 8.8 | 30 | 493 | 16.43 |
| **Q9** | 6 | 62.2 | 7.8 | 9.7 | 7.3 | 38 | 490 | 12.89 |
| **Q9** | 7 | 52.2 | 9.8 | 11.1 | 9 | 31 | 482 | 15.55 |
| **Q9** | 8 | 57.5 | 8.8 | 10.8 | 8.5 | 40 | 578 | 14.45 |
| **Q9** | 9 | 57.1 | 9 | 11.1 | 8.8 | 34 | 506 | 14.88 |
| **Q9** | 10 | 54.8 | 9.3 | 11.1 | 8.5 | 37 | 549 | 14.84 |
| **Q10** | 1 | 41.4 | 11.6 | 14.8 | 11.3 | 32 | 535 | 16.72 |
| **Q10** | 2 | 44.8 | 11.4 | 13.3 | 10.3 | 30 | 534 | 17.8 |
| **Q10** | 3 | 44 | 11.5 | 13.9 | 10.8 | 28 | 498 | 17.79 |
| **Q10** | 4 | 47.4 | 11.1 | 13.2 | 10.4 | 30 | 540 | 18 |
| **Q10** | 5 | 39.6 | 12.8 | 14.6 | 11.5 | 21 | 427 | 20.33 |
| **Q10** | 6 | 47.9 | 10.1 | 11.8 | 9.2 | 38 | 536 | 14.11 |
| **Q10** | 7 | 40.7 | 12.2 | 14.6 | 11.3 | 31 | 575 | 18.55 |
| **Q10** | 8 | 38.7 | 12.7 | 15.1 | 11.8 | 27 | 525 | 19.44 |
| **Q10** | 9 | 42.1 | 11.5 | 13.2 | 10.5 | 30 | 493 | 16.43 |
| **Q10** | 10 | 41.8 | 12 | 14 | 11.1 | 29 | 537 | 18.52 |
| **Q11** | 1 | 50.7 | 10.2 | 12 | 9.3 | 34 | 549 | 16.15 |
| **Q11** | 2 | 46.3 | 10.9 | 11.9 | 9.6 | 33 | 543 | 16.45 |
| **Q11** | 3 | 57.2 | 9.4 | 10.9 | 8.4 | 29 | 486 | 16.76 |
| **Q11** | 4 | 45.3 | 11.2 | 12.2 | 10 | 29 | 499 | 17.21 |
| **Q11** | 5 | 50.1 | 10.4 | 11.6 | 9.3 | 29 | 487 | 16.79 |
| **Q11** | 6 | 51.9 | 10.3 | 11.3 | 9 | 27 | 464 | 17.19 |
| **Q11** | 7 | 53.2 | 9.6 | 10.5 | 8.3 | 34 | 513 | 15.09 |
| **Q11** | 8 | 49.8 | 10.3 | 11.6 | 9.2 | 30 | 483 | 16.1 |
| **Q11** | 9 | 48.2 | 11 | 12.2 | 9.7 | 29 | 518 | 17.86 |
| **Q11** | 10 | 47.8 | 10.9 | 12.3 | 9.5 | 27 | 471 | 17.44 |
| **Q12** | 1 | 54.3 | 9.9 | 12 | 9 | 28 | 471 | 16.82 |
| **Q12** | 2 | 61 | 8.3 | 10.5 | 8 | 33 | 471 | 14.27 |
| **Q12** | 3 | 55 | 9.7 | 11.4 | 9.1 | 29 | 484 | 16.69 |
| **Q12** | 4 | 45.7 | 11.8 | 13.7 | 10.4 | 27 | 531 | 19.67 |
| **Q12** | 5 | 51.1 | 9.9 | 11.6 | 9 | 28 | 429 | 15.32 |
| **Q12** | 6 | 53.8 | 9.7 | 11.8 | 9 | 24 | 382 | 15.92 |
| **Q12** | 7 | 56.9 | 9.3 | 11.4 | 8.6 | 28 | 443 | 15.82 |
| **Q12** | 8 | 57.5 | 8.7 | 10.5 | 8.1 | 34 | 469 | 13.79 |
| **Q12** | 9 | 48.3 | 11.2 | 13.6 | 10.1 | 24 | 455 | 18.96 |
| **Q12** | 10 | 57 | 9.6 | 11.9 | 8.7 | 29 | 497 | 17.14 |
| **Q13** | 1 | 53.6 | 9.5 | 10.2 | 8.8 | 37 | 552 | 14.92 |
| **Q13** | 2 | 51.6 | 10 | 11.6 | 9.2 | 29 | 457 | 15.76 |
| **Q13** | 3 | 50 | 9.9 | 11.6 | 9.5 | 35 | 508 | 14.51 |
| **Q13** | 4 | 57.3 | 9.4 | 10.9 | 8.9 | 31 | 514 | 16.58 |
| **Q13** | 5 | 54 | 9.9 | 11.4 | 8.9 | 32 | 542 | 16.94 |
| **Q13** | 6 | 61.1 | 9 | 10.4 | 7.7 | 29 | 497 | 17.14 |
| **Q13** | 7 | 57.6 | 8.5 | 10.5 | 7.9 | 39 | 508 | 13.03 |
| **Q13** | 8 | 46.7 | 11 | 12.3 | 9.7 | 29 | 496 | 17.1 |
| **Q13** | 9 | 55.5 | 8.9 | 10.2 | 8.5 | 33 | 449 | 13.61 |
| **Q13** | 10 | 54.6 | 9.4 | 11.6 | 8.8 | 19 | 286 | 15.05 |
| **Q14** | 1 | 49.7 | 10.2 | 12.5 | 9.6 | 26 | 408 | 15.69 |
| **Q14** | 2 | 49.2 | 11 | 13.5 | 10.1 | 24 | 445 | 18.54 |
| **Q14** | 3 | 59.1 | 9 | 11.2 | 8.7 | 25 | 404 | 16.16 |
| **Q14** | 4 | 45.9 | 10.7 | 13.2 | 10.1 | 28 | 436 | 15.57 |
| **Q14** | 5 | 57.5 | 8.9 | 12 | 9.3 | 27 | 403 | 14.93 |
| **Q14** | 6 | 54.1 | 10.1 | 12.9 | 9.9 | 28 | 491 | 17.54 |
| **Q14** | 7 | 49.2 | 10.6 | 14.1 | 10.6 | 27 | 455 | 16.85 |
| **Q14** | 8 | 55.5 | 9.5 | 12.1 | 9.2 | 24 | 388 | 16.17 |
| **Q14** | 9 | 48.2 | 11 | 14.1 | 10.6 | 24 | 430 | 17.92 |
| **Q14** | 10 | 42.3 | 12.1 | 14.9 | 11.3 | 24 | 458 | 19.08 |
| **Q15** | 1 | 40.8 | 12.2 | 14.2 | 10.9 | 29 | 545 | 18.79 |
| **Q15** | 2 | 42.3 | 11.6 | 12.8 | 10.1 | 30 | 514 | 17.13 |
| **Q15** | 3 | 47.9 | 10.6 | 12.8 | 10 | 38 | 613 | 16.13 |
| **Q15** | 4 | 43.6 | 11.6 | 13.3 | 10.6 | 30 | 532 | 17.73 |
| **Q15** | 5 | 45.9 | 10.7 | 12.4 | 9.9 | 35 | 536 | 15.31 |
| **Q15** | 6 | 30.2 | 13.8 | 15.7 | 12.3 | 29 | 554 | 19.1 |
| **Q15** | 7 | 43.9 | 11.2 | 12.6 | 10.2 | 35 | 573 | 16.37 |
| **Q15** | 8 | 38.2 | 11.9 | 12.8 | 10.4 | 29 | 466 | 16.07 |
| **Q15** | 9 | 41.8 | 11.2 | 13 | 10.2 | 36 | 552 | 15.33 |
| **Q15** | 10 | 35.7 | 12.6 | 14.1 | 11 | 33 | 569 | 17.24 |

### **S2.3.3 Claude (n = 150)**

| **PromptID** | **Run** | **FRE** | **FKGL** | **Gunning Fog** | **SMOG** | **Sentences** | **Words** | **Words / sentence** |
| --- | --- | --- | --- | --- | --- | --- | --- | --- |
| **Q1** | 1 | 44 | 12.1 | 13.9 | 10.6 | 30 | 603 | 20.1 |
| **Q1** | 2 | 51 | 11.1 | 13.7 | 10.2 | 33 | 665 | 20.15 |
| **Q1** | 3 | 56.9 | 9.4 | 11.6 | 9.1 | 32 | 522 | 16.31 |
| **Q1** | 4 | 44 | 12.1 | 13.9 | 10.6 | 30 | 603 | 20.1 |
| **Q1** | 5 | 49.9 | 11.5 | 13.5 | 10 | 35 | 736 | 21.03 |
| **Q1** | 6 | 45.6 | 11.9 | 14.6 | 10.8 | 31 | 622 | 20.06 |
| **Q1** | 7 | 41.6 | 13.5 | 15.5 | 11.5 | 23 | 562 | 24.43 |
| **Q1** | 8 | 50.7 | 11.4 | 13.2 | 9.7 | 37 | 783 | 21.16 |
| **Q1** | 9 | 49.9 | 11.4 | 14.3 | 10.5 | 32 | 660 | 20.63 |
| **Q1** | 10 | 58.3 | 8.3 | 10.9 | 8.1 | 46 | 590 | 12.83 |
| **Q2** | 1 | 53.4 | 11 | 12.9 | 9.5 | 37 | 777 | 21 |
| **Q2** | 2 | 51.9 | 11.4 | 12.9 | 9.2 | 32 | 698 | 21.81 |
| **Q2** | 3 | 50.9 | 10.6 | 12.6 | 9.2 | 27 | 479 | 17.74 |
| **Q2** | 4 | 51.8 | 11.2 | 13.7 | 9.8 | 22 | 455 | 20.68 |
| **Q2** | 5 | 61.7 | 8.6 | 10.7 | 8.2 | 38 | 600 | 15.79 |
| **Q2** | 6 | 51.9 | 10.7 | 13.5 | 9.9 | 30 | 559 | 18.63 |
| **Q2** | 7 | 57.8 | 9 | 10.9 | 8.3 | 36 | 555 | 15.42 |
| **Q2** | 8 | 62.8 | 8.6 | 10.5 | 8 | 30 | 490 | 16.33 |
| **Q2** | 9 | 47.6 | 10.9 | 12.4 | 9.5 | 27 | 467 | 17.3 |
| **Q2** | 10 | 74.1 | 6.1 | 8.5 | 6.6 | 65 | 830 | 12.77 |
| **Q3** | 1 | 36.5 | 13.1 | 15.6 | 11.8 | 27 | 531 | 19.67 |
| **Q3** | 2 | 36.2 | 11.7 | 14.5 | 10.8 | 28 | 398 | 14.21 |
| **Q3** | 3 | 30.5 | 13.9 | 17.5 | 12.9 | 27 | 535 | 19.81 |
| **Q3** | 4 | 34.9 | 11.5 | 13.1 | 9.7 | 45 | 558 | 12.4 |
| **Q3** | 5 | 33.1 | 12.8 | 15.9 | 11.8 | 43 | 722 | 16.79 |
| **Q3** | 6 | 31.7 | 13.3 | 16 | 11.6 | 35 | 622 | 17.77 |
| **Q3** | 7 | 27.5 | 14.8 | 17.6 | 13.3 | 29 | 633 | 21.83 |
| **Q3** | 8 | 40.6 | 12.4 | 15.6 | 11.5 | 32 | 618 | 19.31 |
| **Q3** | 9 | 32.1 | 13 | 15.2 | 11.7 | 42 | 718 | 17.1 |
| **Q3** | 10 | 34.5 | 12.9 | 15.3 | 11.6 | 29 | 519 | 17.9 |
| **Q4** | 1 | 56.4 | 8.9 | 11.4 | 8.4 | 37 | 516 | 13.95 |
| **Q4** | 2 | 57.8 | 8.7 | 10.6 | 7.9 | 34 | 475 | 13.97 |
| **Q4** | 3 | 57 | 8.8 | 11.3 | 8.3 | 33 | 460 | 13.94 |
| **Q4** | 4 | 48.7 | 11 | 13.2 | 9.6 | 18 | 332 | 18.44 |
| **Q4** | 5 | 66 | 7.1 | 9.2 | 6.8 | 34 | 412 | 12.12 |
| **Q4** | 6 | 51.1 | 10.4 | 12.6 | 9.1 | 22 | 376 | 17.09 |
| **Q4** | 7 | 64.5 | 7.7 | 9.4 | 6.9 | 27 | 376 | 13.93 |
| **Q4** | 8 | 49.3 | 12.2 | 14.4 | 10 | 18 | 418 | 23.22 |
| **Q4** | 9 | 51.7 | 10.4 | 12.3 | 9.2 | 22 | 390 | 17.73 |
| **Q4** | 10 | 53.1 | 10.5 | 12.8 | 9.3 | 23 | 433 | 18.83 |
| **Q5** | 1 | 43.7 | 11.2 | 13.2 | 10.2 | 25 | 410 | 16.4 |
| **Q5** | 2 | 62 | 8.5 | 10.4 | 7.8 | 35 | 547 | 15.63 |
| **Q5** | 3 | 53.1 | 9.2 | 11.1 | 8.4 | 28 | 373 | 13.32 |
| **Q5** | 4 | 42.7 | 11.9 | 14.3 | 10.8 | 24 | 442 | 18.42 |
| **Q5** | 5 | 52.6 | 10.3 | 12.2 | 9.1 | 25 | 442 | 17.68 |
| **Q5** | 6 | 56.7 | 10.3 | 13.3 | 9.8 | 23 | 457 | 19.87 |
| **Q5** | 7 | 40.2 | 13.1 | 15 | 11.2 | 19 | 413 | 21.74 |
| **Q5** | 8 | 38.8 | 12.9 | 15.1 | 11.3 | 15 | 302 | 20.13 |
| **Q5** | 9 | 53.2 | 9.8 | 12.9 | 9.5 | 27 | 435 | 16.11 |
| **Q5** | 10 | 61.8 | 8 | 10.3 | 7.8 | 34 | 460 | 13.53 |
| **Q6** | 1 | 49.6 | 12 | 14.4 | 10.3 | 21 | 475 | 22.62 |
| **Q6** | 2 | 52.2 | 9.9 | 12.6 | 9.2 | 28 | 441 | 15.75 |
| **Q6** | 3 | 48.6 | 11.1 | 14.4 | 10.5 | 20 | 373 | 18.65 |
| **Q6** | 4 | 55.3 | 9 | 11.8 | 8.7 | 25 | 343 | 13.72 |
| **Q6** | 5 | 32.4 | 14.6 | 17.7 | 13.1 | 17 | 400 | 23.53 |
| **Q6** | 6 | 39.9 | 13.3 | 16.4 | 12.1 | 17 | 383 | 22.53 |
| **Q6** | 7 | 51.4 | 10.6 | 14.1 | 10.5 | 23 | 416 | 18.09 |
| **Q6** | 8 | 58.3 | 9.2 | 12.2 | 9 | 26 | 426 | 16.38 |
| **Q6** | 9 | 53.9 | 9.5 | 12.7 | 9.4 | 26 | 397 | 15.27 |
| **Q6** | 10 | 44.3 | 11.4 | 14.8 | 10.8 | 23 | 398 | 17.3 |
| **Q7** | 1 | 40.7 | 11.8 | 14.2 | 11 | 27 | 458 | 16.96 |
| **Q7** | 2 | 44.3 | 10.8 | 13.3 | 10 | 32 | 484 | 15.13 |
| **Q7** | 3 | 44.2 | 12.2 | 15.2 | 11.2 | 34 | 696 | 20.47 |
| **Q7** | 4 | 45.3 | 12.2 | 14 | 10.7 | 24 | 505 | 21.04 |
| **Q7** | 5 | 43.6 | 11.8 | 13.7 | 10.7 | 26 | 486 | 18.69 |
| **Q7** | 6 | 54.3 | 9.9 | 12.7 | 9.7 | 34 | 578 | 17 |
| **Q7** | 7 | 35.4 | 13.4 | 16.1 | 12 | 18 | 371 | 20.61 |
| **Q7** | 8 | 30.2 | 14.3 | 16.6 | 12.9 | 19 | 398 | 20.95 |
| **Q7** | 9 | 40.8 | 11.5 | 13.8 | 10.6 | 29 | 463 | 15.97 |
| **Q7** | 10 | 33.8 | 15.4 | 19.1 | 13.6 | 7 | 193 | 27.57 |
| **Q8** | 1 | 44.9 | 10.6 | 13.1 | 9.7 | 46 | 667 | 14.5 |
| **Q8** | 2 | 49.4 | 9.8 | 12.1 | 9.2 | 43 | 586 | 13.63 |
| **Q8** | 3 | 49.9 | 10.7 | 14 | 10.4 | 42 | 747 | 17.79 |
| **Q8** | 4 | 49.3 | 10.6 | 13.4 | 9.9 | 42 | 712 | 16.95 |
| **Q8** | 5 | 45 | 10.8 | 13.3 | 9.8 | 46 | 700 | 15.22 |
| **Q8** | 6 | 40.6 | 11.9 | 14 | 10.5 | 36 | 622 | 17.28 |
| **Q8** | 7 | 50.5 | 10.2 | 12.6 | 9.3 | 45 | 720 | 16 |
| **Q8** | 8 | 39.9 | 12.7 | 14.9 | 11.3 | 40 | 810 | 20.25 |
| **Q8** | 9 | 44.6 | 11 | 14 | 10.2 | 33 | 531 | 16.09 |
| **Q8** | 10 | 48.1 | 11 | 12.9 | 10 | 37 | 668 | 18.05 |
| **Q9** | 1 | 54.5 | 8.5 | 10.8 | 7.9 | 43 | 491 | 11.42 |
| **Q9** | 2 | 54.5 | 9.2 | 12.2 | 8.9 | 36 | 517 | 14.36 |
| **Q9** | 3 | 44.7 | 12.2 | 15.4 | 11.4 | 16 | 335 | 20.94 |
| **Q9** | 4 | 57.8 | 9.4 | 12.3 | 9.1 | 26 | 441 | 16.96 |
| **Q9** | 5 | 52.5 | 10.1 | 13 | 9.8 | 38 | 637 | 16.76 |
| **Q9** | 6 | 52.3 | 9.8 | 12.5 | 9.1 | 27 | 414 | 15.33 |
| **Q9** | 7 | 52.3 | 9.2 | 11.9 | 8.8 | 39 | 505 | 12.95 |
| **Q9** | 8 | 50.6 | 10.3 | 12.7 | 9.6 | 34 | 556 | 16.35 |
| **Q9** | 9 | 55.2 | 9.6 | 12.3 | 9.1 | 28 | 458 | 16.36 |
| **Q9** | 10 | 58.2 | 9.4 | 12.3 | 8.9 | 35 | 600 | 17.14 |
| **Q10** | 1 | 24 | 15.9 | 19.4 | 14.4 | 22 | 528 | 24 |
| **Q10** | 2 | 35.1 | 13.7 | 17.2 | 12.6 | 25 | 538 | 21.52 |
| **Q10** | 3 | 31.9 | 13.3 | 16.6 | 12.3 | 28 | 507 | 18.11 |
| **Q10** | 4 | 36 | 12.5 | 15.6 | 11.6 | 34 | 584 | 17.18 |
| **Q10** | 5 | 28.9 | 14.5 | 16.9 | 12.6 | 21 | 442 | 21.05 |
| **Q10** | 6 | 36.5 | 12.1 | 15.2 | 11 | 36 | 576 | 16 |
| **Q10** | 7 | 38.5 | 11.1 | 14 | 10 | 39 | 500 | 12.82 |
| **Q10** | 8 | 38.8 | 12.4 | 15.2 | 11.1 | 26 | 474 | 18.23 |
| **Q10** | 9 | 33.6 | 13 | 16.2 | 12.1 | 35 | 620 | 17.71 |
| **Q10** | 10 | 44.5 | 10.3 | 12.7 | 9.6 | 36 | 466 | 12.94 |
| **Q11** | 1 | 51.2 | 9.4 | 11.6 | 8.6 | 36 | 479 | 13.31 |
| **Q11** | 2 | 44 | 10.8 | 13.8 | 10.1 | 39 | 582 | 14.92 |
| **Q11** | 3 | 38.8 | 11.1 | 13 | 9.7 | 53 | 689 | 13 |
| **Q11** | 4 | 36.4 | 12.5 | 15.4 | 11.5 | 46 | 802 | 17.43 |
| **Q11** | 5 | 60.7 | 7.6 | 9.8 | 7.5 | 57 | 633 | 11.11 |
| **Q11** | 6 | 46.6 | 10.1 | 12.5 | 9 | 48 | 652 | 13.58 |
| **Q11** | 7 | 49.7 | 10.5 | 12.9 | 9.7 | 48 | 805 | 16.77 |
| **Q11** | 8 | 39.9 | 12.7 | 15.7 | 11.4 | 29 | 582 | 20.07 |
| **Q11** | 9 | 42.4 | 12.6 | 15.2 | 11.1 | 25 | 524 | 20.96 |
| **Q11** | 10 | 35.7 | 13.8 | 16.4 | 12.2 | 27 | 596 | 22.07 |
| **Q12** | 1 | 53.5 | 9.1 | 11.1 | 8.3 | 40 | 539 | 13.48 |
| **Q12** | 2 | 49.9 | 11.6 | 14 | 10.2 | 22 | 466 | 21.18 |
| **Q12** | 3 | 49.8 | 11.4 | 14 | 10.1 | 21 | 431 | 20.52 |
| **Q12** | 4 | 48.7 | 10.5 | 11.9 | 9 | 34 | 552 | 16.24 |
| **Q12** | 5 | 56.6 | 9.3 | 11.6 | 8.6 | 33 | 519 | 15.73 |
| **Q12** | 6 | 48.2 | 11 | 12.4 | 9.2 | 24 | 433 | 18.04 |
| **Q12** | 7 | 52 | 10.1 | 11.5 | 8.7 | 34 | 562 | 16.53 |
| **Q12** | 8 | 46.7 | 12.1 | 14.1 | 10.7 | 28 | 599 | 21.39 |
| **Q12** | 9 | 39.2 | 12.1 | 13.6 | 10.2 | 35 | 608 | 17.37 |
| **Q12** | 10 | 50.2 | 10.6 | 12 | 8.9 | 35 | 615 | 17.57 |
| **Q13** | 1 | 54.3 | 9.2 | 12.1 | 9.1 | 44 | 626 | 14.23 |
| **Q13** | 2 | 45.8 | 11.3 | 13.9 | 10.5 | 33 | 587 | 17.79 |
| **Q13** | 3 | 47.5 | 11.1 | 13.2 | 10.2 | 26 | 469 | 18.04 |
| **Q13** | 4 | 46.5 | 10.6 | 13.6 | 10.1 | 43 | 667 | 15.51 |
| **Q13** | 5 | 50.9 | 10.6 | 14.1 | 10.3 | 29 | 518 | 17.86 |
| **Q13** | 6 | 41.2 | 12.7 | 15.2 | 11.6 | 25 | 526 | 21.04 |
| **Q13** | 7 | 53.9 | 8.7 | 11.2 | 8.7 | 23 | 272 | 11.83 |
| **Q13** | 8 | 45.8 | 10.5 | 13.1 | 9.7 | 42 | 618 | 14.71 |
| **Q13** | 9 | 42 | 12.6 | 15.8 | 11.6 | 30 | 630 | 21 |
| **Q13** | 10 | 45.1 | 11.8 | 13.7 | 10.2 | 26 | 506 | 19.46 |
| **Q14** | 1 | 41.1 | 11.8 | 15.4 | 11.2 | 21 | 363 | 17.29 |
| **Q14** | 2 | 36.1 | 12.9 | 16 | 12 | 16 | 300 | 18.75 |
| **Q14** | 3 | 46.1 | 10.9 | 14.3 | 10.4 | 20 | 325 | 16.25 |
| **Q14** | 4 | 49.3 | 10.1 | 13.4 | 10 | 29 | 436 | 15.03 |
| **Q14** | 5 | 39.6 | 11.9 | 14.3 | 11.1 | 18 | 298 | 16.56 |
| **Q14** | 6 | 40.8 | 12.9 | 16.6 | 12.2 | 14 | 303 | 21.64 |
| **Q14** | 7 | 50 | 10.6 | 13.9 | 10.4 | 24 | 421 | 17.54 |
| **Q14** | 8 | 46.5 | 11.2 | 14.3 | 10.7 | 23 | 414 | 18 |
| **Q14** | 9 | 49.5 | 11.3 | 15.4 | 11.4 | 17 | 341 | 20.06 |
| **Q14** | 10 | 42.9 | 11.4 | 15.1 | 11 | 19 | 317 | 16.68 |
| **Q15** | 1 | 20.8 | 15.6 | 17.9 | 13.5 | 20 | 422 | 21.1 |
| **Q15** | 2 | 28.3 | 14.3 | 16.5 | 12.2 | 27 | 537 | 19.89 |
| **Q15** | 3 | 34.1 | 12.5 | 15 | 10.9 | 37 | 600 | 16.22 |
| **Q15** | 4 | 30.2 | 13.3 | 15.8 | 11.7 | 43 | 728 | 16.93 |
| **Q15** | 5 | 31.1 | 13.8 | 16.7 | 12.5 | 32 | 630 | 19.69 |
| **Q15** | 6 | 23 | 15.6 | 17.2 | 13.1 | 22 | 487 | 22.14 |
| **Q15** | 7 | 18.3 | 15.9 | 17.7 | 13.4 | 29 | 611 | 21.07 |
| **Q15** | 8 | 21.8 | 15.5 | 17.1 | 13 | 29 | 620 | 21.38 |
| **Q15** | 9 | 25.6 | 14.8 | 17.5 | 13.1 | 24 | 498 | 20.75 |
| **Q15** | 10 | 35.7 | 12.6 | 14.6 | 10.9 | 35 | 608 | 17.37 |

### **S2.3.4 DeepSeek (n = 150)**

| **PromptID** | **Run** | **FRE** | **FKGL** | **Gunning Fog** | **SMOG** | **Sentences** | **Words** | **Words / sentence** |
| --- | --- | --- | --- | --- | --- | --- | --- | --- |
| **Q1** | 1 | 71.2 | 5.8 | 8.6 | 6.4 | 65 | 652 | 10.03 |
| **Q1** | 2 | 67.2 | 7.2 | 9.4 | 7.2 | 52 | 689 | 13.25 |
| **Q1** | 3 | 70.5 | 7 | 9.1 | 6.7 | 46 | 661 | 14.37 |
| **Q1** | 4 | 67.7 | 7.4 | 10.2 | 7.5 | 47 | 672 | 14.3 |
| **Q1** | 5 | 58.1 | 10.2 | 12.8 | 9.3 | 36 | 727 | 20.19 |
| **Q1** | 6 | 50.1 | 12 | 14.1 | 10.3 | 17 | 390 | 22.94 |
| **Q1** | 7 | 66.8 | 7.1 | 9.9 | 7.5 | 33 | 426 | 12.91 |
| **Q1** | 8 | 59 | 9.8 | 12.3 | 8.9 | 36 | 691 | 19.19 |
| **Q1** | 9 | 62.2 | 9.6 | 12.1 | 8.9 | 27 | 541 | 20.04 |
| **Q1** | 10 | 51.7 | 10.9 | 13.3 | 9.9 | 24 | 471 | 19.63 |
| **Q2** | 1 | 62.5 | 7.2 | 9.1 | 6.9 | 30 | 322 | 10.73 |
| **Q2** | 2 | 70 | 7.7 | 10.1 | 7.4 | 44 | 741 | 16.84 |
| **Q2** | 3 | 69.7 | 7.5 | 9.5 | 7 | 36 | 575 | 15.97 |
| **Q2** | 4 | 72.5 | 6.2 | 8.3 | 6.2 | 52 | 638 | 12.27 |
| **Q2** | 5 | 56 | 10.9 | 13 | 9.4 | 27 | 590 | 21.85 |
| **Q2** | 6 | 57.4 | 8.5 | 10.4 | 7.9 | 22 | 287 | 13.05 |
| **Q2** | 7 | 69.3 | 7.2 | 9.4 | 6.8 | 33 | 485 | 14.7 |
| **Q2** | 8 | 65.7 | 7.2 | 9.5 | 7.2 | 22 | 279 | 12.68 |
| **Q2** | 9 | 63.8 | 8.7 | 10.8 | 7.9 | 33 | 575 | 17.42 |
| **Q2** | 10 | 64.9 | 7.6 | 10 | 7.8 | 23 | 310 | 13.48 |
| **Q3** | 1 | 55.5 | 9.6 | 12.2 | 9.2 | 40 | 666 | 16.65 |
| **Q3** | 2 | 57.3 | 9 | 11.1 | 8.8 | 40 | 601 | 15.03 |
| **Q3** | 3 | 48.3 | 9.4 | 10.9 | 8.5 | 52 | 611 | 11.75 |
| **Q3** | 4 | 56 | 8.8 | 11 | 8.5 | 50 | 683 | 13.66 |
| **Q3** | 5 | 54.4 | 9.7 | 11.4 | 9.3 | 43 | 697 | 16.21 |
| **Q3** | 6 | 51.9 | 10.1 | 12.4 | 9.6 | 50 | 828 | 16.56 |
| **Q3** | 7 | 49.7 | 10.7 | 13.4 | 10.3 | 47 | 832 | 17.7 |
| **Q3** | 8 | 44.5 | 11.2 | 13.5 | 10.5 | 50 | 830 | 16.6 |
| **Q3** | 9 | 50.2 | 10.2 | 12.6 | 9.9 | 52 | 815 | 15.67 |
| **Q3** | 10 | 53.8 | 8.4 | 10.5 | 8 | 42 | 453 | 10.79 |
| **Q4** | 1 | 61.4 | 8 | 9.7 | 7.2 | 43 | 574 | 13.35 |
| **Q4** | 2 | 63.7 | 7.7 | 10 | 7.4 | 36 | 488 | 13.56 |
| **Q4** | 3 | 68.3 | 7 | 9.2 | 6.9 | 55 | 724 | 13.16 |
| **Q4** | 4 | 66 | 7.3 | 10 | 7.4 | 38 | 498 | 13.11 |
| **Q4** | 5 | 71.6 | 6.2 | 8.4 | 6.3 | 41 | 477 | 11.63 |
| **Q4** | 6 | 66.7 | 7.6 | 9.6 | 7 | 42 | 616 | 14.67 |
| **Q4** | 7 | 69.1 | 6.3 | 8.5 | 6.4 | 41 | 438 | 10.68 |
| **Q4** | 8 | 67.2 | 7.3 | 10 | 7.4 | 39 | 528 | 13.54 |
| **Q4** | 9 | 68.1 | 6.6 | 8.5 | 6.4 | 51 | 577 | 11.31 |
| **Q4** | 10 | 76.4 | 5 | 7.4 | 5.7 | 50 | 487 | 9.74 |
| **Q5** | 1 | 72.9 | 6.3 | 8.9 | 6.6 | 37 | 475 | 12.84 |
| **Q5** | 2 | 71.3 | 6.4 | 9 | 6.7 | 48 | 593 | 12.35 |
| **Q5** | 3 | 76.5 | 6.1 | 8.6 | 6.2 | 28 | 392 | 14 |
| **Q5** | 4 | 64.6 | 7.9 | 10.8 | 8 | 30 | 442 | 14.73 |
| **Q5** | 5 | 65.6 | 7.3 | 10.1 | 7.5 | 46 | 596 | 12.96 |
| **Q5** | 6 | 59.7 | 8.6 | 11.8 | 8.7 | 30 | 445 | 14.83 |
| **Q5** | 7 | 67.2 | 6.6 | 9.2 | 6.9 | 56 | 621 | 11.09 |
| **Q5** | 8 | 65.4 | 8 | 11.4 | 8.4 | 36 | 561 | 15.58 |
| **Q5** | 9 | 72.1 | 6.2 | 8.5 | 6.6 | 53 | 646 | 12.19 |
| **Q5** | 10 | 70.9 | 6.9 | 9.5 | 7 | 40 | 561 | 14.03 |
| **Q6** | 1 | 58.5 | 8 | 10.5 | 7.8 | 32 | 380 | 11.88 |
| **Q6** | 2 | 57.5 | 8.8 | 11.6 | 8.5 | 32 | 456 | 14.25 |
| **Q6** | 3 | 56.1 | 9.9 | 13.2 | 9.6 | 27 | 480 | 17.78 |
| **Q6** | 4 | 64.5 | 7.5 | 10.3 | 7.7 | 45 | 583 | 12.96 |
| **Q6** | 5 | 57.4 | 9.6 | 13.1 | 9.5 | 28 | 491 | 17.54 |
| **Q6** | 6 | 58 | 8.6 | 11.4 | 8.5 | 43 | 599 | 13.93 |
| **Q6** | 7 | 59.1 | 8.6 | 11.7 | 8.6 | 43 | 629 | 14.63 |
| **Q6** | 8 | 57.3 | 8.9 | 11.3 | 8.4 | 24 | 351 | 14.63 |
| **Q6** | 9 | 58.2 | 9.3 | 12.1 | 8.9 | 26 | 435 | 16.73 |
| **Q6** | 10 | 60.5 | 8.7 | 11.3 | 8.5 | 30 | 471 | 15.7 |
| **Q7** | 1 | 59 | 8.2 | 11 | 8.4 | 50 | 644 | 12.88 |
| **Q7** | 2 | 59.9 | 8.2 | 10.7 | 8.3 | 50 | 655 | 13.1 |
| **Q7** | 3 | 63.9 | 7.2 | 9.9 | 7.6 | 55 | 630 | 11.45 |
| **Q7** | 4 | 63.3 | 7.6 | 9.9 | 7.8 | 54 | 699 | 12.94 |
| **Q7** | 5 | 62.9 | 7.3 | 10 | 7.8 | 46 | 527 | 11.46 |
| **Q7** | 6 | 63.7 | 7.8 | 10.2 | 7.8 | 51 | 696 | 13.65 |
| **Q7** | 7 | 53.3 | 9.8 | 12.2 | 9.1 | 38 | 601 | 15.82 |
| **Q7** | 8 | 65.8 | 7.4 | 9.7 | 7.8 | 55 | 733 | 13.33 |
| **Q7** | 9 | 64.3 | 7.4 | 10.1 | 7.7 | 53 | 653 | 12.32 |
| **Q7** | 10 | 58.6 | 8.3 | 11 | 8.4 | 54 | 707 | 13.09 |
| **Q8** | 1 | 49.7 | 10 | 13 | 9.9 | 44 | 652 | 14.82 |
| **Q8** | 2 | 50.1 | 9 | 12.5 | 8.9 | 65 | 720 | 11.08 |
| **Q8** | 3 | 45 | 11.7 | 14.8 | 11.3 | 37 | 709 | 19.16 |
| **Q8** | 4 | 45.6 | 9.4 | 12.5 | 8.8 | 72 | 734 | 10.19 |
| **Q8** | 5 | 51.3 | 9.3 | 11.9 | 8.9 | 40 | 520 | 13 |
| **Q8** | 6 | 51.7 | 9.4 | 12.6 | 9.3 | 54 | 725 | 13.43 |
| **Q8** | 7 | 50.3 | 9.8 | 12.2 | 9.4 | 54 | 779 | 14.43 |
| **Q8** | 8 | 47.2 | 10.3 | 13 | 9.8 | 45 | 650 | 14.44 |
| **Q8** | 9 | 39.2 | 12.4 | 15 | 11.5 | 36 | 661 | 18.36 |
| **Q8** | 10 | 48.6 | 10 | 13.1 | 9.8 | 46 | 651 | 14.15 |
| **Q9** | 1 | 59.5 | 8.3 | 10.3 | 7.8 | 40 | 537 | 13.43 |
| **Q9** | 2 | 57.9 | 9.3 | 11.8 | 8.7 | 26 | 434 | 16.69 |
| **Q9** | 3 | 60.7 | 8.3 | 10.3 | 7.6 | 35 | 495 | 14.14 |
| **Q9** | 4 | 61.2 | 7.6 | 10 | 7.4 | 32 | 369 | 11.53 |
| **Q9** | 5 | 57.9 | 9.1 | 11.3 | 8.3 | 27 | 422 | 15.63 |
| **Q9** | 6 | 57.4 | 8.9 | 11.2 | 8.3 | 37 | 541 | 14.62 |
| **Q9** | 7 | 63.9 | 7.7 | 9.9 | 7.3 | 33 | 443 | 13.42 |
| **Q9** | 8 | 63.3 | 8.1 | 10 | 7.4 | 32 | 468 | 14.63 |
| **Q9** | 9 | 65.9 | 7.2 | 9.2 | 7.1 | 34 | 432 | 12.71 |
| **Q9** | 10 | 63.2 | 7.2 | 9.4 | 7.1 | 38 | 424 | 11.16 |
| **Q10** | 1 | 53.5 | 9.8 | 12.2 | 9.3 | 44 | 716 | 16.27 |
| **Q10** | 2 | 49.1 | 10.4 | 13.6 | 10.1 | 27 | 435 | 16.11 |
| **Q10** | 3 | 49 | 9.1 | 12.1 | 8.7 | 51 | 544 | 10.67 |
| **Q10** | 4 | 45.3 | 11.2 | 14.5 | 10.5 | 15 | 257 | 17.13 |
| **Q10** | 5 | 55.5 | 9.3 | 12.3 | 9.2 | 40 | 614 | 15.35 |
| **Q10** | 6 | 56.2 | 8.8 | 11.9 | 8.8 | 50 | 674 | 13.48 |
| **Q10** | 7 | 44.4 | 10.6 | 13.6 | 9.9 | 43 | 604 | 14.05 |
| **Q10** | 8 | 47.9 | 9.7 | 12.4 | 8.9 | 34 | 424 | 12.47 |
| **Q10** | 9 | 51.4 | 9.3 | 11.7 | 8.6 | 41 | 533 | 13 |
| **Q10** | 10 | 58.5 | 8.7 | 11.9 | 8.8 | 44 | 636 | 14.45 |
| **Q11** | 1 | 51.8 | 9.1 | 12.1 | 8.8 | 33 | 411 | 12.45 |
| **Q11** | 2 | 55.2 | 9.1 | 11.5 | 8.4 | 56 | 802 | 14.32 |
| **Q11** | 3 | 54.1 | 8.4 | 10 | 7.7 | 76 | 809 | 10.64 |
| **Q11** | 4 | 57.5 | 8.7 | 11 | 8.3 | 59 | 813 | 13.78 |
| **Q11** | 5 | 56 | 8.4 | 10.5 | 7.8 | 61 | 718 | 11.77 |
| **Q11** | 6 | 50.5 | 9.1 | 11.5 | 8.5 | 66 | 765 | 11.59 |
| **Q11** | 7 | 54.9 | 8.7 | 11 | 8.3 | 63 | 797 | 12.65 |
| **Q11** | 8 | 53.7 | 8.9 | 11.5 | 8.6 | 72 | 922 | 12.81 |
| **Q11** | 9 | 54.9 | 8.6 | 11.6 | 8.5 | 56 | 680 | 12.14 |
| **Q11** | 10 | 54.4 | 8.6 | 10.7 | 8 | 57 | 669 | 11.74 |
| **Q12** | 1 | 62.5 | 7.6 | 9.5 | 7.2 | 43 | 520 | 12.09 |
| **Q12** | 2 | 65 | 7.5 | 9.7 | 7.3 | 53 | 704 | 13.28 |
| **Q12** | 3 | 67.7 | 7 | 9.1 | 7 | 42 | 543 | 12.93 |
| **Q12** | 4 | 62.9 | 8.2 | 10.4 | 7.8 | 36 | 535 | 14.86 |
| **Q12** | 5 | 61.3 | 8.3 | 10.4 | 7.7 | 25 | 365 | 14.6 |
| **Q12** | 6 | 60.5 | 8.9 | 10.8 | 7.8 | 30 | 497 | 16.57 |
| **Q12** | 7 | 55.5 | 10.1 | 12.4 | 9.2 | 24 | 439 | 18.29 |
| **Q12** | 8 | 61.3 | 8 | 10.4 | 7.7 | 43 | 572 | 13.3 |
| **Q12** | 9 | 71 | 6.7 | 9 | 6.7 | 45 | 606 | 13.47 |
| **Q12** | 10 | 62.8 | 7.6 | 9.9 | 7.4 | 39 | 492 | 12.62 |
| **Q13** | 1 | 54.4 | 9.6 | 11.7 | 9 | 41 | 642 | 15.66 |
| **Q13** | 2 | 61.5 | 8.1 | 10.4 | 8.2 | 60 | 830 | 13.83 |
| **Q13** | 3 | 54.2 | 10.2 | 12 | 9.2 | 32 | 573 | 17.91 |
| **Q13** | 4 | 56.1 | 9.2 | 11.7 | 8.9 | 45 | 689 | 15.31 |
| **Q13** | 5 | 56.5 | 9.1 | 11.1 | 8.7 | 39 | 586 | 15.03 |
| **Q13** | 6 | 58.5 | 8.6 | 10.5 | 8.2 | 51 | 728 | 14.27 |
| **Q13** | 7 | 59.1 | 8.6 | 10.7 | 8.2 | 48 | 700 | 14.58 |
| **Q13** | 8 | 56.4 | 9.5 | 11.6 | 8.8 | 40 | 660 | 16.5 |
| **Q13** | 9 | 61.5 | 7.2 | 9.3 | 7.1 | 64 | 643 | 10.05 |
| **Q13** | 10 | 60.6 | 8 | 10.2 | 8.1 | 64 | 820 | 12.81 |
| **Q14** | 1 | 48.5 | 10 | 12.5 | 9.1 | 29 | 407 | 14.03 |
| **Q14** | 2 | 42.3 | 11.9 | 14.8 | 10.9 | 25 | 454 | 18.16 |
| **Q14** | 3 | 56.9 | 8.8 | 11.4 | 8.5 | 35 | 494 | 14.11 |
| **Q14** | 4 | 53.1 | 8.9 | 12.3 | 8.8 | 45 | 546 | 12.13 |
| **Q14** | 5 | 56.9 | 9.2 | 12.3 | 9.1 | 24 | 372 | 15.5 |
| **Q14** | 6 | 60.1 | 8.9 | 11.9 | 8.8 | 24 | 386 | 16.08 |
| **Q14** | 7 | 52.6 | 10.1 | 12.4 | 9.1 | 21 | 355 | 16.9 |
| **Q14** | 8 | 54.1 | 8.8 | 11.4 | 8.5 | 32 | 398 | 12.44 |
| **Q14** | 9 | 57.9 | 8.2 | 11 | 8 | 33 | 396 | 12 |
| **Q14** | 10 | 51.5 | 9.6 | 12.4 | 9.2 | 30 | 425 | 14.17 |
| **Q15** | 1 | 49.5 | 10.2 | 13 | 9.6 | 48 | 736 | 15.33 |
| **Q15** | 2 | 37.9 | 10.9 | 12.9 | 9.8 | 60 | 716 | 11.93 |
| **Q15** | 3 | 33.3 | 11.8 | 14.6 | 10.2 | 19 | 247 | 13 |
| **Q15** | 4 | 48 | 10.7 | 13.3 | 10.2 | 45 | 745 | 16.56 |
| **Q15** | 5 | 37.9 | 12.4 | 14.7 | 11 | 25 | 442 | 17.68 |
| **Q15** | 6 | 27.8 | 13.5 | 17.2 | 12.1 | 12 | 201 | 16.75 |
| **Q15** | 7 | 47.3 | 9.3 | 11.9 | 8.7 | 36 | 376 | 10.44 |
| **Q15** | 8 | 50 | 8.8 | 11.1 | 8.2 | 49 | 492 | 10.04 |
| **Q15** | 9 | 42.1 | 11.1 | 13.9 | 10.3 | 40 | 594 | 14.85 |
| **Q15** | 10 | 49 | 9.9 | 12.7 | 9.7 | 22 | 312 | 14.18 |

### **S2.3.5 Doubao (n = 150)**

| **PromptID** | **Run** | **FRE** | **FKGL** | **Gunning Fog** | **SMOG** | **Sentences** | **Words** | **Words / sentence** |
| --- | --- | --- | --- | --- | --- | --- | --- | --- |
| **Q1** | 1 | 66 | 8 | 9.7 | 7.4 | 9 | 144 | 16 |
| **Q1** | 2 | 59.9 | 10.2 | 12 | 8.5 | 23 | 491 | 21.35 |
| **Q1** | 3 | 68.5 | 6.6 | 9.2 | 6.9 | 32 | 380 | 11.88 |
| **Q1** | 4 | 67 | 6.6 | 9.1 | 7.1 | 31 | 334 | 10.77 |
| **Q1** | 5 | 69.5 | 6.7 | 8.1 | 6.5 | 34 | 428 | 12.59 |
| **Q1** | 6 | 70.8 | 5.8 | 8 | 6.3 | 46 | 454 | 9.87 |
| **Q1** | 7 | 74.5 | 5.2 | 8.4 | 6.2 | 25 | 232 | 9.28 |
| **Q1** | 8 | 68.7 | 6.7 | 8.4 | 6.8 | 31 | 378 | 12.19 |
| **Q1** | 9 | 72.4 | 5.9 | 8.2 | 6.5 | 39 | 432 | 11.08 |
| **Q1** | 10 | 69 | 6.2 | 8.2 | 6.6 | 36 | 377 | 10.47 |
| **Q2** | 1 | 53.4 | 11.7 | 14 | 10.7 | 14 | 329 | 23.5 |
| **Q2** | 2 | 68.8 | 6.1 | 7.9 | 6.1 | 32 | 308 | 9.63 |
| **Q2** | 3 | 62.6 | 9 | 10.4 | 8.2 | 22 | 393 | 17.86 |
| **Q2** | 4 | 75.3 | 4.5 | 6.3 | 5.2 | 59 | 401 | 6.8 |
| **Q2** | 5 | 63.7 | 7.2 | 8.8 | 6.9 | 27 | 306 | 11.33 |
| **Q2** | 6 | 62.5 | 9.2 | 10.5 | 8.1 | 17 | 321 | 18.88 |
| **Q2** | 7 | 68 | 5.7 | 8.1 | 5.9 | 39 | 305 | 7.82 |
| **Q2** | 8 | 65.7 | 6.6 | 8.8 | 6.9 | 32 | 319 | 9.97 |
| **Q2** | 9 | 73.3 | 5.4 | 7.2 | 5.9 | 42 | 392 | 9.33 |
| **Q2** | 10 | 53.6 | 9.3 | 11.3 | 8.6 | 6 | 86 | 14.33 |
| **Q3** | 1 | 50.7 | 11.4 | 12.8 | 10.1 | 17 | 354 | 20.82 |
| **Q3** | 2 | 47 | 10.2 | 12.1 | 9.7 | 24 | 339 | 14.13 |
| **Q3** | 3 | 44.2 | 10.6 | 12.5 | 9.9 | 17 | 239 | 14.06 |
| **Q3** | 4 | 46.6 | 10.1 | 12.8 | 9.7 | 20 | 269 | 13.45 |
| **Q3** | 5 | 40.9 | 11.5 | 13.9 | 10.9 | 28 | 445 | 15.89 |
| **Q3** | 6 | 50.5 | 9.4 | 10.7 | 8.8 | 22 | 286 | 13 |
| **Q3** | 7 | 40.4 | 13.8 | 14.8 | 12.1 | 11 | 274 | 24.91 |
| **Q3** | 8 | 31.3 | 15 | 16.9 | 13.3 | 17 | 420 | 24.71 |
| **Q3** | 9 | 44.5 | 10.2 | 11.5 | 9.6 | 31 | 391 | 12.61 |
| **Q3** | 10 | 41.3 | 11.7 | 13.9 | 10.8 | 15 | 255 | 17 |
| **Q4** | 1 | 54.7 | 11.4 | 13.5 | 9.4 | 20 | 464 | 23.2 |
| **Q4** | 2 | 64 | 7.6 | 9.8 | 7.3 | 35 | 462 | 13.2 |
| **Q4** | 3 | 61.6 | 8.3 | 10.4 | 7.9 | 28 | 411 | 14.68 |
| **Q4** | 4 | 63.5 | 7.9 | 10.3 | 7.6 | 38 | 530 | 13.95 |
| **Q4** | 5 | 68.1 | 7.6 | 9.8 | 7.2 | 33 | 503 | 15.24 |
| **Q4** | 6 | 64.2 | 8.3 | 10 | 7.7 | 26 | 415 | 15.96 |
| **Q4** | 7 | 59.8 | 10.1 | 12.9 | 9 | 23 | 476 | 20.7 |
| **Q4** | 8 | 57.5 | 10.3 | 12.7 | 9.1 | 22 | 446 | 20.27 |
| **Q4** | 9 | 51 | 11.6 | 13.3 | 9.9 | 23 | 504 | 21.91 |
| **Q4** | 10 | 65.3 | 7.9 | 10.1 | 7.5 | 26 | 396 | 15.23 |
| **Q5** | 1 | 75.1 | 6.2 | 8.4 | 6.4 | 20 | 276 | 13.8 |
| **Q5** | 2 | 85.6 | 4 | 5.8 | 5 | 20 | 219 | 10.95 |
| **Q5** | 3 | 77.8 | 6.6 | 9 | 6.5 | 16 | 270 | 16.88 |
| **Q5** | 4 | 67.7 | 8.6 | 11.4 | 8.3 | 16 | 306 | 19.13 |
| **Q5** | 5 | 72.5 | 7.4 | 10.1 | 7.7 | 14 | 242 | 17.29 |
| **Q5** | 6 | 71.9 | 6.8 | 9.4 | 7.1 | 23 | 328 | 14.26 |
| **Q5** | 7 | 72.9 | 9 | 11.5 | 7.4 | 12 | 287 | 23.92 |
| **Q5** | 8 | 66 | 8.4 | 11.1 | 8.1 | 14 | 243 | 17.36 |
| **Q5** | 9 | 74.1 | 6.6 | 9.1 | 6.9 | 22 | 323 | 14.68 |
| **Q5** | 10 | 62.1 | 10.5 | 12.7 | 8.8 | 8 | 191 | 23.88 |
| **Q6** | 1 | 63.3 | 7.4 | 9.9 | 7.8 | 28 | 329 | 11.75 |
| **Q6** | 2 | 65.2 | 7.5 | 11.2 | 8.4 | 25 | 338 | 13.52 |
| **Q6** | 3 | 56.5 | 9.9 | 12.5 | 9.4 | 22 | 399 | 18.14 |
| **Q6** | 4 | 57.8 | 9.2 | 11.6 | 8.6 | 22 | 351 | 15.95 |
| **Q6** | 5 | 66.5 | 7.3 | 9.9 | 7.5 | 31 | 410 | 13.23 |
| **Q6** | 6 | 64.9 | 7.6 | 10.6 | 7.9 | 23 | 316 | 13.74 |
| **Q6** | 7 | 47.5 | 12.2 | 13.8 | 11.1 | 14 | 314 | 22.43 |
| **Q6** | 8 | 45.1 | 12.1 | 14.9 | 11 | 14 | 289 | 20.64 |
| **Q6** | 9 | 53.7 | 11 | 13.6 | 10.2 | 18 | 381 | 21.17 |
| **Q6** | 10 | 55.8 | 10.2 | 12.7 | 9.6 | 17 | 320 | 18.82 |
| **Q7** | 1 | 49.2 | 9.4 | 11.1 | 8.8 | 32 | 392 | 12.25 |
| **Q7** | 2 | 49.4 | 10 | 12.1 | 9.5 | 32 | 470 | 14.69 |
| **Q7** | 3 | 60.1 | 7.6 | 9.8 | 7.4 | 38 | 410 | 10.79 |
| **Q7** | 4 | 56.8 | 9 | 10.8 | 8.4 | 27 | 402 | 14.89 |
| **Q7** | 5 | 53.7 | 8.4 | 10.3 | 8 | 40 | 423 | 10.58 |
| **Q7** | 6 | 49.4 | 11.1 | 12.8 | 10.2 | 22 | 417 | 18.95 |
| **Q7** | 7 | 62.6 | 8.3 | 10.3 | 8.1 | 26 | 394 | 15.15 |
| **Q7** | 8 | 50 | 10.7 | 13 | 9.9 | 19 | 335 | 17.63 |
| **Q7** | 9 | 52.1 | 10.2 | 12.4 | 9.6 | 21 | 352 | 16.76 |
| **Q7** | 10 | 51.2 | 12 | 14.6 | 10.6 | 15 | 357 | 23.8 |
| **Q8** | 1 | 34.8 | 14.1 | 15.8 | 12.3 | 18 | 411 | 22.83 |
| **Q8** | 2 | 38.9 | 12.2 | 14.2 | 11 | 31 | 543 | 17.52 |
| **Q8** | 3 | 26.9 | 14.5 | 15.5 | 12.4 | 8 | 160 | 20 |
| **Q8** | 4 | 41.2 | 11.5 | 13.5 | 10.5 | 29 | 462 | 15.93 |
| **Q8** | 5 | 49.5 | 9.4 | 11.2 | 8.7 | 38 | 468 | 12.32 |
| **Q8** | 6 | 42.4 | 12 | 13.5 | 10.6 | 26 | 489 | 18.81 |
| **Q8** | 7 | 41.3 | 12.4 | 13.8 | 10.7 | 24 | 470 | 19.58 |
| **Q8** | 8 | 53.8 | 8.4 | 10.2 | 8 | 39 | 423 | 10.85 |
| **Q8** | 9 | 46.9 | 11.9 | 13.5 | 10.1 | 24 | 505 | 21.04 |
| **Q8** | 10 | 48.3 | 9.3 | 10.8 | 8.5 | 37 | 415 | 11.22 |
| **Q9** | 1 | 62.6 | 8.4 | 10.6 | 7.9 | 21 | 326 | 15.52 |
| **Q9** | 2 | 62.8 | 7.8 | 9.7 | 7.6 | 24 | 318 | 13.25 |
| **Q9** | 3 | 62.8 | 8.3 | 10.6 | 7.7 | 22 | 334 | 15.18 |
| **Q9** | 4 | 55.6 | 10.6 | 13.8 | 10 | 15 | 309 | 20.6 |
| **Q9** | 5 | 65.2 | 7.6 | 9.9 | 7.5 | 23 | 319 | 13.87 |
| **Q9** | 6 | 53.8 | 11 | 13.1 | 9.4 | 15 | 317 | 21.13 |
| **Q9** | 7 | 64.1 | 8 | 10.4 | 7.7 | 23 | 342 | 14.87 |
| **Q9** | 8 | 59.8 | 9.5 | 11.7 | 8.6 | 18 | 334 | 18.56 |
| **Q9** | 9 | 54.5 | 11.8 | 13.9 | 9.9 | 14 | 343 | 24.5 |
| **Q9** | 10 | 60.4 | 10 | 12.6 | 9.2 | 16 | 332 | 20.75 |
| **Q10** | 1 | 40.5 | 13.4 | 16.3 | 12.3 | 20 | 464 | 23.2 |
| **Q10** | 2 | 43.3 | 10 | 12.8 | 9.3 | 39 | 440 | 11.28 |
| **Q10** | 3 | 51.7 | 8.7 | 10.8 | 8.3 | 44 | 476 | 10.82 |
| **Q10** | 4 | 46.1 | 9.9 | 11.8 | 9 | 38 | 476 | 12.53 |
| **Q10** | 5 | 44.8 | 10.7 | 13.1 | 10 | 32 | 476 | 14.88 |
| **Q10** | 6 | 40.4 | 11.1 | 13.5 | 10.1 | 34 | 474 | 13.94 |
| **Q10** | 7 | 38.9 | 12.3 | 13.5 | 10.9 | 25 | 448 | 17.92 |
| **Q10** | 8 | 50.2 | 9.3 | 12 | 9.1 | 39 | 473 | 12.13 |
| **Q10** | 9 | 56.8 | 8.5 | 11.8 | 8.9 | 35 | 449 | 12.83 |
| **Q10** | 10 | 47 | 10.6 | 12.4 | 9.4 | 24 | 377 | 15.71 |
| **Q11** | 1 | 49.1 | 11.2 | 12.5 | 9.9 | 24 | 465 | 19.38 |
| **Q11** | 2 | 56 | 8.4 | 9.3 | 7.8 | 37 | 447 | 12.08 |
| **Q11** | 3 | 47.5 | 12 | 13.4 | 10.3 | 21 | 451 | 21.48 |
| **Q11** | 4 | 45.6 | 11.9 | 13.1 | 10.4 | 24 | 481 | 20.04 |
| **Q11** | 5 | 48.8 | 11.6 | 12.7 | 10.1 | 27 | 561 | 20.78 |
| **Q11** | 6 | 54.7 | 9.4 | 10.8 | 8.5 | 33 | 500 | 15.15 |
| **Q11** | 7 | 40.6 | 13.2 | 14.2 | 11.1 | 22 | 492 | 22.36 |
| **Q11** | 8 | 60.5 | 7.4 | 9.1 | 7.1 | 61 | 628 | 10.3 |
| **Q11** | 9 | 39.2 | 13.9 | 15.6 | 11.8 | 23 | 563 | 24.48 |
| **Q11** | 10 | 48.3 | 11.1 | 12.6 | 9.9 | 29 | 532 | 18.34 |
| **Q12** | 1 | 54.4 | 11.5 | 13.3 | 9.6 | 15 | 352 | 23.47 |
| **Q12** | 2 | 55.7 | 9.8 | 11.8 | 8.8 | 24 | 414 | 17.25 |
| **Q12** | 3 | 61.1 | 8.8 | 11.3 | 8.4 | 30 | 486 | 16.2 |
| **Q12** | 4 | 57.6 | 7.8 | 9.9 | 7.7 | 36 | 376 | 10.44 |
| **Q12** | 5 | 45 | 12.8 | 15.2 | 11.2 | 17 | 396 | 23.29 |
| **Q12** | 6 | 50 | 11.8 | 13.8 | 10.3 | 19 | 425 | 22.37 |
| **Q12** | 7 | 60 | 8.2 | 10.4 | 7.9 | 33 | 435 | 13.18 |
| **Q12** | 8 | 45.7 | 12.6 | 15.3 | 11.1 | 10 | 231 | 23.1 |
| **Q12** | 9 | 55.9 | 10 | 12.1 | 9.2 | 16 | 295 | 18.44 |
| **Q12** | 10 | 52 | 10.8 | 12.8 | 9.7 | 26 | 506 | 19.46 |
| **Q13** | 1 | 54.9 | 8.9 | 11 | 8.7 | 42 | 562 | 13.38 |
| **Q13** | 2 | 59.9 | 8.2 | 9.4 | 7.8 | 48 | 630 | 13.13 |
| **Q13** | 3 | 53.9 | 9.2 | 11.5 | 8.8 | 34 | 477 | 14.03 |
| **Q13** | 4 | 62.6 | 7.1 | 9.3 | 7.4 | 42 | 443 | 10.55 |
| **Q13** | 5 | 57.1 | 8.6 | 11 | 8.5 | 42 | 559 | 13.31 |
| **Q13** | 6 | 62.5 | 6.8 | 9 | 7.2 | 56 | 515 | 9.2 |
| **Q13** | 7 | 50.4 | 9.3 | 11.2 | 8.8 | 36 | 453 | 12.58 |
| **Q13** | 8 | 59.8 | 8 | 9.9 | 8 | 47 | 588 | 12.51 |
| **Q13** | 9 | 52 | 9.4 | 11.3 | 8.9 | 36 | 490 | 13.61 |
| **Q13** | 10 | 55.2 | 8.6 | 10.6 | 8.6 | 46 | 562 | 12.22 |
| **Q14** | 1 | 53.1 | 10.3 | 12.7 | 9.8 | 17 | 301 | 17.71 |
| **Q14** | 2 | 37.8 | 14.2 | 16.9 | 12.8 | 12 | 299 | 24.92 |
| **Q14** | 3 | 61 | 7.2 | 9.6 | 7.4 | 30 | 300 | 10 |
| **Q14** | 4 | 37.4 | 14.1 | 16.3 | 12.1 | 11 | 267 | 24.27 |
| **Q14** | 5 | 44.8 | 12 | 14.1 | 10.6 | 14 | 280 | 20 |
| **Q14** | 6 | 51.6 | 10 | 12.9 | 9.4 | 17 | 270 | 15.88 |
| **Q14** | 7 | 44.7 | 12.4 | 14.5 | 10.7 | 13 | 282 | 21.69 |
| **Q14** | 8 | 45.5 | 10.5 | 12.8 | 9.5 | 15 | 215 | 14.33 |
| **Q14** | 9 | 53.5 | 8.4 | 10.4 | 7.8 | 24 | 252 | 10.5 |
| **Q14** | 10 | 45.8 | 11.8 | 14 | 10.4 | 14 | 278 | 19.86 |
| **Q15** | 1 | 52.7 | 8.8 | 10.7 | 8.4 | 60 | 692 | 11.53 |
| **Q15** | 2 | 27.9 | 13.5 | 14.7 | 11.6 | 24 | 403 | 16.79 |
| **Q15** | 3 | 34.8 | 14.2 | 15.7 | 12.1 | 14 | 324 | 23.14 |
| **Q15** | 4 | 32.8 | 12.4 | 12.9 | 10.6 | 15 | 223 | 14.87 |
| **Q15** | 5 | 30.1 | 14.3 | 15.8 | 12.3 | 27 | 569 | 21.07 |
| **Q15** | 6 | 24.8 | 15.2 | 17.2 | 13.9 | 19 | 415 | 21.84 |
| **Q15** | 7 | 33.3 | 12.5 | 13.7 | 11.4 | 45 | 697 | 15.49 |
| **Q15** | 8 | 27.4 | 15.4 | 17.4 | 13.7 | 18 | 429 | 23.83 |
| **Q15** | 9 | 33.9 | 14.4 | 15.3 | 12.6 | 29 | 689 | 23.76 |
| **Q15** | 10 | 41 | 11.4 | 13.4 | 10.4 | 38 | 591 | 15.55 |

## **Table S2.4. Sensitivity of model-level readability estimates to sentence-boundary preprocessing**

## Values are model-level means across 150 responses per model. The primary analysis preserved independent list-item and table-row sentence boundaries. The structure-stripped sensitivity scenario reflects the earlier preprocessing approach in which these structural boundaries were not retained.

| **Model** | **Primary FRE** | **Primary FKGL** | **Primary Fog** | **Primary SMOG** | **Sensitivity FRE** | **Sensitivity FKGL** | **Sensitivity Fog** | **Sensitivity SMOG** |
| --- | --- | --- | --- | --- | --- | --- | --- | --- |
| **ChatGPT** | 56.57 | 8.60 | 10.97 | 8.34 | 24.84 | 19.68 | 21.84 | 14.44 |
| **Gemini** | 53.14 | 9.79 | 11.68 | 9.09 | 53.52 | 9.60 | 10.98 | 8.98 |
| **Claude** | 45.46 | 11.32 | 13.84 | 10.30 | 34.87 | 15.37 | 17.51 | 12.69 |
| **DeepSeek** | 57.83 | 8.72 | 11.21 | 8.38 | 58.40 | 8.85 | 11.12 | 8.46 |
| **Doubao** | 54.08 | 9.78 | 11.83 | 9.06 | 42.16 | 14.63 | 16.61 | 11.51 |

Values are model-level means across 150 responses per model. The primary analysis preserved independent list-item and table-row sentence boundaries. For sensitivity assessment, results from the earlier structure-stripped preprocessing, in which these boundaries were not retained, are shown for comparison.

The sensitivity comparison showed that model-level readability estimates were affected differently by preprocessing. Mean FRE for ChatGPT changed from 56.57 under the structure-preserving primary analysis to 24.84 under the structure-stripped scenario, whereas DeepSeek changed only from 57.83 to 58.40. The corresponding FRE ordering changed from DeepSeek > ChatGPT > Doubao > Gemini > Claude in the primary analysis to DeepSeek > Gemini > Doubao > Claude > ChatGPT in the structure-stripped scenario. Although DeepSeek remained above ChatGPT in both scenarios, the substantial change in ChatGPT's absolute FRE and the broader reordering across models demonstrate that readability estimates are sensitive to sentence-boundary preprocessing. Accordingly, small between-model differences in readability should not be interpreted as stable rankings across preprocessing choices.
