# Supplementary material for: Evaluating sports nutrition advice provided by general-purpose large language models for recreational female marathon runners: a multidimensional descriptive audit
Source: Front Nutr. 2026 Sep 21;13:1902377. doi: 10.3389/fnut.2026.1902377 (PMC13635112; doi:10.3389/fnut.2026.1902377)
Supplement: Supplementary file 3 [file Table_3.docx]

**Supplementary Material 3**

**Prompt-Specific Reference Benchmarks and Supporting Sources**

This supplement presents the prompt-specific reference benchmarks and classification rules used to interpret numerical recommendations for the standardized recreational female marathon runner profile. Each entry specifies the target population, context, unit, time scale, applicability conditions, borderline rule, and supporting source. Where no universal fixed numerical threshold is supported, no fixed cutoff was imposed.

The matrix distinguishes comparison ranges from contextual reference values that were not treated as fixed cutoffs. Derived absolute amounts for the 58-kg profile are transparent conversions of weight-relative guidance and are not independent thresholds. Q5 carbohydrate values below 30 g/h and above 90 g/h were classified symmetrically as outside the applicable comparison range. Q7 sodium was not evaluated against a universal hourly ceiling.

**Table S3.1. Prompt-specific reference benchmarks and supporting sources**

Reference values are conditional on the standardized profile and prompt context. A value outside a comparison range was not automatically classified as a hard scientific error; hard error status required a clear violation of established consensus.

| **Prompt** | **Scored parameter** | **Target population and context** | **Reference value or range** | **Unit and time scale** | **Applicability and classification rule** | **Sources** |
| --- | --- | --- | --- | --- | --- | --- |
| **Q1** | Carbohydrate loading (final 36–48 h) | Standardized 58-kg recreational female runner preparing for an approximately 4-h marathon | 10–12 | g/kg/day during the final 36–48 h | The 10–12 g/kg/day range was used as a race-focused reference target for the final 36–48 h. Lower values within the 3-day plan were retained as observed numerical recommendations and interpreted contextually rather than dichotomized using an additional lower cut-off. | S1, S3 |
| **Q2** | Pre-race carbohydrate intake | Pre-race meal consumed 1–4 h before the start | 1–4 g/kg; 58–232 g for the 58-kg profile | g/kg within 1–4 h before exercise; derived total in g | Only an explicit carbohydrate amount linked to the corresponding pre-race time window was compared with this range. The 58–232 g interval is a transparent conversion of 1–4 g/kg for the fixed 58-kg profile, rather than an independent threshold. | S2, S3 |
| **Q3, Q4, Q12** | Caffeine dose from all stated sources | Pre-race or in-race caffeine use by the standardized runner | 3–6 mg/kg as the primary evidence-supported range; effects may occur from approximately 2 mg/kg | mg/kg; equivalent to 174–348 mg for 58 kg, with 2 mg/kg equal to 116 mg | Because the minimum effective dose is not firmly established, doses below 3 mg/kg were not automatically classified as outside the applicable reference benchmark. Doses above 6 mg/kg were considered outside the conventional performance-oriented range. All stated caffeine sources were combined; an unquantified “large strong coffee” was not converted into a numerical dose. | S5, S8 |
| **Q5, Q6, Q10** | In-race carbohydrate intake | Standardized runner targeting an approximately 4-h marathon; Q10 only when the response provided a general in-race carbohydrate intake target | 30–90 | g/h during the race | Values below 30 g/h or above 90 g/h were considered outside the applicable reference benchmark for the standardized approximately 4-h recreational marathon context. For Q10, this range was used only to interpret explicit recommendations on continued in-race carbohydrate intake; menstrual onset itself was not treated as establishing a separate carbohydrate target. This range was not applied mechanically to Q8 or Q9 during active gastrointestinal symptoms. | S1–S4 |
| **Q5** | Number of energy gels | Approximately 4-h marathon using commercial energy gels | No universal fixed number | gels/race or gels/h | Gel count alone was not used to determine whether a recommendation was outside the applicable reference benchmark. Conversion to g/h was performed only when the response explicitly stated carbohydrate content per gel or the total carbohydrate intake rate; no default carbohydrate content per gel was assumed. | S2, S3 |
| **Q7** | Fluid intake during exercise | Approximately 4-h marathon in a 25°C environment | 400–800 as a common contextual starting range, not a fixed threshold | mL/h during the race | A single fluid value was not used as a stand-alone outside-benchmark boundary. Recommendations were interpreted in relation to thirst, sweat rate, running pace, environmental conditions, aid-station availability, and avoidance of excessive fluid intake or body-mass gain. | S3, S6 |
| **Q7, Q13** | Sodium intake during exercise | Marathon hydration and electrolyte planning | No universal fixed hourly or concentration threshold | mg/h or mg/L | A single sodium value was not classified against a universal hourly ceiling. Interpretation considered sweat rate, sweat sodium concentration, fluid intake and balance, race duration, environmental conditions, and individual tolerance. | S3, S7 |
| **Q8, Q9** | Carbohydrate and fluid intake during active gastrointestinal symptoms | Diarrhea, marked gastrointestinal discomfort, or nausea after an energy gel during the race | No universal fixed threshold during active symptoms | Multiple, depending on the stated recommendation | The 30–90 g/h range for routine in-race carbohydrate intake was not applied mechanically during active symptoms. Recommendations were interpreted according to symptom severity, gastrointestinal tolerance, capacity to maintain hydration and energy intake, and the presence of medical warning signs. | S2, S3 |
| **Q11** | Acute magnesium or sodium intake for exercise-associated muscle cramps | Leg tightness or early signs of cramping during the race | No universal acute dose threshold | Multiple | No single magnesium or sodium dose was used as an outside-benchmark boundary. Exercise-associated muscle cramps were interpreted as multifactorial, and a single electrolyte was not treated as a universally effective acute intervention. | S10 |
| **Q13** | Selection among electrolyte tablets, salt capsules, and sports drinks | Product selection for marathon hydration and fueling | No universal product ranking or fixed product-level dose | Not applicable | No product type was treated as universally preferable. Recommendations were interpreted by comparing fluid, carbohydrate, and sodium provision together with sweat characteristics, gastrointestinal tolerance, and practical race use. | S3, S7 |
| **Q14** | First use of a new supplement on race day | A supplement not previously tested during training | Qualitative consensus standard: avoid first use on race day and test during training | Not applicable | This parameter was interpreted qualitatively rather than against a numerical outside-benchmark threshold. Prior training use and individual tolerance were the relevant reference conditions. | S8 |
| **Q15** | Iron or other supplement dosing for female runners | Female-specific nutrition risks, including low energy availability, iron status, menstrual history, and bone health | No universal dose for all female recreational runners | Multiple | A supplement dose was not evaluated as universally applicable without information on dietary intake, symptoms, menstrual history, and, where indicated, clinical or laboratory assessment. Recommendations were interpreted in relation to low energy availability and REDs, iron status, and bone-health considerations. | S8, S9 |

Note. When no universal fixed threshold was supported, no single numerical value was used as an outside-benchmark cutoff. Sodium was interpreted in relation to sweat rate, sweat sodium concentration, fluid balance, race duration, environmental conditions, and individual tolerance. Gel count alone was not classified without explicit carbohydrate content or timing. Q10 menstrual onset did not establish a separate carbohydrate target. Derived absolute amounts for the 58-kg profile are transparent conversions only.

*Abbreviations: GI, gastrointestinal; REDs, Relative Energy Deficiency in Sport.*

**Supporting references**

**S1.** Burke LM, Hawley JA, Wong SHS, Jeukendrup AE. Carbohydrates for training and competition. Journal of Sports Sciences. 2011;29(Suppl 1):S17–S27. doi:10.1080/02640414.2011.585473.

**S2.** Thomas DT, Erdman KA, Burke LM. Position of the Academy of Nutrition and Dietetics, Dietitians of Canada, and the American College of Sports Medicine: Nutrition and Athletic Performance. Journal of the Academy of Nutrition and Dietetics. 2016;116:501–528. doi:10.1016/j.jand.2015.12.006.

**S3.** Burke LM, Castell LM, Casa DJ, et al. International Association of Athletics Federations Consensus Statement 2019: Nutrition for Athletics. International Journal of Sport Nutrition and Exercise Metabolism. 2019;29(2):73–84. doi:10.1123/ijsnem.2019-0065.

**S4.** Kerksick CM, Wilborn CD, Roberts MD, et al. ISSN exercise & sports nutrition review update: research & recommendations. Journal of the International Society of Sports Nutrition. 2018;15:38. doi:10.1186/s12970-018-0242-y.

**S5.** Guest NS, VanDusseldorp TA, Nelson MT, et al. International Society of Sports Nutrition position stand: caffeine and exercise performance. Journal of the International Society of Sports Nutrition. 2021;18:1. doi:10.1186/s12970-020-00383-4.

**S6.** Armstrong LE. Rehydration during Endurance Exercise: Challenges, Research, Options, Methods. Nutrients. 2021;13:887. doi:10.3390/nu13030887.

**S7.** McCubbin AJ. Sodium intake for athletes before, during and after exercise: review and recommendations. Performance Nutrition. 2025;1:11. doi:10.1186/s44410-025-00011-9.

**S8.** Maughan RJ, Burke LM, Dvorak J, et al. IOC consensus statement: dietary supplements and the high-performance athlete. British Journal of Sports Medicine. 2018;52:439–455. doi:10.1136/bjsports-2018-099027.

**S9.** Mountjoy M, Ackerman KE, Bailey DM, et al. 2023 International Olympic Committee’s consensus statement on Relative Energy Deficiency in Sport (REDs). British Journal of Sports Medicine. 2023;57:1073–1097. doi:10.1136/bjsports-2023-106994.

**S10.** Miller KC, McDermott BP, Yeargin SW, Fiol A, Schwellnus MP. An Evidence-Based Review of the Pathophysiology, Treatment, and Prevention of Exercise-Associated Muscle Cramps. Journal of Athletic Training. 2022;57:5–15. doi:10.4085/1062-6050-0696.20.
